# Merging Double Hydrogen Atom Transfer and Stepwise Proton-Coupled Electron Transfer for γ‑C–H Hydrazination of Alcohols

**DOI:** 10.1021/jacsau.5c01435

**Published:** 2026-01-26

**Authors:** Kaiming Zuo, Phong Dam, Kosala N. Amarasinghe, Anke Spannenberg, Jabor Rabeah, Olga S. Bokareva, Luis Miguel Azofra, Osama El-Sepelgy

**Affiliations:** † Leibniz Institute for Catalysis e.V., Albert-Einstein-Str. 29a, Rostock 18059, Germany; ‡ Instituto de Estudios Ambientales y Recursos Naturales (i-UNAT), Universidad de Las Palmas de Gran Canaria (ULPGC), Campus de Tafira, Las Palmas de Gran Canaria 35017, Spain; § Institute of Chemistry and Department of Life, Light & Matter, 9187 University of Rostock, Albert-Einstein-Str. 25 and 27, Rostock 18059, Germany

**Keywords:** cobalt, C−H functionalization, MHAT, radical relay, mechanistic study

## Abstract

A cobalt­(salen)-catalyzed γ-C–H hydrazination of alcohols is unveiled, merging a double hydrogen atom transfer (HAT) and a proton-coupled electron transfer (PCET) within a single catalytic cycle. The transformation harnesses metal–hydrogen atom transfer-induced radical translocation and electrophilic azodicarboxylate coupling to achieve remote C–H functionalization under mild and sustainable conditions. Mechanistic investigations (EPR, UV–vis, and spin-trapping) reveal cobalt oxidation state modulation and transient radical intermediates, while DFT analysis elucidates the double HAT/PCET pathway and site selectivity. This strategy offers an efficient and sustainable route from simple alcohols to γ-hydrazino and γ-amino alcohols.

## Graphical abstract

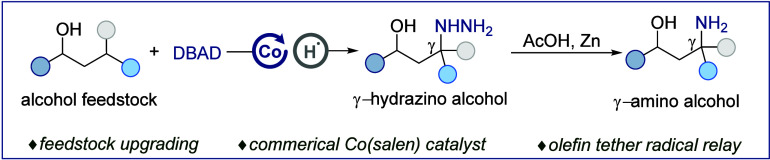

## Introduction

Amino alcohols represent privileged structural motifs found in numerous natural products and biologically active compounds. They play versatile roles across chemistry and medicine, serving not only as key components in small-molecule therapeutics but also as valuable chiral auxiliaries and ligands in asymmetric synthesis.^1–5^ Similarly, hydrazino alcohols constitute valuable scaffolds owing to their distinctive steric and electronic features, which enable the synthesis of azacycles, heterocycles, and diverse bioactive pharmacophores.^6,7^ Notably, replacing an amino group with a hydrazino moiety within a drug scaffold can profoundly alter its pharmacological properties. For instance, substitution of the amino group in the antihypertensive drug methyldopa^8^ with a hydrazino moiety yields carbidopa, an antiparkinsonian agent.^9,10^

While numerous methods have been reported for the synthesis of β- and δ-amino and hydrazino alcohols,^11–14^ general strategies for γ-amino alcohols remain scarce,^15–18^ and access to γ-hydrazino alcohols is even more challenging. Existing strategies for γ-amino alcohols typically involve multistep sequences with a limited substrate scope, harsh conditions, and poor regioselectivity, underscoring the need for efficient direct approaches that provide access to γ-hydrazino alcohols and, subsequently, to γ-amino alcohols. Remote C–H functionalization provides a powerful and conceptually distinct strategy for the direct molecular editing of abundant alcohol feedstocks^19–21^ into high-value products using radical strategy.^22–29^ A well-established strategy for remote γ-amination of alcohols relies on transition-metal-catalyzed insertion of nitrene into γ-C­(sp^3^)–H bonds, affording oxathiazinane intermediates. However, these species are often difficult to deprotect efficiently to yield the corresponding γ-amino alcohols (Scheme 1A).^30–41^

**Scheme 1:**
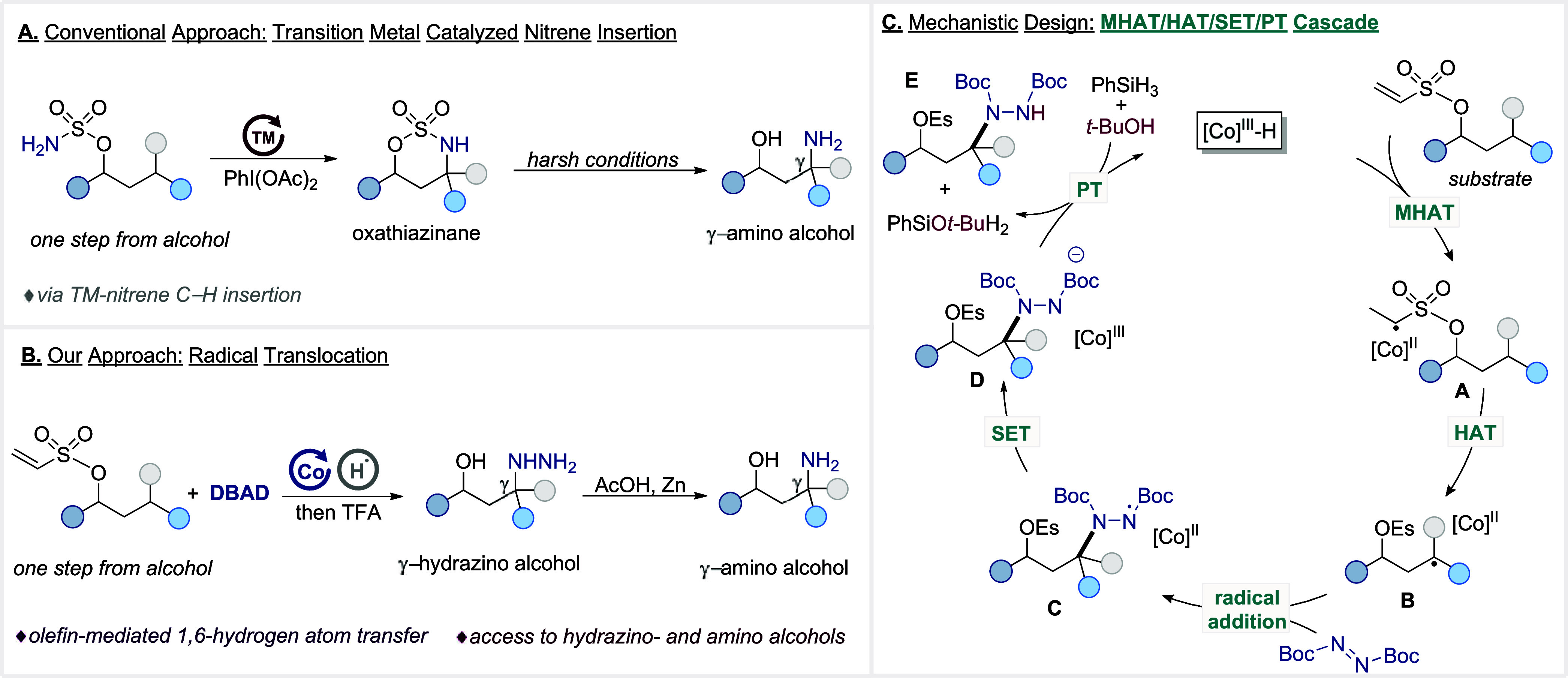
γ-Amination and Hydrazination of Alcohols

Given these limitations and guided by our interest in mild and selective radical-mediated remote C­(sp^3^)–H functionalization,^42–44^ we sought to develop an alternative, conceptually distinct strategy that merges metalhydrogen atom transfer (MHAT) with radical relay chemistry.^45^ The resulting γ-hydrazino alcohols can be readily transformed into the corresponding γ-amino alcohols via reduction with zinc in acetic acid (Scheme 1B). Our approach relies on installing a transient olefin moiety onto the alcohol substrate, which serves as a radical precursor in the presence of an *in situ*-generated [Co]^III^–H species.^46–48^ This species promotes the formation of an electrophilic alkyl radical (**A**), which undergoes an intramolecular 1,6-hydrogen atom transfer (1,6-HAT) to generate a more nucleophilic translocated radical at the γ-position (**B**).^49–51^ This polarity inversion enables a selective radical addition to di-tert-butyl azodicarboxylate (DBAD), whose strongly electron-deficient N=N bond serves as an effective electrophilic trap, affording the hydrazyl intermediate (**C**) and metalloradical [Co]^II^ complex. Subsequent single-electron transfer (SET) reduction generates intermediate (**D**) and [Co]^III^ species. A following proton transfer (PT) to (**D**) completes a two-step proton-coupled electron transfer (PCET) sequence, delivering γ-hydrazino alcohol with high site selectivity, while hydride abstraction from the silane regenerates the active cobalt catalyst (Scheme 1C).

## Results and Discussion

We initiated our studies using the 4-methylpentanol derivative (**1a**) as a model substrate for γ-hydrazination, employing DBAD as the hydrazinating reagent. Extensive reaction optimization (summarized in Table 1) identified the combination of the commercially available cobalt­(salen) complex **Co-1** and phenylsilane in a DCE:*t*-BuOH solvent mixture as the optimal conditions (Table 1, entry 1). However, instead of the expected γ-hydrazino product **2a′,** we obtained 1,3-oxazinone derivative **2a** in 97% isolated yield (Figure 1). This outcome can be rationalized by an intramolecular cyclization of the initially formed γ-hydrazino intermediate **2a′**, in which the carbamate oxygen attacks the adjacent cationic center, followed by elimination to afford the 1,3-oxazinone ring. Interestingly, this transformation–though unexpected–proved beneficial, as the oxazinone intermediate can be readily deprotected with trifluoroacetic acid (TFA) under mild conditions, providing direct access to the corresponding hydrazine. This unexpected cyclization mechanism was further supported by a control experiment using substrate **1e′**, bearing a translocated alkene moiety, which afforded the cyclized product **2e** in 60% yield, while no γ-hydrazino product **2e′** was detected. Interestingly, other tethered alcohols, such as vinyl ester **1aa** and allyl sulfonate **1ab**, failed to undergo γ-functionalization and instead preferentially underwent competitive reduction under the optimized conditions. Catalyst screening revealed that replacing **Co-1** with either **Co-2** or **Co-3** led to significantly reduced yields of 24 and 32%, respectively (Table 1, entries 2 and 3).

**Table 1:**
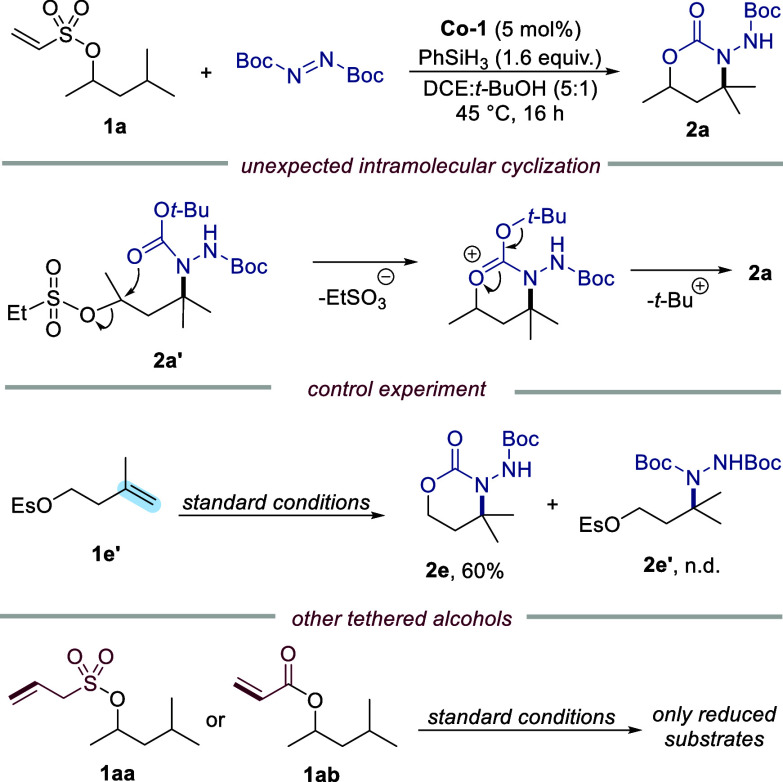

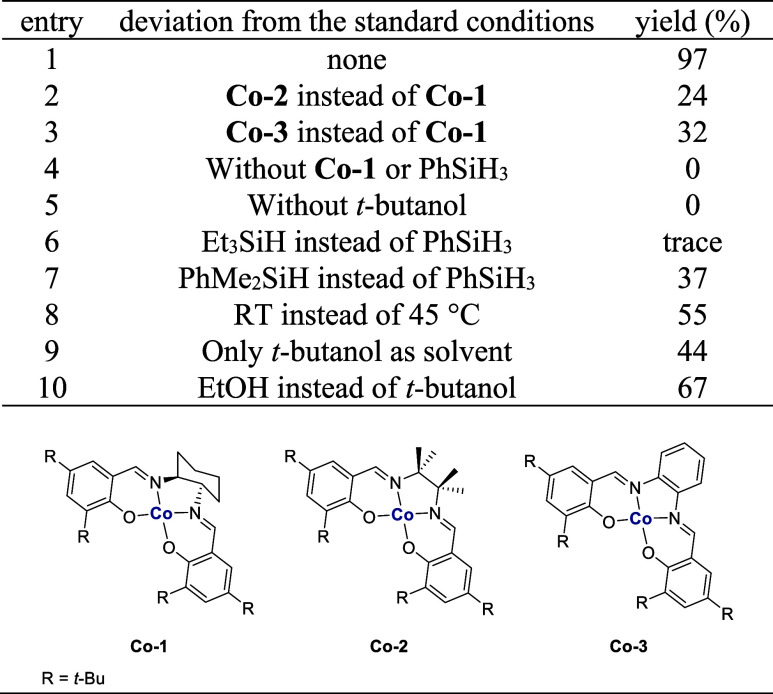
Reaction Development^a^

a Standard conditions: **1a** (0.2 mmol), **Co-1** (0.01 mmol, 6 mg), DBAD (0.6 mmol, 138 mg), PhSiH_3_ (1.6 equiv., 0.32 mmol, 40 μL), 1,2-dichloroethane (4 mL), *t-*BuOH (0.8 mL), 45 °C, 16 h, isolated yields.

**Figure 1:**
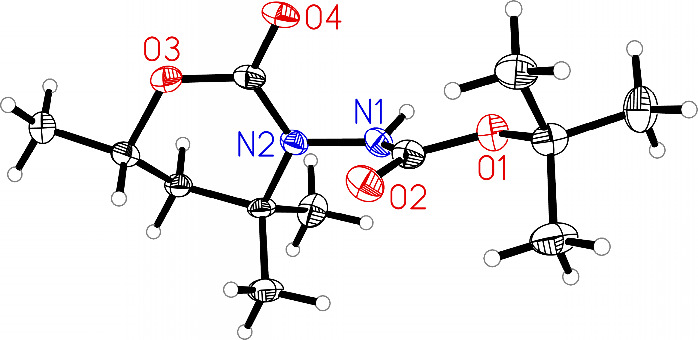
Molecular structure of **(** ***S*** **)-2a**. Displacement ellipsoids correspond to a 30% probability. Lower occupied atoms of the disordered part of the molecule have been omitted for the sake of clarity.

Control experiments confirmed the necessity of both the cobalt catalyst and the silane reductant, as the omission of either component resulted in no detectable product formation (Table 1, entry 4). Likewise, the removal of the *t*-butanol additive furnished only trace amounts of the product (Table 1, entry 5). The choice of silane proved to be critical: phenylsilane was clearly superior, while Et_3_SiH afforded only a trace yield and PhMe_2_SiH gave a diminished yield of 37% (Table 1, entries 6 and 7). Reaction temperature also influenced the outcome–conducting the reaction at room temperature decreased the yield to 34% (Table 1, entry 8). Solvent evaluation indicated that *t*-butanol alone was unsuitable (55%, Table 1, entry 9), whereas replacing *t*-butanol with ethanol in the DCE mixture provided a low yield of 67% (Table 1, entry 10).

We next explored the scope of the γ-C­(sp^3^)–H hydrazination methodology using a variety of olefin-tethered alcohols **1** with DBAD as the hydrazine source (Scheme 2). The transformation generally proceeded smoothly across a broad substrate range, affording the corresponding 1,3-oxazinone derivatives (**2a**–**2q**) in excellent yields. We first examined substrates **1a** and **1b**, which each contain a single tertiary γ-C–H bond and lack potentially competing tertiary β- or δ-C–H sites. Both underwent efficient γ-hydrazination to furnish **2a** and **2b** in excellent yields and with excellent regioselectivity. The synthetic practicality of the method was further demonstrated by a 4 mmol scale synthesis of **2a**, which afforded an isolated yield comparable to that under standard conditions. The reaction also tolerated substantial steric hindrance at the target γ-position, as demonstrated by the successful functionalization of **1c** and **1d**, which delivered the corresponding products in good yields (66–70%). In addition, primary alcohol derivatives (**1e**–**1h**) were suitable substrates, providing **2e**–**2h** in good to very good yields. A persistent challenge in remote C–H functionalization is achieving high regioselectivity when multiple C–H sites are present. To probe this aspect, we investigated substrates **1i**–**1l**, which contain both tertiary β- and γ-C–H bonds. In all cases, hydrazination occurred exclusively at the γ-position via the desired 1,6-HAT pathway, affording oxazinones **2i**–**2l** with excellent regioselectivity and yields. Likewise, substrates **1m** and **1n**, bearing both tertiary γ- and δ-C–H bonds, reacted selectively at the γ-site to furnish **2m** and **2n** in excellent yields. This outcome highlights the strong preference for a seven-membered HAT transition state. For substrate **1o**, which features both secondary and tertiary γ-C–H bonds, hydrazination occurred selectively at the tertiary site, yielding **2o** in a 97% yield. Furthermore, the applicability of the method was demonstrated with the (−)-menthol derivative, which was efficiently functionalized using isopropyl or benzyl azodicarboxylate as hydrazine precursors, affording the corresponding oxazinones **2p** and **2q** in very good yields. In contrast, substrates **1r**–**1t** were unreactive under the standard conditions, further underscoring the exclusive selectivity of the protocol for tertiary γ-C–H bonds.

**Scheme 2:**
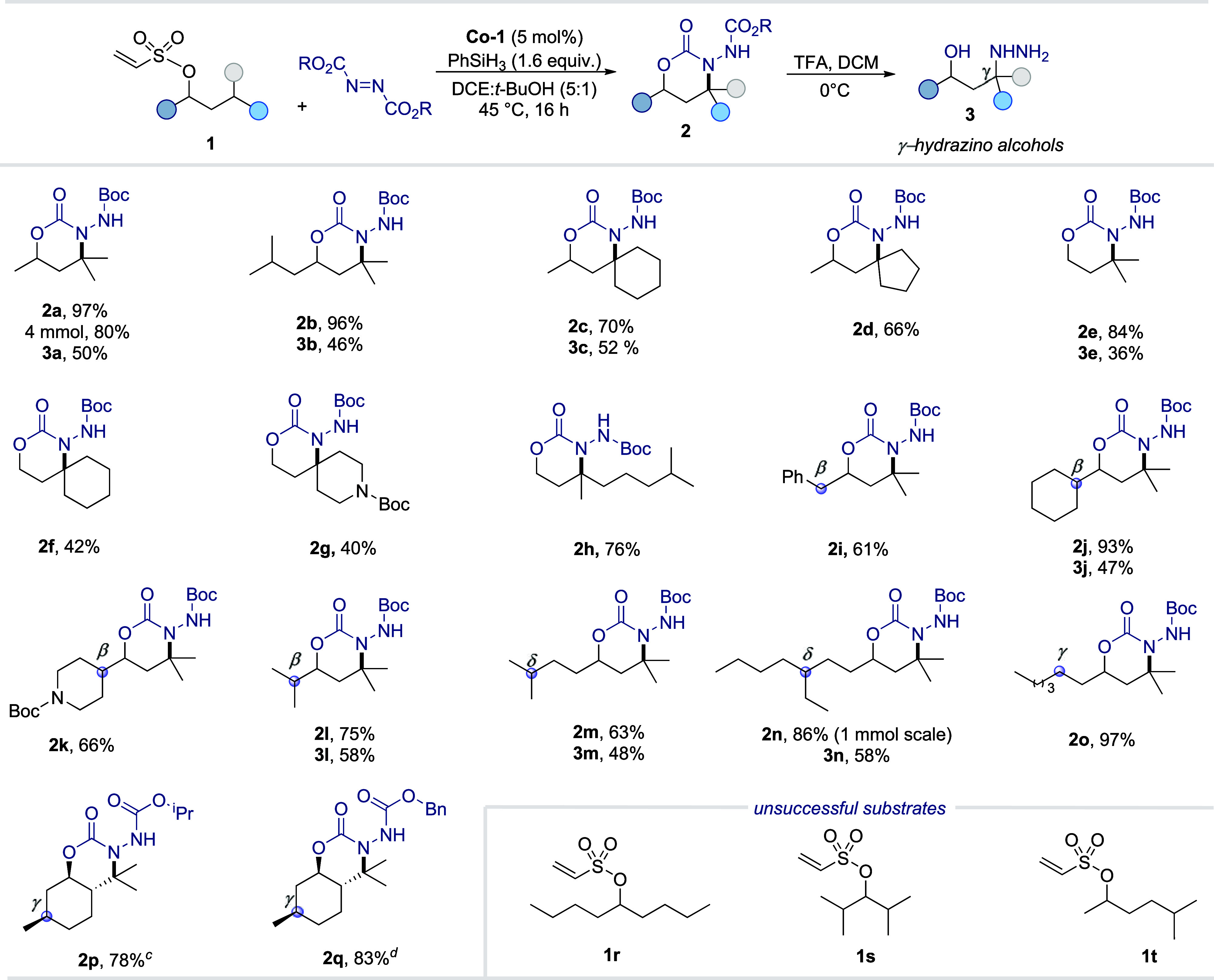
Scope of γ-C−H Hydrazination of Alcohols^a^ ^,^ ^b^

a Standard Conditions: **1** (0.2 mmol), **Co-1** (0.01 mmol, 6 mg), DBAD (0.6 mmol, 138 mg), PhSiH_3_ (1.6 equiv., 0.32 mmol, 40 μL), 1,2-Dichloroethane (4 mL), *t-*BuOH (0.8 mL), 45 °C, 16 h, Isolated Yields.
b Conditions of Deprotection of Hydrazination: **2** (0.2 mmol), TFA (10 equiv., 154 μL), DCM (2 mL), Isolated Yields ^*c*^ Diisopropyl azadicarboxylate was used. ^*d*^ Dibenzyl azadicarboxylate was used.

To demonstrate the synthetic utility of the developed methodology, we investigated the conversion of oxazinone products **2** into the corresponding γ-hydrazino alcohols **3**. Gratifyingly, treatment with TFA in DCM at 0 °C efficiently removed both protecting groups, affording the desired products in good yields. As an example of a hydrazine-to-amine transformation, compound **3n** was reduced using Zn/HOAc, followed by salt formation with Et_2_O/HCl, to furnish the corresponding γ-amino alcohol **4n** in 45% yield (see Supporting Information for details).

### Mechanistic Studies

To gain insight into the reaction mechanism, a radical trapping experiment was conducted, revealing that the addition of TEMPO completely suppressed γ-functionalization and led to detectable TEMPO–substrate adducts by HRMS, consistent with the involvement of a radical intermediate (Scheme 3).

**Scheme 3:**
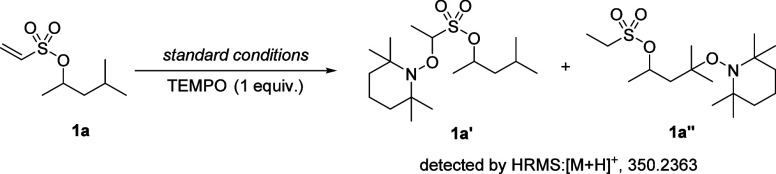
Radical Trapping Experiment with TEMPO

To investigate catalyst activation and monitor changes in the cobalt oxidation state during the interaction with the reaction components, EPR spectroscopy was employed (Figure 2a). Fresh **Co-1** in DCE:*t*-BuOH exhibited the expected pattern of a square-planar [Co]^II^ species, an EPR-active system with *g*_1_ = 1.947, *g*_2_ = 1.933, and *g*_3_ = 3.213.^52^ Upon addition of DBDA, a new isotropic signal at *g* = 2.027 appeared, corresponding to ca. 10% of the initial intensity (red line). This signal was assigned to a [Co]^II^–phenoxyl radical, generated by tautomerization between [Co^III^(phenolate)]^+^ and [Co^II^(phenoxyl•)]^+^,^53^ indicating that DBDA acts both as an oxidant and hydrazine donor. Subsequent addition of the substrate did not affect the signal (blue line), whereas introduction of phenylsilane caused its complete disappearance (green line), consistent with the formation of the EPR-silent [Co]^III^ species. Prolonged reaction times produced no detectable signal, confirming the persistence of [Co]^III^ as the resting state (Figure S1). To further probe the coordination and electronic changes of cobalt, *in situ* UV–vis spectroscopy was conducted (Figure 2b). During the first 15 min, corresponding to the activation period, [Co]^II^ was oxidized to [Co]^III^ with the coordination of an additional axial ligand. This intermediate displayed a broad LMCT band at ∼850 nm (blue curve)^54^ and a shoulder at 440 nm attributable to LMCT from a phenoxyl radical in the equatorial ligand to the cobalt center, which is consistent with EPR data.

**Figure 2:**
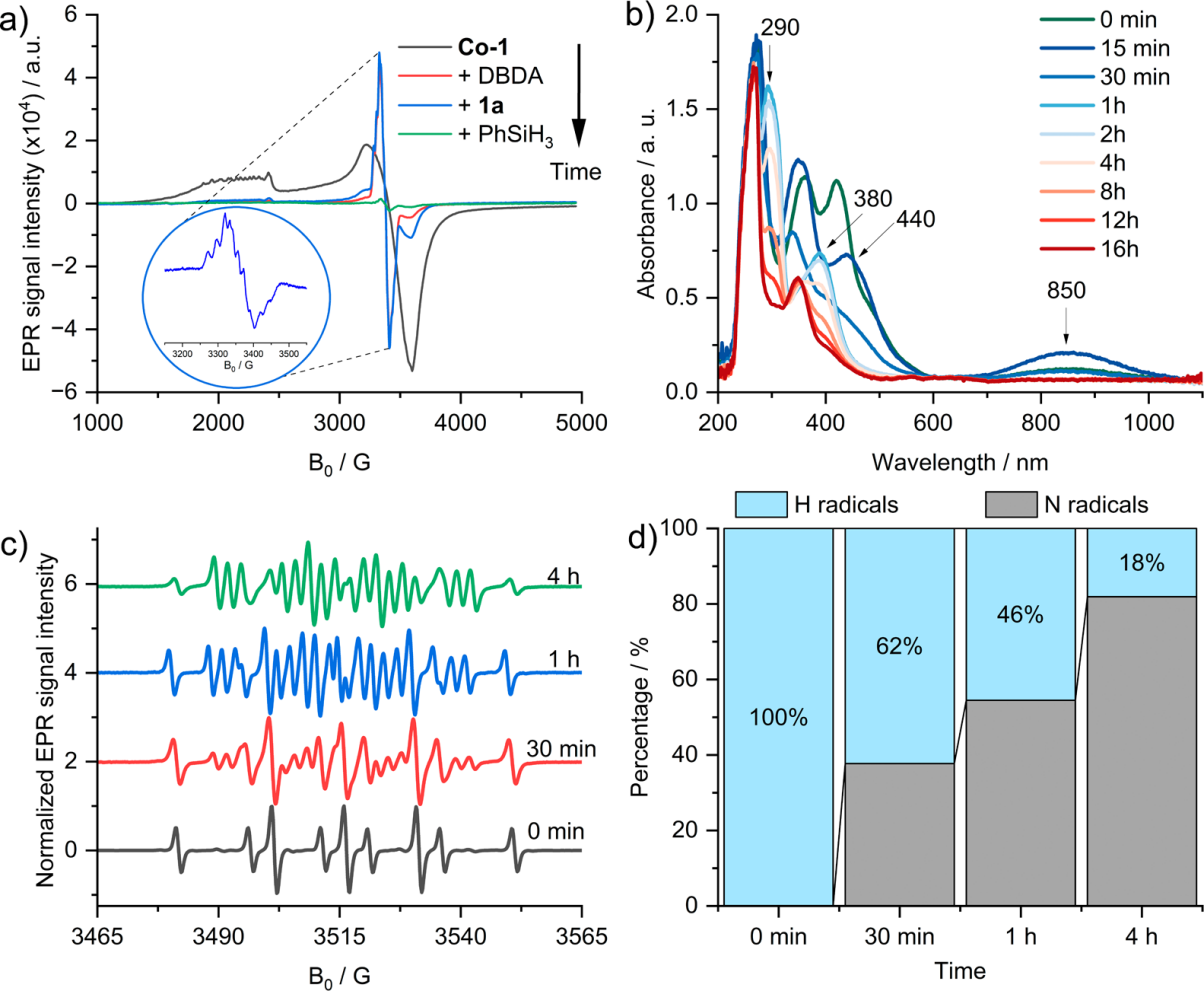
(a) EPR spectra recorded at −173 °C of **Co-1** upon addition of different components; (b) UV–vis spectra of the reaction mixture at different times; (c) EPR spectra recorded at room temperature of the reaction mixture with DMPO at different times; (d) relative amount of H radical and N radical at different times by simulating EPR spectra.

Complementary EPR spin-trapping with DMPO at the beginning period revealed only H• radicals (*a*_N_ = 14.9 G and *a*_H1_ = *a*_H2_ = 19.7 G; Figure 2c and Figure S2), confirming the formation of a [Co]^III^–H species bearing an axial hydride. After ∼60 min, the UV–vis spectrum evolved, showing new absorption bands at 380 and 290 nm (Figure 2b, light blue curve). Concurrent EPR spin-trapping experiments detected an additional *N*-centered radical (*a*_N_ = 14 G, *a*_H_ = 19.3 G, and *a*_N_ = 2.8 G) alongside the H• signal (Figure S2c), suggesting the gradual formation of a hydrazine-related intermediate, as supported by DFT calculations (*a*_N_ = 12.5 G, *a*_H_= 23.5 G, and *a*_N_ = 2.4 G). With longer reaction times, those absorbance bands diminished, indicating cobalt decomposition (Figure 2b). Notably, the ratio of N• to H• radicals increased progressively (Figure 2d), highlighting the continuous conversion of the [Co]^III^–H species into the hydrazine-forming intermediate, leading to the final product.

To elucidate the reaction pathway in greater detail, DFT calculations were carried out (Figure 3). The catalytic cycle begins with a MHAT step involving homolytic cleavage of the [Co]^III^–C bond, generating [Co]^II^ and carbon-centered radical intermediate **A1** (Figure 3a). This reactive species can follow two competing routes. In the first, radical translocation occurs via intramolecular HAT. Calculations show that 1,6-HAT is energetically favored over 1,5- and 1,7-HATs by 3.4 and 10.6 kcal mol^–1^, respectively (Figure S4). Alternatively, DBAD can couple directly with radical **A1** before HAT. The activation barrier for this addition (TS_CN1_) is 11.6 kcal mol^–1^, while the 1,6-HAT transition state (TS_HAT‑1,6_) requires only 8.6 kcal mol^–1^, confirming the kinetic preference for HAT. The resulting translocated radical **B1** then couples with the DBDA through TS_CN2_ (Δ*G* ^‡^ = 13.9 kcal mol^–1^) to afford complex **C1**. The process proceeds through an outer-sphere single-electron transfer (SET) step (Figure 3b), in which [Co]^II^ donates an electron to **C1** without forming a bridging ligand. Two redox pathways were examined. Reduction of [Co]^II^ to [Co]^I^ is highly endergonic (*E*_red_ = −2.48 V vs NHE), whereas oxidation to [Co]^III^, affording the aminoanionic complex **D1**, is more accessible (*E*_ox_ = −0.72 V vs NHE), indicating that oxidation is thermodynamically preferred.

**Figure 3:**
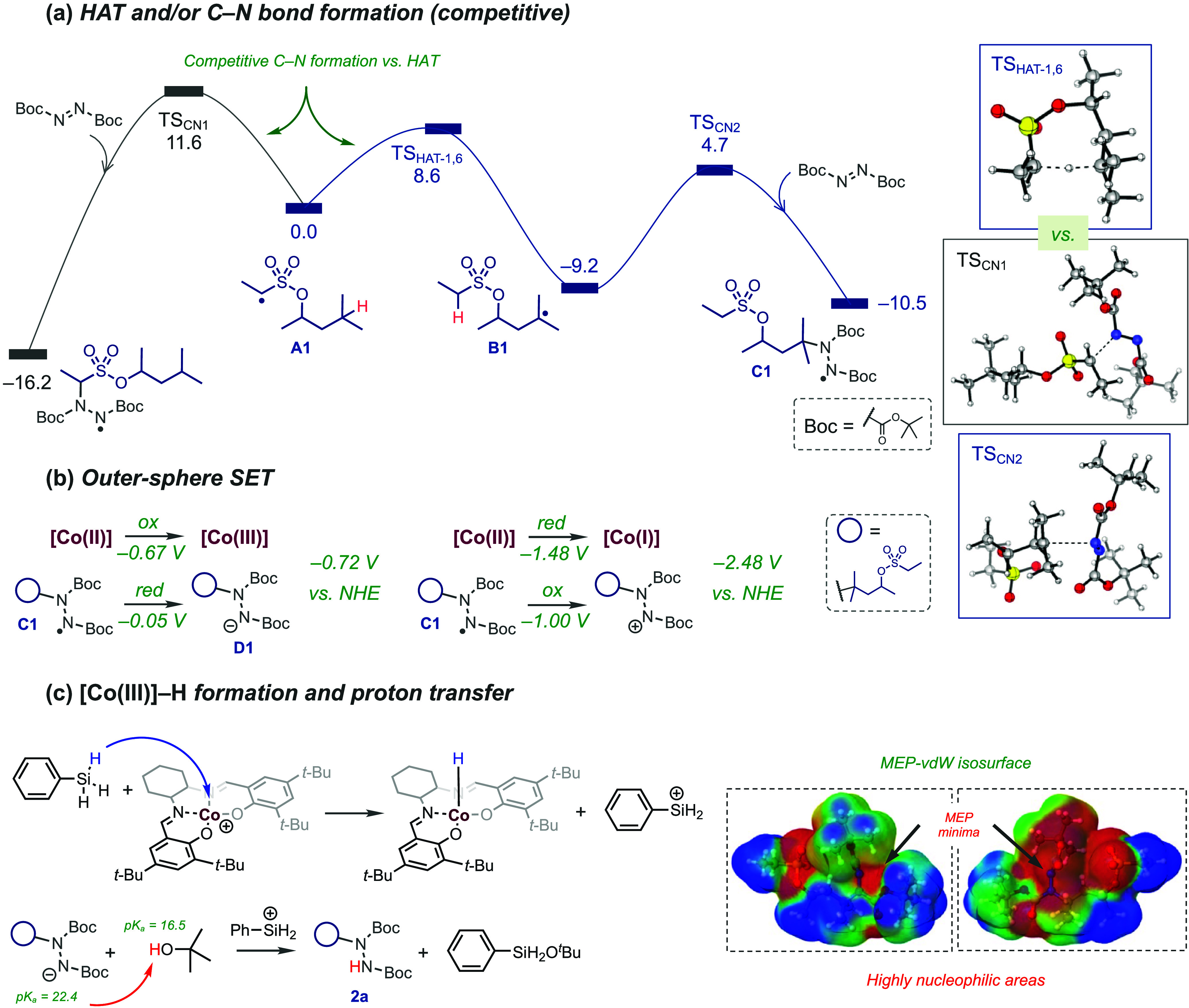
Computational study. (a) Potential energy surface (PES) comprising HAT and radical addition to DBAD. (b) Plausible outer-sphere SET processes involving [Co]^II^ oxidation versus reduction. (c) Formation of the [Co]^III^–H intermediate via an acid–base reaction and product formation; MEP-vdW refers to molecular electrostatic potential (MEP) at the van der Waals (vdW) isosurface. Free energies (Δ*G*, kcal mol^–1^) have been calculated at the BPW91/TZVP­(dichloromethane)//BPW91/SVP­(vacuum) level of theory at 45 °C, while redox potentials have been computed under standard conditions.

Finally, the protonation of complex **D1** by *t*-BuOH yields the aminated product **2a** (Figure 3c). The p*K*_a_ of *t*-BuOH in dichloromethane (16.5) is lower than that of the conjugate acid of **D1** (22.4), showing that *t*-BuOH acts as a proton donor. Molecular electrostatic potential (MEP) analysis of **D1** reveals a region of high negative potential near the azodicarboxylate nitrogen, supporting the feasibility of proton transfer and confirming the proposed acid–base step that produces the hydrazinated product.

## Conclusions

In conclusion, we have developed a selective γ-C­(sp^3^)–H hydrazination of alcohols through cobalt­(salen)-catalyzed MHAT and radical translocation. The reaction proceeds under mild and sustainable conditions, tolerating diverse substrates to afford γ-hydrazino alcohols that are readily convertible into γ-amino alcohols. Combined spectroscopic and computational studies support a rare MHAT/HAT/SET/PT cascade, revealing an unusual convergence of radical HAT^55^ and polar PCET^56^ processes within a single catalytic cycle. This cobalt-based approach highlights the capability of base-metal catalysis to promote radical transformations,^57,58^ paving the way for the development of mechanistically distinct and sustainable pathways in C–H functionalization chemistry.^59^

## Experimental Content

All experimental details in this paper, such as synthetic procedure, characterization data, computational information, and NMR spectral data, are included in the Supporting Information.

## Supplementary Material



## Notes

Experimental data, Characterization data for all new compounds, copies of NMR spectra and computational information (PDF)

## #

State Key laboratory of Low Carbon Catalysis and Carbon Dioxide Utilization, Lanzhou Institute of Chemical Physics, Chinese Academy of Sciences, No. 18, Tianshui Middle Road, Lanzhou 730000, China (J.R.)
