# Supplementary material for: Merging Double Hydrogen Atom Transfer and Stepwise Proton-Coupled Electron Transfer for γ‑C–H Hydrazination of Alcohols
Source: JACS Au. 2026 Jan 26;6(2):1012–9. doi: 10.1021/jacsau.5c01435 (PMC12933358; doi:10.1021/jacsau.5c01435)
Supplement: Supplementary file 1 [file au5c01435_si_001.pdf]

Supporting Information

Merging Double Hydrogen Atom Transfer and stepwise Proton-Coupled Electron Transfer for γ-C–H Hydrazination of Alcohols

Kaiming Zuo,[†] Phong Dam,[†] Kosala N. Amarasinghe,[†] Anke Spannenberg,[†] Jabor Rabeah,[†⸸] Olga S. Bokareva,[†§] Luis Miguel Azofra,[‡] and Osama El-Sepelgy[†]*

[†]Leibniz Institute for Catalysis e.V., Albert-Einstein-Str. 29a, 18059 Rostock, Germany

[‡]Instituto de Estudios Ambientales y Recursos Naturales (i-UNAT), Universidad de Las Palmas de Gran Canaria (ULPGC), Campus de Tafira, 35017 Las Palmas de Gran Canaria, Spain

[§]Institute of Chemistry and Department of Life, Light & Matter, University of Rostock, Albert-Einstein-Str. 25 and 27, 18059 Rostock, Germany

[⸸]State Key laboratory of Low Carbon Catalysis and Carbon Dioxide Utilization, Lanzhou Institute of Chemical Physics, Chinese Academy of Sciences, No.18, Tianshui Middle Road, Lanzhou, 730000, China

Email: Osama.Elsepelgy@Catalysis.de

Table of Contents

1. General Information **3**

2. General procedures **4**

2.1. General procedure for hydrazination of aliphatic alcohols *4*

2.2. Deprotection of 1,3-dioxazinone derivative[1] *4*

2.3. Hydrazine-to-amine transformation[2] *5*

3. Optimization conditions **6**

3.1. Optimization of catalyst loading *6*

3.2. Optimization of silane loading *6*

3.3. Optimization of co-solvent with t-butanol *7*

3.4. Optimization of temperature *7*

4. Additive robustness study **8**

5. Characterization data **9**

6. Mechanistic studies-EPR spectroscopy **18**

7. DFT calculations **20**

7.1. Computational Details *20*

7.2. Optimized Cartesian coordinates *24*

8. Single crystal structure **32**

9. References **33**

10. NMR spectra **34**

1. General Information

All commercial reagents were purchased from commercial suppliers and used without further purification and all solvents were treated according to the general methods. The reactions were monitored by thin layer chromatography (TLC) with aluminum sheets silica gel 60 F_{254} from Merck, and flash column chromatography purifications were performed using silica gel 60 (63-200 μm) from MACHEREY-NAGEL. ^{1}H and ^{13}C NMR spectra were recorded with Bruker AV 300 (300 MHz), AV 400 (400 MHz) or Fourier 300 (300 MHz) NMR spectrometers. Chemical shifts (*δ*) are given relative to solvent: references for $CDCl_3$ were 7.26 ppm (^{1}H NMR) and 77.16 ppm (^{13}C NMR). And all signals were reported in parts per million (ppm) and spin-spin coupling constants (*J*) are given in Hz, while multiplicities are abbreviated by s (singlet), d (doublet), t (triplet), q (quartet), br (broad), m (multiplet). And all HRMS data were collected by the Waters Acquity UPLC H-Class/Xevo G2-XS TOF LC-MS (ion trap). All measurements were carried out at room temperature unless otherwise stated.

2. General procedures

2.1. General procedure for hydrazination of aliphatic alcohols

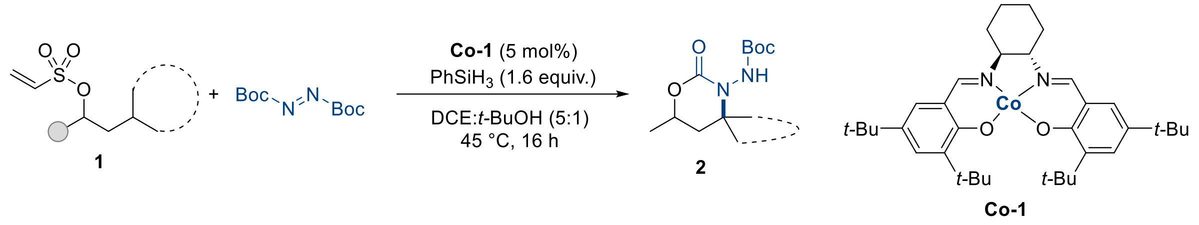

In an oven-dried 25 mL Schlenk tube equipped with a magnetic stir bar, olefin-tethered alcohols **1** (0.20 mmol), **Co-1** (5 mol%, 6 mg), di-tert-butyl azodicarboxylate (0.60 mmol, 138 mg), 1,2-dichloroethane (4.0 mL), and *tert*-butanol (0.80 mL) were added. The reaction mixture was placed under an argon atmosphere, and phenylsilane (1.6 equiv., 3.2 mmol, 40 μL) was subsequently added. The resulting mixture was stirred at 45 °C for 16 h.

After completion, the reaction mixture was filtered through a short silica gel plug and washed with dichloromethane/methanol (98:2, v/v). The solvent was removed under reduced pressure, and the crude residue was purified by column chromatography on silica gel using hexane/ethyl acetate (2:1, v/v) as the eluent to afford the pure 1,3-oxazinone derivative **2**.

2.2. Deprotection of 1,3-dioxazinone derivatives[1]

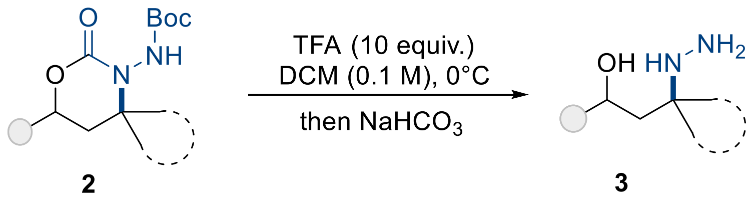

To a solution of compound **2** (0.20 mmol) in dichloromethane (2.0 mL) at 0 °C was added trifluoroacetic acid (0.15 mL). The resulting mixture was stirred at this temperature for 3 h. After completion, acetone (3.0 mL) was added, and the reaction mixture was stirred for an additional 10 min. The reaction was then neutralized by the careful addition of solid sodium bicarbonate, and the mixture was filtered through anhydrous sodium sulfate. The filtrate was extracted with dichloromethane, and the combined organic layers were concentrated under reduced pressure. The crude product was purified by flash column chromatography on silica gel using ethyl acetate as the eluent.

2.3. Hydrazine-to-amine transformation[2]

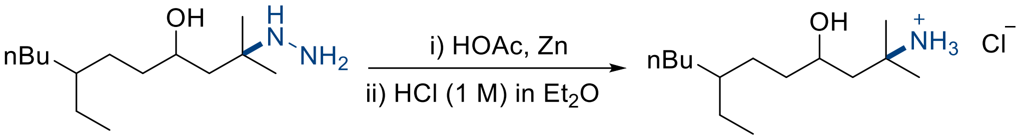

3n **4n**

To a solution of compound **3n** (0.20 mmol) in acetic acid (2.0 mL) was added freshly activated zinc powder (200 mg). After purging the reaction mixture with argon, the suspension was stirred at room temperature overnight. The reaction mixture was then filtered, and the filtrate was concentrated under reduced pressure to afford a residue. The residue was diluted with dichloromethane and basified by the addition of a saturated aqueous solution of sodium carbonate. The resulting mixture was extracted with dichloromethane (3 ×), and the combined organic layers were dried over anhydrous sodium sulfate. After filtration and removal of the solvent under reduced pressure, the crude material was purified by flash column chromatography on silica gel using hexane/ethyl acetate (1:1, v/v) as the eluent.

Salt formation: The purified free base was dissolved in diethyl ether and treated with hydrochloric acid (3.0 equiv). Excess hexane was added to induce precipitation, and the resulting suspension was stored at 4 °C for 4 h. The precipitated hydrochloride salt was collected by filtration to afford compound **4n·** as a white solid (20 mg, 45% yield).

3. Optimization conditions

3.1. Optimization of catalyst loading

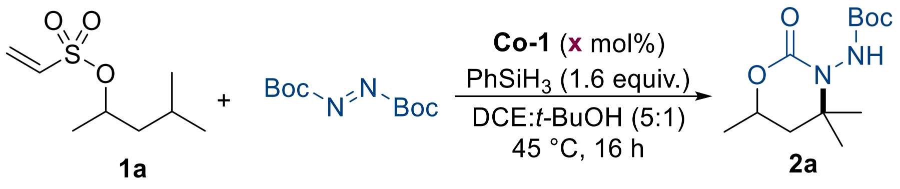

Entry	Amount of Co-1 (mol %)	Yield (%)
1	2.5	21
2	5	97
3	7.5	89
4	10	63

3.2. Optimization of silane loading

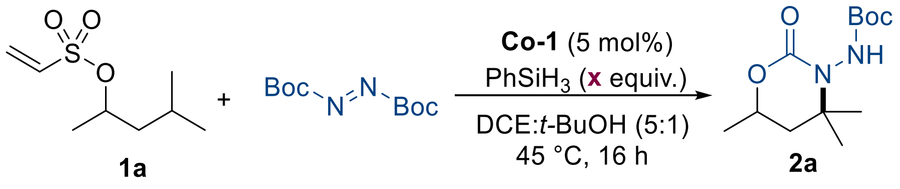

Entry	Amount of $PhSiH_3$ (equiv. to 1a)	Yield (%)
1	1	68
2	1.6	97
3	2	79

3.3. Optimization of co-solvent with t-butanol

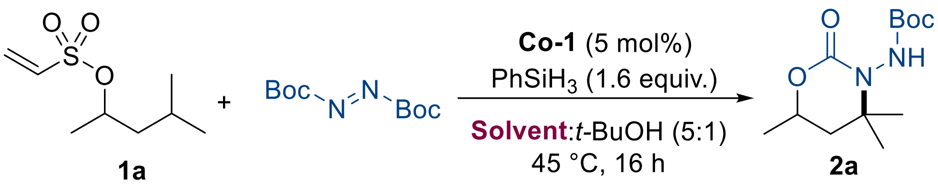

Entry	Solvent	Yield (%)
1	DCE	97
2	DCM	43
3	Acetone	40
4	MeCN	25
5	Dioxane	38

3.4. Optimization of temperature

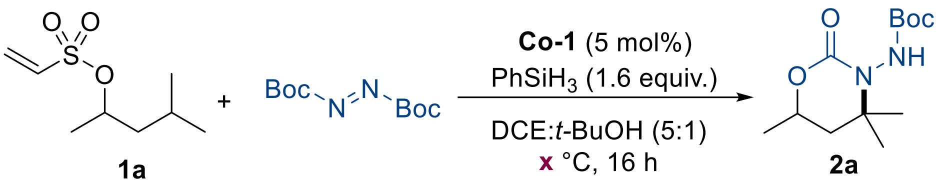

Entry	Temperature (°C)	Yield (%)
1	RT	55
2	45	97
3	60	31
4	90	15
6	Blue LEDs at RT	48
7	Blue LEDs at 45 °C	65

4. Additive robustness study

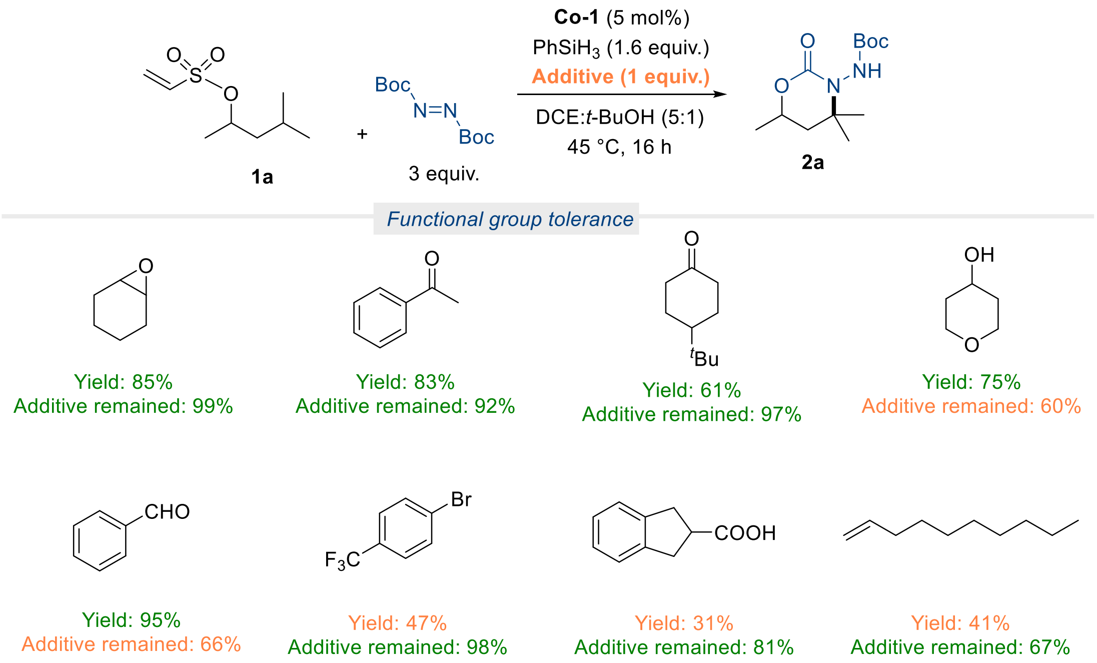

5. Characterization data

tert-butyl (4,4,6-trimethyl-2-oxo-1,3-oxazinan-3-yl)carbamate (2a)

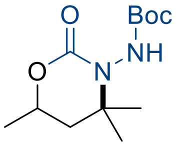

Purification by chromatography (hexane: EA = 2:1); Yield: 97% (light yellow solid, 50 mg).

^{1}H NMR (300 MHz, $CDCl_3$) δ = 6.48 – 6.36 (br, 1H), 4.49 (br, 1H), 1.95 – 1.77 (m, 2H), 1.44 (s, 9H), 1.37 – 1.24 (m, 9H).

^{13}C NMR (75 MHz, $CDCl_3$) δ ppm 156.3, 81.6, 69.9, 58.5, 44.1, 28.2, 26.3, 20.9, 20.6.

HRMS (ESI-TOF) m/z: $[M + Na]^+$ Calcd for $C_{12}H_{22}N_2O_4Na$ 281.1472; Found 281.1482.

tert-butyl (6-isobutyl-4,4-dimethyl-2-oxo-1,3-oxazinan-3-yl)carbamate (2b)

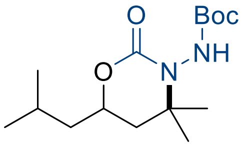

Purification by chromatography (hexane: EA = 2:1); Yield: 96% (light yellow solid, 58 mg).

^{1}H NMR (300 MHz, $CDCl_3$) δ = 6.52 – 6.41 (br, 1H), 4.39 (br, 1H), 1.91 – 1.78 (m, 2H), 1.66 – 1.57 (m, 1H), 1.44 (s, 9H), 1.36 – 1.18 (m, 8H), 0.91 (d, *J*=5.1, 3H), 0.88 (d, *J*=5.1, 3H).

^{13}C NMR (75 MHz, $CDCl_3$) δ ppm 156.3, 81.5, 71.8, 58.5, 43.7, 42.9, 29.7, 28.2, 26.3, 24.1, 23.0, 22.3.

HRMS (ESI-TOF) m/z: $[M + Na]^+$ Calcd for $C_{15}H_{28}O_4N_2Na$ 323.1942; Found 323.1949.

tert-butyl (4-methyl-2-oxo-3-oxa-1-azaspiro[5.5]undecan-1-yl)carbamate (2c)

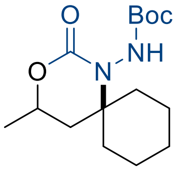

Purification by chromatography (hexane: EA = 2:1), Yield: 70% (light yellow solid, 42 mg).

^{1}H NMR (300 MHz, $CDCl_3$) δ = 6.27 – 6.12 (br, 1H), 4.4 (br, 1H), 2.34 (dd, *J*=14.2, 1.8, 1H), 1.98 – 1.89 (m, 1H), 1.82 – 1.66 (m, 7H), 1.47 (s, 9H), 1.36 (d, *J*=6.2, 3H), 1.29 – 1.23 (m, 3H).

^{13}C NMR (75 MHz, $CDCl_3$) δ ppm 156.6, 81.9, 69.8, 61.7, 38.6, 37.0, 32.4, 28.3, 25.0, 22.7, 21.2.

HRMS (ESI-TOF) m/z: $[M + Na]^+$ Calcd for $C_{15}H_{26}O_4N_2Na$ 321.1785; Found 321.1794.

tert-butyl (9-methyl-7-oxo-8-oxa-6-azaspiro[4.5]decan-6-yl)carbamate (2d)

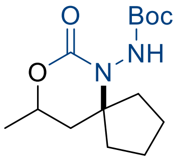

Purification by chromatography (hexane: EA = 2:1), Yield: 66% (Light yellow solid, 27 mg).

^{1}H NMR (300 MHz, CDCl$_3$) δ = 6.31 (br, 1H), 4.42 (br, 1H), 2.12 – 1.87 (m, 3H), 1.80 – 1.52 (m, 7H), 1.47 (s, 9H), 1.35 (d, *J*=6.3, 3H).

^{13}C NMR (75 MHz, CDCl$_3$) δ ppm 156.9, 82.4, 71.2, 69.2, 43.5, 37.4, 36.0, 28.6, 24.2, 23.7, 21.4, 21.1.

HRMS (ESI-TOF) m/z: $[M + Na]^+$ Calcd for $C_{14}H_{24}O_4N_2Na$ 307.1629; Found 307.1639.

tert-butyl (4,4-dimethyl-2-oxo-1,3-oxazinan-3-yl)carbamate (2e)

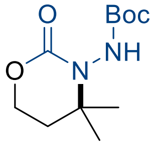

Purification by chromatography (hexane: EA = 2:1); Yield: 84% (white solid, 41 mg).

^{1}H NMR (300 MHz, CDCl$_3$) δ = 6.25 (br, 1H), 4.31 (br, 1H), 4.24 (s, 1H), 2.05 – 2.01 (m, 2H), 1.48 (s, 9H), 1.33 (d, *J*=10.8, 6H).

^{13}C NMR (75 MHz, CDCl$_3$) δ ppm 156.2, 81.8, 62.9, 58.9, 36.5, 28.1, 27.1, 26.2.

HRMS (ESI-TOF) m/z: $[M + Na]^+$ Calcd for $C_{11}H_{20}O_4N_2Na$ 267.1321; Found 267.132.

tert-butyl (2-oxo-3-oxa-1-azaspiro[5.5]undecan-1-yl)carbamate (2f)

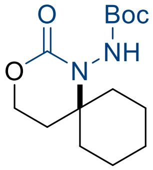

Purification by chromatography (hexane: EA = 1:1); Yield: 42% (light yellow solid, 24 mg).

^{1}H NMR (300 MHz, CDCl$_3$) δ = 6.21 (br, 1H), 4.35 – 4.14 (m, 2H), 2.24 (d, *J*=13.8, 1H), 2.02 – 1.56 (m, 7H), 1.48 (s, 9H), 1.40 – 1.07 (m, 4H).

^{13}C NMR (75 MHz, CDCl$_3$) δ ppm 156.5, 81.9, 62.8, 62.2, 35.4, 33.0, 30.8, 28.3, 25.0, 22.8, 22.7.

HRMS (ESI-TOF) m/z: $[M + Na]^+$ Calcd for $C_{14}H_{24}O_4N_2Na$ 307.1629; Found 307.1635.

tert-butyl 1-((tert-butoxycarbonyl)amino)-2-oxo-3-oxa-1,9-diazaspiro[5.5]undecane-9-carboxylate(2g)

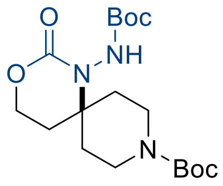

Purification by chromatography (hexane: EA = 2:1); Yield: 40% (light yellow solid, 31 mg).

^{1}H NMR (300 MHz, CDCl3) δ = 6.25 (br, 1H), 4.32 – 4.25(m, 2H), 4.11– 4.07 (m, 2H), 2.77 (m, 2H), 2.26 (m, 1H), 2.17 – 1.85 (m, 3H), 1.84 – 1.61 (m, 2H), 1.46 (dm, 18H).

^{13}C NMR (75 MHz, CDCl3) δ ppm 156.5, 154.7, 154.3, 82.3, 80.2, 62.4, 60.5, 40.2, 34.6, 30.0, 28.5, 28.2.

HRMS (ESI-TOF) m/z: $[M + Na]^+$ Calcd for $C_{18}H_{31}O_6N_3Na$ 408.2105; Found 408.2111.

tert-butyl (S)-(4-methyl-4-(4-methylpentyl)-2-oxo-1,3-oxazinan-3-yl)carbamate (2h)

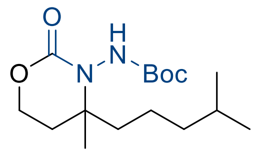

Purification by chromatography (hexane: EA = 1:1); Yield: 76% (light yellow solid, 48 mg).

^{1}H NMR (300 MHz, CDCl3) δ = 6.16 (br, 1H), 4.40 – 4.22 (m, 2H), 2.19 – 2.10 (m, 1H), 1.87 – 1.83 (m, 1H), 1.69 – 1.55 (m, 3H), 1.48 (s, 9H), 1.31 – 1.13 (m, 7H), 0.87 (d, *J*=6.6, 6H).

^{13}C NMR (75 MHz, CDCl3) δ ppm 156.2, 81.8, 62.8, 61.6, 39.4, 39.1, 33.2, 28.2, 28.1, 27.9, 24.7, 22.6, 21.6.

HRMS (ESI-TOF) m/z: $[M + Na]^+$ Calcd for $C_{16}H_{30}O_4N_2Na$ 337.2098; Found 337.2106.

tert-butyl (6-benzyl-4,4-dimethyl-2-oxo-1,3-oxazinan-3-yl)carbamate(2i)

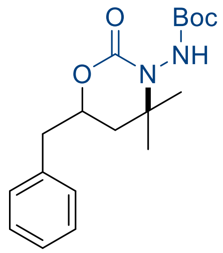

Purification by chromatography (hexane: EA = 2:1); Yield: 61% (light yellow solid, 41 mg).

^{1}H NMR (300 MHz, CDCl3) δ = 7.33 – 7.16 (m, 5H), 6.42 (br, 1H), 4.58 (br, 1H), 3.13 – 3.07 (m, 1H), 2.84 – 2.77 (m, 1H), 1.94 – 1.68 (m, 2H), 1.46 (s, 9H), 1.25 – 1.23 (m, 6H).

^{13}C NMR (75 MHz, CDCl3) δ ppm 156.2, 136.0, 129.6, 128.7, 127.0, 81.7, 74.0, 58.5, 40.9, 28.3, 28.2, 26.2.

HRMS (ESI-TOF) m/z: $[M + Na]^+$ Calcd for $C_{18}H_{26}O_4N_2Na$ 357.1785; Found 357.1794.

tert-butyl (6-cyclohexyl-4,4-dimethyl-2-oxo-1,3-oxazinan-3-yl)carbamate (2j)

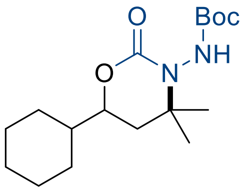

Purification by chromatography (hexane: EA = 2:1); Yield: 93% (light yellow oil, 61 mg).

^{1}H NMR (300 MHz, $CDCl_3$) δ = 6.36 – 6.25 (br, 1H), 4.11 (br, 1H), 1.93 – 1.65 (m, 8H), 1.46 (s, 9H), 1.28 (s, 6H), 1.21 – 1.01 (m, 5H).

^{13}C NMR (75 MHz, $CDCl_3$) δ ppm 156.3, 81.7, 77.4, 58.4, 41.4, 39.5, 28.3, 28.2, 28.2, 26.3, 26.0, 25.9.

HRMS (ESI-TOF) m/z: $[M + Na]^+$ Calcd for $C_{17}H_{30}O_4N_2Na$ 349.2098; Found 349.2109.

tert-butyl 4-(3-((tert-butoxycarbonyl)amino)-4,4-dimethyl-2-oxo-1,3-oxazinan-6-yl)piperidine-1-carboxylate (2k)

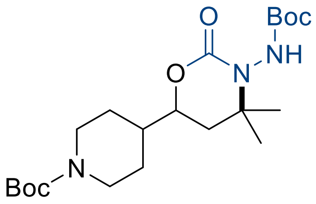

Purification by chromatography (hexane: EA = 1:1); Yield: 66% (light yellow solid, 57 mg).

^{1}H NMR (300 MHz, $CDCl_3$) δ = 6.32 (br, 1H), 4.17 – 4.12 (m, 3H), 2.73 – 2.56 (m, 2H), 1.95 – 1.74 (m, 3H), 1.74 – 1.56 (m, 2H), 1.46 (s, 9H), 1.43 (s, 9H), 1.34 – 1.21 (m, 8H).

^{13}C NMR (75 MHz, $CDCl_3$) δ ppm 156.2, 154.8, 81.8, 79.6, 58.4, 43.5, 40.0, 28.5, 28.2, 27.5, 27.3, 26.2.

HRMS (ESI-TOF) m/z: $[M + Na]^+$ Calcd for $C_{21}H_{37}O_6N_3Na$ 450.2575; Found 450.2569.

tert-butyl (6-isopropyl-4,4-dimethyl-2-oxo-1,3-oxazinan-3-yl)carbamate (2l)

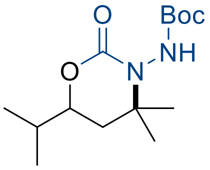

Purification by chromatography (hexane: EA = 2:1); Yield: 75% (light yellow solid, 43 mg).

^{1}H NMR (300 MHz, $CDCl_3$) δ = 6.44 – 6.30 (br, 1H), 4.09 (br, 1H), 1.90 – 1.81 (m, 3H), 1.46 (s, 9H), 1.28 (s, 6H), 0.98 (d, *J*=6.8, 3H), 0.94 (d, *J*=6.9, 3H).

^{13}C NMR (75 MHz, $CDCl_3$) δ ppm 156.3, 81.7, 78.0, 58.4, 39.4, 31.7, 28.2, 26.2, 17.9, 17.7.

HRMS (ESI-TOF) m/z: $[M + Na]^+$ Calcd for $C_{14}H_{26}O_4N_2Na$ 309.1785; Found 309.1791.

tert-butyl (6-isopentyl-4,4-dimethyl-2-oxo-1,3-oxazinan-3-yl)carbamate (2m)

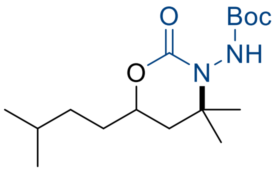

Purification by chromatography (hexane: EA = 2:1); Yield: 63% (light yellow solid, 40 mg).

^{1}H NMR (300 MHz, CDCl$_3$) δ = 6.28 (br, 1H), 4.33 (br, 1H), 1.96 – 1.75 (m, 2H), 1.68 – 1.66 (m, 1H), 1.59 – 1.51 (s, 2H), 1.48 (s, 9H), 1.31 – 1.29 (m, 8H), 0.89 – 0.88 (m, 6H).

^{13}C NMR (75 MHz, CDCl$_3$) δ ppm 156.3, 81.9, 77.4, 73.9, 42.6, 33.9, 32.6, 28.2, 28.0, 26.4, 22.6.

HRMS (ESI-TOF) m/z: [M + Na]$^+$ Calcd for $C_{16}H_{30}O_4N_2Na$ 337.2098; Found 337.2096.

ert-butyl (6-(3-ethylheptyl)-4,4-dimethyl-2-oxo-1,3-oxazinan-3-yl)carbamate (2n)

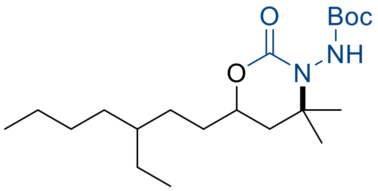

Purification by chromatography (hexane: EA = 1:1); Yield: 86% (light yellow solid, 64 mg).

^{1}H NMR (300 MHz, CDCl$_3$) δ = 6.46 (br, 1H), 4.30 (br, 1H), 1.97 – 1.77 (m, 2H), 1.64 (s, 1H), 1.45 (s, 9H), 1.44 (s, 6H), 1.29 – 1.19 (m, 12H), 0.93 – 0.73 (m, 6H).

^{13}C NMR (75 MHz, CDCl$_3$) δ ppm 156.3, 155.9, 81.7, 74.0, 58.5, 42.5, 38.7, 32.7, 31.9, 28.9, 28.2, 25.8, 23.2, 14.2, 10.9.

HRMS (ESI-TOF) m/z: [M + Na]$^+$ Calcd for $C_{20}H_{38}O_4N_2Na$ 393.2724; Found 393.2731.

tert-butyl (6-hexyl-4,4-dimethyl-2-oxo-1,3-oxazinan-3-yl)carbamate (2o)

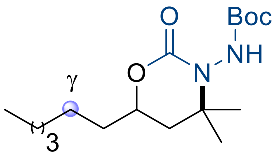

Purification by chromatography (hexane: EA = 2:1), Yield: 97% (Light yellow solid, 64 mg).

^{1}H NMR (300 MHz, CDCl$_3$) δ = 6.50 (br, 1H), 4.31 (br, 1H), 1.91 – 1.77 (m, 2H), 1.74 – 1.57 (m, 1H), 1.55 – 1.48 (m, 1H), 1.44 (s, 9H), 1.28 – 1.22 (m, 14H), 0.91 – 0.79 (m, 3H).

^{13}C NMR (75 MHz, CDCl$_3$) δ ppm 156.3, 81.6, 73.5, 58.5, 42.5, 34.7, 31.7, 29.1, 28.2, 26.2, 24.8, 22.6, 14.1.

HRMS (ESI-TOF) m/z: [M + Na]$^+$ Calcd for $C_{17}H_{32}N_2Na$ 351.2255; Found 351.2266.

isopropyl ((4aS,7R,8aR)-4,4,7-trimethyl-2-oxohexahydro-2H-benzo[e][1,3]oxazin-3(4H)-yl)carbamate (2p)

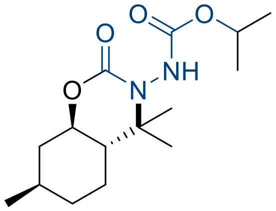

Purification by chromatography (hexane: EA = 2:1); Yield: 83% (Light yellow solid, 50 mg).

^{1}H NMR (300 MHz, CDCl$_3$) δ = 6.48 (br, 1H), 5.03 – 4.83 (m, 1H), 4.73 (br, 1H), 2.11 – 2.06 (m, 1H), 1.87 – 1.72 (m, 3H), 1.70 – 1.45 (m, 2H), 1.36 – 1.18 (m, 13H), 1.12 – 0.95 (m, 1H), 0.87 (d, *J*=6.4, 3H).

^{13}C NMR (75 MHz, CDCl$_3$) δ ppm 156.9, 72.8, 72.4, 70.1, 61.3, 43.8, 43.0, 38.9, 38.6, 33.8, 33.6, 28.1, 27.8, 25.6, 25.4, 24.1, 23.5, 22.0, 21.8.

HRMS (ESI-TOF) m/z: $[M + Na]^+$ Calcd for $C_{15}H_{26}O_4N_2Na$ 321.1785; Found 321.1788.

benzyl ((4aS,7R,8aR)-4,4,7-trimethyl-2-oxohexahydro-2H-benzo[e][1,3]oxazin-3(4H)-yl)carbamate (2q)

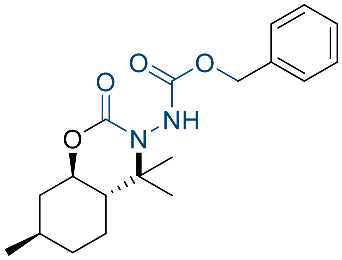

Purification by chromatography (hexane: EA = 2:1); Yield: 78% (yellow solid, 54 mg).

^{1}H NMR (400 MHz, CDCl$_3$) δ = 7.40 – 7.27 (m, 5H), 6.75 (br, 1H), 5.20 – 4.11 (m, 2H), 4.76 (br, 1H), 2.11 – 2.09 (m, 1H), 1.86 – 1.66 (m, 3H), 1.66 – 1.40 (m, 2H), 1.40 – 1.18 (m, 6H), 1.17 – 0.92 (m, 2H), 0.88 (d, *J*=6.5, 3H).

^{13}C NMR (101 MHz, CDCl$_3$) δ ppm 157.2, 154.2, 135.8, 128.7, 128.2, 72.6, 67.9, 61.4, 43.7, 43.0, 38.6, 33.6, 27.7, 24.1, 23.6, 21.8, 14.3.

HRMS (ESI-TOF) m/z: $[M + Na]^+$ Calcd for $C_{19}H_{26}O_4N_2Na$ 369.1785; Found 369.1783.

4-hydrazineyl-4-methylpentan-2-ol (3a)

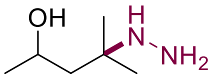

Purification by chromatography (EA); Yield: 50% (colorless oil, 20 mg).

^{1}H NMR (300 MHz, CDCl$_3$) δ = 9.02 (s, 1H), 4.72 – 4.53 (m, 1H), 1.97 – 1.93 (m, 2H), 1.41 – 1.39 (m, 3H), 1.33 – 1.31 (m, 6H).

^{13}C NMR (75 MHz, CDCl$_3$) δ ppm 70.8, 59.8, 43.7, 27.6, 26.2, 20.7.

2-hydrazineyl-2,6-dimethylheptan-4-ol (3b)

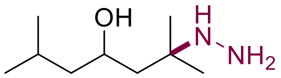

Purification by chromatography (EA), Yield: 46% (colorless oil, 24 mg).

^{1}H NMR (300 MHz, $CDCl_3$) δ = 9.00 (s, 1H), 4.57 – 4.44 (m, 1H), 1.96 – 1.84 (m, 3H), 1.78 – 1.61 (m, 2H), 1.48 (s, 1H), 1.32 (s, 6H), 0.99 – 0.91 (m, 6H).

^{13}C NMR (75 MHz, $CDCl_3$) δ ppm 72.7, 59.7, 43.8, 42.5, 27.7, 26.2, 24.2, 23.0, 22.4.

1-(1-hydrazineylcyclohexyl)propan-2-ol (3c)

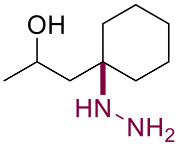

Purification by chromatography (EA), Yield: 52% (colorless oil, 14 mg).

^{1}H NMR (400 MHz, $CDCl_3$) δ = 9.05 (s, 1H), 4.68 – 4.39 (m, 1H), 2.42 (dd, *J*=14.4, 1.9, 1H), 1.90 – 1.42 (m, 9H), 1.41 (d, *J*=6.2, 3H), 1.36 – 1.24 (m, 2H).

^{13}C NMR (101 MHz, $CDCl_3$) δ ppm 70.2, 62.8, 36.2, 32.1, 24.5, 22.2, 20.5.

3-hydrazineyl-3-methylbutan-1-ol (3e)

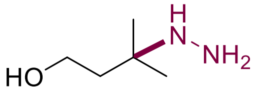

Purification by chromatography (EA), Yield: 36% (colorless oil, 3 mg).

^{1}H NMR (400 MHz, $CDCl_3$) δ = 8.81 (s, 1H), 4.42 – 4.29 (m, 2H), 2.10 (dd, *J*=6.1, 4.8, 2H), 1.35 (s, 6H).

^{13}C NMR (101 MHz, $CDCl_3$) δ ppm 63.5, 60.3, 36.4, 26.6.

1-cyclohexyl-3-hydrazineyl-3-methylbutan-1-ol (3j)

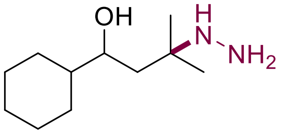

Purification by chromatography (EA); Yield: 47% (colorless oil, 9 mg).

^{1}H NMR (300 MHz, $CDCl_3$) δ = 9.33 (s, 1H), 4.87 (s, 2H), 4.19 (dd, *J*=7.2, 6.0, 1H), 2.03 – 1.85 (m, 6H), 1.84 – 1.63 (m, 4H), 1.59 – 1.52 (m, 1H), 1.30 (d, *J*=10.9, 6H), 1.15 – 1.03 (m, 2H).

^{13}C NMR (75 MHz, $CDCl_3$) δ ppm 78.31, 59.62, 41.43, 39.01, 28.21, 28.05, 27.75, 26.31, 26.02, 25.99, 25.87.

5-hydrazineyl-2,5-dimethylhexan-3-ol (3l)

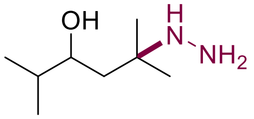

Purification by chromatography (EA); Yield: 58% (colorless oil, 11 mg)

^{1}H NMR (300 MHz, $CDCl_3$) δ = 8.78 (s, 1H), 4.28 – 4.12 (m, 1H), 1.95 – 1.90 (m, 2H), 1.73 (s, 1H), 1.33 (d, *J*=9.4, 6H), 1.01 (dd, *J*=9.6, 6.8, 6H).

^{13}C NMR (75 MHz, $CDCl_3$) δ ppm 78.8, 59.6, 38.9, 31.8, 27.8, 26.1, 17.9, 17.6.

2-hydrazineyl-2,7-dimethyloctan-4-ol (3m)

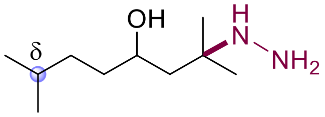

Purification by chromatography (EA); Yield: 48% (colorless oil, 27 mg).

^{1}H NMR (400 MHz, $CDCl_3$) δ = 8.70 (s, 1H), 4.55 – 4.26 (m, 1H), 2.02 – 1.88 (m, 2H), 1.80 – 1.50 (m, 5H), 1.32 (d, *J*=7.1, 6H), 0.91 (dd, *J*=6.6, 1.4, 6H).

^{13}C NMR (101 MHz, $CDCl_3$) δ ppm 74.7, 59.7, 42.0, 33.8, 32.6, 28.0, 27.7, 26.2, 22.6.

7-ethyl-2-hydrazineyl-2-methylundecan-4-ol (3n)

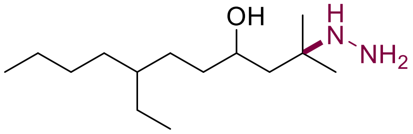

Purification by chromatography (EA), Yield: 58% (colorless oil, 40mg).

^{1}H NMR (400 MHz, $CDCl_3$) δ = 10.77 (s, 1H), 5.10 (s, 1H), 4.04 (s, 1H), 1.93 – 1.87 (m, 1H), 1.56 – 1.51 (m, 1H), 1.36 (s, 6H), 1.29 – 1.19 (m, 14H), 0.89 (t, *J*=6.1, 3H), 0.82 (t, *J*=7.3, 3H).

^{13}C NMR (101 MHz, $CDCl_3$) δ ppm 68.7, 66.3, 42.4, 38.9, 38.2, 35.4, 32.7, 29.0, 28.4, 25.7, 23.1, 14.1, 10.8.

7-ethyl-4-hydroxy-2-methylundecan-2-aminiumchloride (4n)

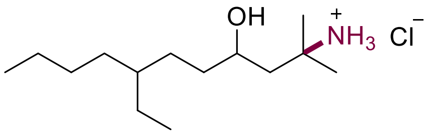

Purification by chromatography (hexane: EA = 1:1); Yield: 45% (white solid, 20mg).

^{1}H NMR (400 MHz, D_2O) δ = 3.96 (s, 1H), 2.99 (s, 3H), 2.13 – 1.99 (m, 1H), 1.73 (d, *J*=16.8, 1H), 1.49 (s, 3H), 1.35 (s, 3H), 1.33 – 1.17 (m, 14H), 0.86 (t, *J*=6.3, 3H), 0.82 (t, *J*=6.8, 3H).

^{13}C NMR (101 MHz, D_2O) δ ppm 68.5, 41.3, 39.9, 37.9, 34.6, 32.0, 28.2, 27.9, 25.2, 22.4, 21.7, 19.9, 13.4, 10.1.

LCMS: $[M]^+$ Calcd for $C_{14}H_{31}ON$ 229.24; Found 229.

6. Mechanistic studies-EPR spectroscopy

EPR measurements were recorded on a Bruker EMX CW-micro X-band spectrometer with a microwave power ≈ 6.9 mW, a modulation frequency of 100 kHz and modulation amplitude up to 5 G. The EPR spectrometer is equipped with a variable temperature control unit including a liquid N_2 cryostat and a temperature controller for recording the EPR spectra at low temperature down to 100 K. g values were calculated using the equation $h\nu = g\beta B_0$ with β, B_0 and ν being the Bohr magneton, resonance field and frequency, respectively. Calibration of the g values was performed using a DPPH standard (g = 2.0036 ± 0.0004). The simulated spectrum was acquired by using software package Easyspin.[3] All low-temperature EPR measurements were carried out using a standard quartz EPR tube. Prior to introducing the sample mixture, the tube was sealed with a rubber septum and thoroughly degassed using three cycles of argon purging and vacuum evacuation by Schlenk technique, to ensure an oxygen-free environment. For spin-trapping experiments involving 5,5-dimethyl-1-pyrroline N-oxide (DMPO), measurements were performed using an EPR flat cell. A similar degassing procedure was applied to the flat cell before sample loading. After the sample components were introduced, DMPO was added as the final component. The cell was then immediately subjected to the spectrometer.

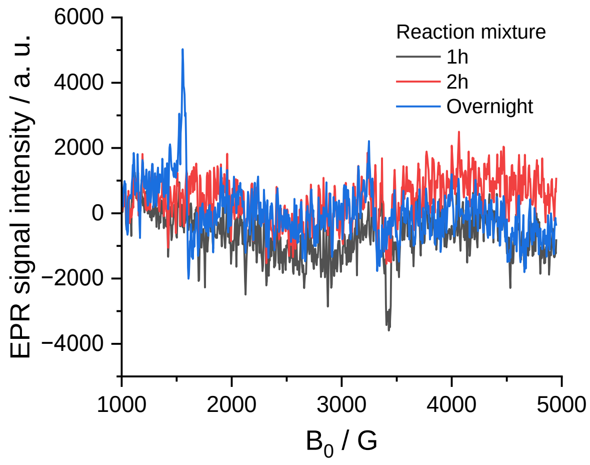

Figure S1. EPR spectra recorded at −173°C of reaction mixture at different time.

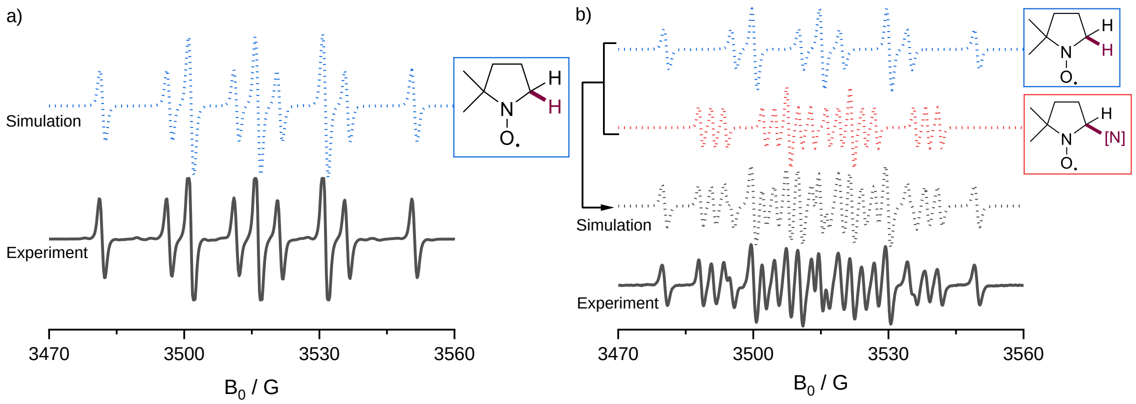

Figure S2. EPR spectra recorded at room temperature and the simulation spectra of the reaction mixture with DMPO after a) 0 minutes and b) 60 minutes.

7. DFT calculations

7.1. Computational Details

Computational study was carried out using density functional theory (DFT). In a first step, optimization calculations were performed using the BPW91 functional[(4,5)] and the split-valence double-ζ SVP basis set[(6,7)] in vacuum. Frequency calculations were also carried out to describe minima and first-order transition states (TS) with none and one imaginary frequency, respectively, also computing entropic and thermal contributions to the energy. Subsequently, single-point energy refinement calculations were conducted with the BPW91 functional and the split-valence triple-ζ TZVP basis set[(6,8)] including solvent effects through the polarizable continuum model (PCM)[(9,10)] with dichloromethane ($\varepsilon = 8.93$) as the solvent. All calculations have been performed using the Gaussian16 suite package,[(11)] visualizing and drawing structures through the facilities provided by the Jmol (http://www.jmol.org/) and CYLview(http://www.cylview.org) programs. The molecular electrostatic potential (MEP) mapped onto the van der Waals (vdW) isosurface in the aminoanion species **D1** was calculated using Gaussian 16. The geometry of aminoanion species **D1** was optimized at the BPW91 functional and the split-valence double-ζ SVP basis set level of theory in vacuum. The color range was chosen to clearly display both negative and positive electrostatic surface potential (ESP) regions (±0.015 a.u). The MEP-vdW isosurface highlights regions of negative (red) and positive (blue) potentials. In aminoanion species **D1**, the most negative region was found near the nitrogen atom of the azodicarboxylate moiety, supporting its role in base-assisted proton transfer, *i.e.*, it corresponds to a potential proton acceptor, nucleophilic site.

The catalytic cycle is initiated by the formation of the [Co(III)]–H and substrate complex. As described in our previous study[(12)], metal-hydrogen atom transfer (MHAT) can proceed *via* four distinct pathways: Markovnikov-MHAT, Markovnikov migratory insertion, anti-Markovnikov-MHAT and anti-Markovnikov migratory insertion. The calculated activation barriers, relative to substrate, for these pathways are 18.1, 24.1, 38.1, and 41.7 kcal mol^{-1}, respectively. These results indicate that the Markovnikov-MHAT pathway is energetically favored over the alternatives (see Figure S3).

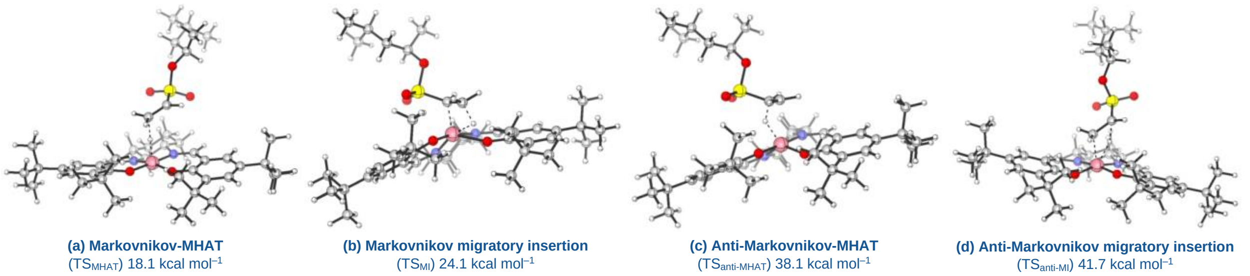

Figure S3. Representation for MHAT optimized transition states where [Co(III)]–H complex and substrate are involved: (a) Markovnikov-MHAT (TS_{MHAT}); (b) Markovnikov migratory insertion (TS_{MI}); (c) anti-Markovnikov migratory insertion ($TS_{anti\text{-}MI}$); and (d) anti-Markovnikov-MHAT ($TS_{anti\text{-}MHAT}$).

Following the formation of radical species **A1** *via* the Markovnikov-MHAT pathway, three possible intramolecular hydrogen atom transfer (HAT) routes were considered. Among them, the 1,6-HAT pathway is kinetically favored, with a calculated activation free energy of 8.6 kcal mol^{-1}, compared to 12.5 and 19.2 kcal mol^{-1} for the 1,5- and 1,7-HAT pathways, respectively. A comparative illustration of the transition states for all three HAT processes is provided in Figure S4. The preference for the 1,6-HAT pathway can be attributed to both enthalpic and entropic contributions. From an enthalpic standpoint, 1,6-HAT is favored due to its lower ring strain compared to the 1,5-HAT route. Entropically, it is more favorable than the 1,7-HAT pathway because it involves less structural reorganization.(13) This step leads to the formation of radical intermediate **B1** within the isopropyl moiety and is associated with a reaction free energy of –9.2 kcal mol^{-1} relative to the radical substrate.

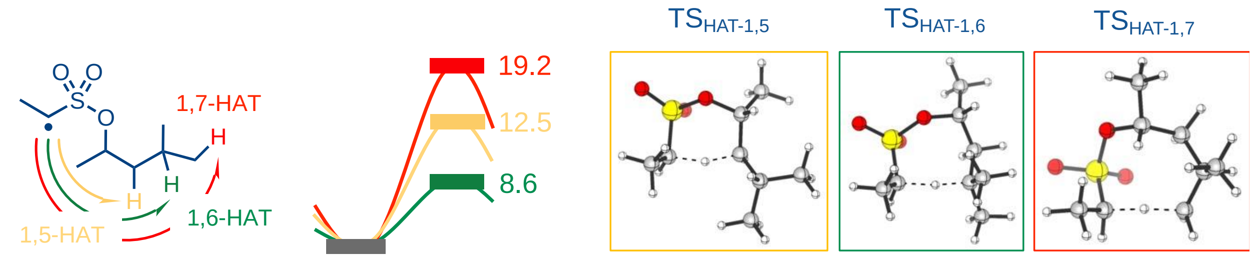

Figure S4. Representation of the transition states involving intramolecular HATs: 1,5- ($TS_{HAT\text{-}1,5}$), 1,6- ($TS_{HAT\text{-}1,6}$), and 1,7-HATs ($TS_{HAT\text{-}1,7}$) highlighted in yellow, green, and red colors, respectively.

In our previous study, we have performed DFT calculations on substrates **1l** and **1m**, corresponding to tertiary δ- and β-C–H abstraction pathways, respectively, were performed at the BPW91/TZVP//BPW91/SVP level of theory in dichloromethane at 45 °C. In both cases, a clear preference for 1,6-HAT over competing pathways is observed.[12] For substrate **1l**, the 1,6-HAT pathway is kinetically favored over the 1,5-HAT process by ΔΔG = 2.4 kcal mol^{-1}. Likewise, for substrate **1m**, the 1,6-HAT pathway is strongly preferred over the alternative 1,7-HAT route, with a ΔΔG of 13.2 kcal mol^{-1}.

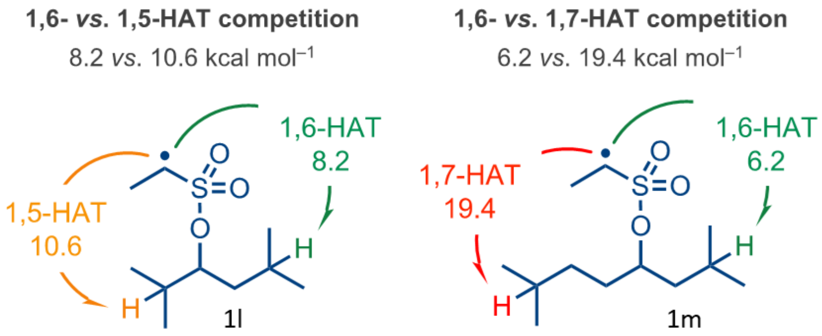

Figure S5. *DFT Analysis of Competing 1,5-, 1,6-, and 1,7-HATs Pathways*

To estimate the pK_a values, we employed a linear free energy relationship (LFER) approach based on computed reaction free energies. [14-19] All calculations were performed with the same computational protocol, *i.e.*, BPW91/TZVP-PCM(dichloromethane)//BPW91/SVP level of theory. The pK_a value of aminoanionic complex **D1** was estimated by considering acid-base equilibrium with various acids: dichloromethane (CH_2Cl_2; DCM), benzoic acid (C_6H_5COOH), formic acid (HCOOH), 4-nitrobenzoic acid [$C_6H_4(NO_2)COOH$)], 2,4-dinitrophenol [$C_6H_3(NO_2)_2OH$)], and hydrofluoric acid (HF), whose pK_a values were experimentally determined (range of 5-30 in DCM). A linear free energy plot was shown in Figure S6, where the OX-axis represents the calculated reaction free energy (ΔG) of the equilibrium shown in eqn. 1 with DCM of the PCM solvent model and the OY-axis represents reported pK_a values. In this regard, the intercept of the plot corresponds to pK_a values of aminoanionic complex **D1**.

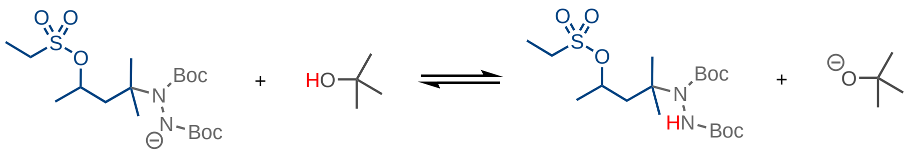

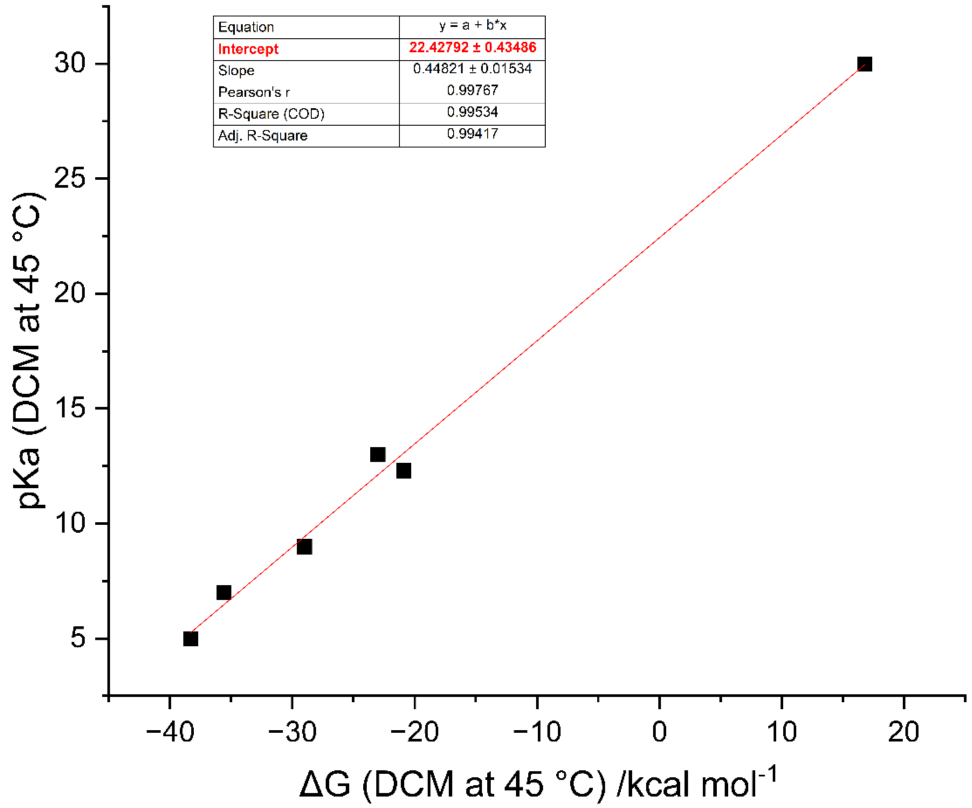

Figure S6. Linear free energy plot for eqn. 1 with pK_a in DCM solvent of aminoanion species **D1** with an estimated value of 22.4.

Table S1. Computed reaction free energies (ΔG) in DCM using the PCM solvent model, along with corresponding experimental pK_a values used for linear free energy relationship (LFER) analysis.

Solvents	ΔG (kcal mol^{-1})	pK_a
Dichloromethane (CH_2Cl_2)	16.8	30.0
Benzoic acid (C_6H_5COOH)	–23.0	13.0
Formic acid (HCOOH)	–20.9	12.3
4-Nitrobenzoic acid [$C_6H_4(NO_2)COOH$]	–29.0	9.0
2,4-Dinitrophenol [$C_6H_3(NO_2)_2OH$]	–35.6	7.0
Hydrofluoric acid (HF)	–38.3	5.0

7.2. Optimized Cartesian coordinates

```
[Co(III)]-H
Co,0.006924,0.718406,-0.060861
O,-1.296756,-0.647634,0.062232
O,1.297465,-0.666588,0.054708
N,1.262803,2.062257,0.248104
N,-1.262834,2.075309,-0.204422
C,-1.478594,4.644701,-0.141115
C,-0.627248,3.394314,-0.400989
C,0.626740,3.375439,0.488375
C,1.482638,4.632923,0.281271
C,0.639294,5.897821,0.524851
C,-0.632186,5.916131,-0.335937
C,-2.564964,1.925392,-0.293011
C,2.566371,1.912796,0.324227
C,-3.285756,0.694377,-0.179262
C,-2.602242,-0.561447,0.037452
C,-4.704418,0.754914,-0.255486
C,-3.424979,-1.736447,0.247530
C,-5.500884,-0.375704,-0.100783
C,-4.815688,-1.594132,0.161824
C,3.288076,0.688830,0.159633
C,2.600350,-0.574005,0.000515
C,4.708466,0.754523,0.178695
C,3.419007,-1.754815,-0.194360
C,5.501949,-0.377922,0.022462
C,4.811226,-1.606751,-0.169949
C,-7.041065,-0.279349,-0.202118
C,-7.571513,0.695356,0.880728
C,-7.431912,0.249544,-1.606294
C,-7.727527,-1.646369,0.004211
C,7.044733,-0.273577,0.052157
C,7.521959,0.664791,-1.086175
C,7.727672,-1.644797,-0.137393
C,7.495537,0.304662,1.418392
C,-2.786631,-3.107643,0.563847
C,-3.843979,-4.210708,0.790368
C,-1.893438,-3.549385,-0.622951
C,-1.945839,-2.994676,1.862860
C,2.771267,-3.135351,-0.442126
C,1.887606,-3.060764,-1.715209
C,1.919584,-3.539547,0.788233
C,3.820796,-4.245834,-0.668363
H,-0.274177,3.425806,-1.457548
H,-1.883651,4.608679,0.892961
H,-2.348818,4.675400,-0.826123
H,1.888998,4.635384,-0.752803
H,2.351698,4.634489,0.968372
H,1.252485,6.799615,0.326779
H,0.357176,5.946223,1.598721
H,-0.349914,6.004820,-1.407128
H,-1.242865,6.811314,-0.103158
H,0.268938,3.372331,1.544842
H,-3.180186,2.828196,-0.453753
H,3.178332,2.806625,0.538432
H,-5.161045,1.740271,-0.437249
H,-5.420243,-2.494341,0.309451
H,5.168895,1.745023,0.318086
H,5.412812,-2.509471,-0.314765
H,-8.675551,0.784168,0.823042
H,-7.149858,1.712355,0.762861
H,-7.309746,0.340864,1.897048
H,-8.533715,0.332536,-1.702764
H,-7.069261,-0.430345,-2.402056
H,-7.003957,1.251559,-1.803247
H,-7.516722,-2.070809,1.005163
H,-7.413138,-2.387701,-0.756062
H,-8.826173,-1.532594,-0.080565
H,8.627070,0.759006,-1.081131
H,7.100836,1.683751,-0.984515
H,7.216604,0.274734,-2.076966
H,7.450073,-2.360760,0.660606
H,8.828454,-1.524860,-0.104714
H,7.475344,-2.103731,-1.113173
H,7.172098,-0.348568,2.252481
H,7.072514,1.311684,1.599918
H,8.600152,0.394416,1.462397
H,-4.468865,-4.387807,-0.107320
H,-4.516302,-3.983182,1.641304
H,-3.331134,-5.164329,1.023690
H,-1.094311,-2.814185,-0.812956
H,-2.496484,-3.660237,-1.546233
H,-1.422447,-4.529314,-0.406142
H,-2.589020,-2.718007,2.722226
H,-1.153135,-2.234171,1.760281
H,-1.466974,-3.966576,2.097151
H,1.108620,-2.285778,-1.615910
H,1.390194,-4.034591,-1.896705
H,2.505131,-2.824748,-2.604875
H,2.553357,-3.616364,1.694380
H,1.447030,-4.528403,0.620802
H,1.123151,-2.801535,0.979614
H,4.476490,-4.393273,0.212645
H,4.463128,-4.046839,-1.549046
H,3.300937,-5.206409,-0.852560
H,0.188320,0.809804,-1.478480

Substrate
S,-1.640237,-0.415482,0.241005
O,-0.715530,0.767585,-0.481312
O,-1.248706,-0.521186,1.662398
O,-1.670525,-1.607834,-0.624561
C,1.562469,-0.122267,-0.574044
```

C,0.652428,0.962527,0.021289
C,1.014581,2.393418,-0.359139
C,3.051734,-0.077993,-0.163251
C,3.254781,-0.206212,1.356953
C,3.833639,-1.169368,-0.915116
C,-3.207136,0.438294,0.144632
C,-4.204324,-0.057240,-0.598374
H,0.609343,0.864014,1.125291
H,3.468824,0.903036,-0.482201
H,4.332557,-0.207747,1.613914
H,2.786445,0.624313,1.920389
H,2.818680,-1.152097,1.738560
H,4.914150,-1.128897,-0.673436
H,3.467563,-2.180570,-0.642894
H,3.729359,-1.062669,-2.013037
H,-3.259374,1.336610,0.776293
H,-5.187200,0.435958,-0.624599
H,-4.057299,-0.969231,-1.195872
H,2.007697,2.662176,0.047591
H,0.272866,3.103253,0.052341
H,1.043433,2.517295,-1.459177
H,1.480355,-0.061982,-1.679612
H,1.145446,-1.111209,-0.289522

TS Markovnikov-MHAT

Co,-0.845057,0.577155,0.885898
S,2.345187,-2.324878,-1.013003
O,-0.298368,2.263787,0.218566
O,-2.510468,0.904855,0.025055
O,3.038448,-2.909370,-2.405569
O,3.426050,-1.801599,-0.140351
O,1.405423,-3.335988,-0.476526
N,-1.518668,-0.854776,1.877865
N,0.798665,0.232728,1.702093
C,1.929776,-1.388483,3.355005
C,0.822276,-1.104018,2.333447
C,-0.578004,-1.292143,2.935069
C,-0.756765,-2.705181,3.506892
C,0.351268,-3.007773,4.532829
C,1.752757,-2.801343,3.940624
C,1.890427,0.950458,1.588711
C,-2.700449,-1.410995,1.746077
C,2.000805,2.228285,0.946516
C,0.862643,2.862386,0.319691
C,3.268131,2.869427,0.983288
C,1.048221,4.207420,-0.187489
C,3.469590,4.141261,0.452395
C,2.327148,4.774670,-0.107517
C,-3.715722,-1.031769,0.808585
C,-3.568968,0.137227,-0.031483
C,-4.891937,-1.827966,0.760210
C,-4.664283,0.448666,-0.929822
C,-5.948521,-1.535829,-0.098081
C,-5.786639,-0.389632,-0.923441
C,4.865323,4.805392,0.497273
C,5.329635,4.937429,1.970733
C,5.877097,3.927620,-0.283878
C,4.863553,6.214096,-0.133599
C,-7.212818,-2.425836,-0.128912
C,-6.816401,-3.872367,-0.522142
C,-8.258803,-1.921168,-1.145649
C,-7.870702,-2.436287,1.275078
C,-0.132698,5.000671,-0.791778
C,0.274967,6.426266,-1.224752
C,-0.665123,4.263374,-2.046239
C,-1.255673,5.138763,0.269709
C,-4.596018,1.670047,-1.874607
C,-3.377385,1.520022,-2.821796
C,-4.470616,2.967992,-1.036050
C,-5.856225,1.804466,-2.757771
C,0.078394,-0.923590,-1.590654
C,1.439755,-0.984866,-1.719060
C,4.395397,-3.470755,-2.317374
C,4.306523,-4.904584,-1.773741
C,5.640279,-5.670441,-1.621738
C,6.627058,-4.967212,-0.672902
C,5.365944,-7.111741,-1.158076
H,0.921157,-1.848001,1.512457
H,1.900276,-0.626989,4.164175
H,2.921502,-1.319539,2.868259
H,-0.719978,-3.443188,2.677779
H,-1.746796,-2.810472,3.993647
H,0.238292,-4.045394,4.906093
H,0.221636,-2.345675,5.416331
H,1.926513,-3.546523,3.135803
H,2.527964,-2.987104,4.710831
H,-0.676537,-0.562133,3.772453
H,2.817861,0.555241,2.036370
H,-2.957796,-2.246721,2.420186
H,4.097499,2.323450,1.458523
H,2.452065,5.785378,-0.508080
H,-4.942099,-2.699084,1.431730
H,-6.601880,-0.139747,-1.609305
H,6.332228,5.408431,2.026033
H,5.397324,3.952745,2.472265
H,4.625682,5.562334,2.554697
H,6.889882,4.379189,-0.260313
H,5.574976,3.821771,-1.344413
H,5.955720,2.909496,0.143884
H,4.181749,6.906684,0.397431
H,4.566783,6.191209,-1.200354
H,5.880620,6.650250,-0.083591
H,-7.707862,-4.531796,-0.545821
H,-6.096239,-4.312110,0.194651
H,-6.346408,-3.897006,-1.524928
H,-8.609856,-0.898107,-0.907689
H,-9.145600,-2.584926,-1.134223
H,-7.864169,-1.916712,-2.180434
H,-8.168667,-1.414489,1.581956
H,-7.184560,-2.830650,2.049377

H,-8.778543,-3.073387,1.279084
H,1.048905,6.422302,-2.017648
H,0.650491,7.033397,-0.377405
H,-0.610436,6.949449,-1.636149
H,-1.006318,3.247157,-1.789411
H,0.124459,4.187123,-2.820517
H,-1.518801,4.817055,-2.486516
H,-0.891422,5.699472,1.153675
H,-1.610768,4.149499,0.605431
H,-2.118038,5.693428,-0.151732
H,-2.440395,1.453564,-2.243067
H,-3.304776,2.392517,-3.501430
H,-3.476478,0.609931,-3.446700
H,-5.353467,3.094604,-0.378163
H,-4.414693,3.852879,-1.701719
H,-3.565280,2.948712,-0.406659
H,-6.776862,1.951109,-2.159153
H,-6.007286,0.925938,-3.415889
H,-5.748793,2.690468,-3.413773
H,-0.341114,-0.294587,-0.219478
H,-0.469354,-0.137297,-2.128318
H,-0.476322,-1.838625,-1.332922
H,2.075064,-0.174334,-2.101738
H,6.117648,-5.733829,-2.624680
H,7.557269,-5.558320,-0.559237
H,6.921236,-3.962312,-1.033635
H,6.186251,-4.840521,0.337258
H,6.304009,-7.696928,-1.086531
H,4.886992,-7.123946,-0.157425
H,4.689564,-7.642970,-1.856562
H,4.966512,-2.827953,-1.616956
H,6.024248,-3.670806,-3.743336
H,4.928347,-2.292239,-4.064202
H,4.397586,-3.964299,-4.442996
H,3.624998,-5.475361,-2.438789
H,3.804764,-4.863231,-0.784149

Radical species I

S,1.665045,-0.569073,0.010758
O,0.633319,0.737253,0.232230
O,1.082025,-1.457407,-1.018067
O,2.015600,-1.066375,1.353594
C,4.080049,0.894863,0.188249
C,3.049383,0.266065,-0.670643
C,-0.742584,0.637853,-0.272291
C,-1.581799,-0.093991,0.791199
C,-1.149266,2.069992,-0.601665
C,-3.038393,-0.505352,0.445461
C,-3.144938,-1.275127,-0.883261
C,-4.067892,0.636683,0.524119
H,3.831829,1.960914,0.403560
H,4.155374,0.371631,1.160158
H,5.068727,0.891271,-0.312452
H,2.985099,0.410550,-1.759310
H,-0.708462,0.030670,-1.199776
H,-1.025036,-1.021967,1.032005
H,-0.409310,2.529435,-1.283919
H,-2.133966,2.089941,-1.102989
H,-2.976082,-0.616299,-1.759453
H,-2.406388,-2.098836,-0.939057
H,-4.153809,-1.717271,-1.003095
H,-5.098784,0.235453,0.455801
H,-3.989572,1.191120,1.480325
H,-3.954914,1.370127,-0.298107
H,-1.211816,2.690306,0.313172
H,-1.574792,0.514345,1.720064
H,-3.313741,-1.219902,1.251872

[Co(II)] metalloradical

Co,-0.000009,0.699415,-0.000080
O,-1.299957,-0.637847,0.125781
O,1.299963,-0.637831,-0.125948
N,1.240464,2.043332,0.292832
N,-1.240507,2.043314,-0.292939
C,-1.464137,4.615838,-0.313906
C,-0.597452,3.360502,-0.484907
C,0.597413,3.360534,0.484712
C,1.464106,4.615850,0.313589
C,0.602856,5.882220,0.475123
C,-0.602869,5.882180,-0.475574
C,-2.549844,1.904799,-0.378508
C,2.549788,1.904794,0.378613
C,-3.280895,0.689428,-0.203593
C,-2.605877,-0.557086,0.082458
C,-4.699810,0.746397,-0.287975
C,-3.426188,-1.725271,0.328449
C,-5.495898,-0.376261,-0.082976
C,-4.815734,-1.586886,0.230528
C,3.280857,0.689430,0.203738
C,2.605877,-0.557061,-0.082507
C,4.699762,0.746390,0.288310
C,3.426223,-1.725211,-0.328548
C,5.495878,-0.376244,0.083288
C,4.815757,-1.586835,-0.230445
C,-7.036340,-0.280598,-0.185671
C,-7.561714,0.728712,0.867678
C,-7.428727,0.205195,-1.604849
C,-7.725776,-1.638822,0.064267
C,7.036306,-0.280592,0.186199
C,7.561823,0.728852,-0.866949
C,7.725776,-1.638783,-0.063821
C,7.428501,0.205019,1.605493
C,-2.783471,-3.085622,0.679872
C,-3.838682,-4.182123,0.944667
C,-1.903096,-3.556751,-0.506214
C,-1.928457,-2.941150,1.966706
C,2.783550,-3.085520,-0.680212
C,1.928574,-2.940875,-1.967053
C,1.903150,-3.556860,0.505773
C,3.838798,-4.181949,-0.945145

H,-0.175084,3.367593,-1.516371
H,-1.940584,4.604167,0.689812
H,-2.283857,4.630830,-1.059324
H,1.940559,4.604061,-0.690123
H,2.283823,4.630917,1.059010
H,1.226779,6.782771,0.306952
H,0.243449,5.947164,1.524770
H,-0.243459,5.947003,-1.525227
H,-1.226774,6.782760,-0.307499
H,0.175052,3.367716,1.516180
H,-3.154449,2.803152,-0.592142
H,3.154369,2.803134,0.592373
H,-5.156268,1.721762,-0.516856
H,-5.423092,-2.479385,0.409290
H,5.156191,1.721733,0.517344
H,5.423139,-2.479310,-0.409244
H,-8.665715,0.817804,0.810097
H,-7.137811,1.740350,0.716864
H,-7.297965,0.405541,1.893864
H,-8.530613,0.286141,-1.701384
H,-7.068492,-0.499442,-2.379903
H,-7.000746,1.200298,-1.833760
H,-7.516294,-2.031485,1.078334
H,-7.413152,-2.404830,-0.671892
H,-8.824110,-1.525031,-0.024182
H,8.665815,0.817943,-0.809203
H,7.137896,1.740471,-0.716069
H,7.298220,0.405810,-1.893214
H,7.413032,-2.404891,0.672184
H,8.824096,-1.525011,0.024815
H,7.516449,-2.031306,-1.077975
H,7.068154,-0.499714,2.380407
H,7.000495,1.200095,1.834469
H,8.530374,0.285946,1.702191
H,-4.469578,-4.385035,0.056755
H,-4.505252,-3.929526,1.793023
H,-3.324351,-5.128701,1.202050
H,-1.105548,-2.827050,-0.724419
H,-2.516684,-3.690174,-1.419498
H,-1.429919,-4.531172,-0.270606
H,-2.562639,-2.644763,2.826170
H,-1.137134,-2.182914,1.839837
H,-1.448203,-3.908131,2.218308
H,1.137215,-2.182692,-1.840088
H,1.448364,-3.907834,-2.218823
H,2.562773,-2.644329,-2.826449
H,2.516710,-3.690402,1.419058
H,1.430009,-4.531259,0.270002
H,1.105572,-2.827216,0.724056
H,4.469663,-4.384988,-0.057240
H,4.505394,-3.929191,-1.793433
H,3.324504,-5.128500,-1.202703

TSHAT-1,5
S,1.873788,0.096873,0.204005
O,0.899141,0.802893,-0.955692
O,1.762097,0.837080,1.477914
O,3.163984,-0.082559,-0.477294
C,1.111074,-2.435656,-0.737858
C,0.980489,-1.447740,0.390863
C,-0.477447,1.183985,-0.583854
C,-1.180643,0.144768,0.302372
C,-0.482765,2.599274,-0.005972
C,-2.468734,-0.492917,-0.208192
C,-2.906854,-1.665502,0.686768
C,-3.606727,0.546051,-0.339343
H,0.048245,2.621681,0.963106
H,-1.235844,0.449396,1.366532
H,0.445033,-3.304565,-0.571614
H,0.854682,-1.973930,-1.710193
H,2.151491,-2.815170,-0.814406
H,1.129183,-1.820102,1.420517
H,-0.966429,1.185312,-1.579957
H,-0.257062,-0.822187,0.402915
H,0.021531,3.297335,-0.700938
H,-1.521986,2.949450,0.147431
H,-2.267271,-0.892268,-1.227930
H,-3.829242,-2.140670,0.299409
H,-2.126255,-2.448463,0.749420
H,-3.117917,-1.320558,1.719541
H,-4.526421,0.068826,-0.732898
H,-3.852786,0.992065,0.645417
H,-3.341685,1.370986,-1.028563

TSHAT-1,6
S,-1.502313,-0.196189,-0.270385
O,-0.725676,0.650384,0.950824
O,-1.439954,0.569157,-1.535410
O,-2.789274,-0.573098,0.335939
C,-0.589980,-2.676038,0.651160
C,-0.431878,-1.621558,-0.413299
C,0.317327,1.639164,0.661595
C,1.381969,1.150325,-0.338498
C,-0.307865,2.977570,0.270643
C,1.926718,-0.262874,-0.126689
C,2.856682,-0.718073,-1.242209
C,2.431997,-0.578604,1.273026
H,-0.822910,2.898961,-0.704110
H,2.226003,1.876667,-0.281327
H,0.174437,-3.467207,0.522777
H,-0.484315,-2.248791,1.666238
H,-1.589040,-3.156580,0.596537
H,-0.453639,-1.957090,-1.465933
H,0.789217,1.742031,1.660329
H,0.978179,1.232584,-1.366979
H,-1.043435,3.292988,1.033960
H,0.473031,3.759935,0.198064
H,0.844796,-0.989857,-0.283142
H,3.125421,-1.788273,-1.141064
H,2.405082,-0.568938,-2.242732

H,3.808117,-0.140808,-1.223797
H,2.762815,-1.632361,1.356545
H,3.309988,0.057648,1.526817
H,1.662688,-0.402901,2.048777

TSHAT-1,7

S,-1.629754,0.032937,-0.432644
O,-0.676058,1.298868,0.082384
O,-1.293848,-0.323546,-1.827906
O,-2.989278,0.480103,-0.088624
C,-1.298707,-1.150635,2.102825
C,-1.122804,-1.333949,0.614661
C,0.767073,1.193825,0.307611
C,1.581153,0.625459,-0.881962
C,1.175761,2.614622,0.690863
C,2.263649,-0.745863,-0.619423
C,1.280025,-1.900112,-0.522901
C,3.254921,-0.707250,0.557849
H,2.371766,1.355211,-1.149870
H,1.044668,3.299525,-0.169055
H,-0.941588,-2.049520,2.642470
H,-0.747334,-0.272608,2.488256
H,-2.369316,-1.005303,2.356125
H,-1.632858,-2.219174,0.189128
H,0.905468,0.539397,1.194853
H,0.917468,0.550786,-1.764088
H,0.560003,2.988578,1.529895
H,2.239552,2.633196,0.996381
H,1.648832,-2.804713,-0.004692
H,0.156310,-1.571407,0.194781
H,0.763400,-2.139926,-1.469010
H,3.819201,-1.657659,0.629512
H,3.992582,0.110834,0.438098
H,2.746144,-0.559490,1.531912
H,2.868816,-0.944704,-1.536359

Radical species II

S,-1.593343,-0.629538,0.040645
O,-0.690485,0.779620,0.069588
O,-1.188236,-1.479627,1.180545
O,-1.602833,-1.176245,-1.330587
C,-3.699452,1.068768,-0.694176
C,-3.214069,0.117592,0.392205
C,0.705930,0.667044,0.500237
C,1.578208,0.102779,-0.649639
C,1.106842,2.061913,0.961731
C,2.992825,-0.207438,-0.239337
C,3.272893,-1.475861,0.510872
C,4.143498,0.555969,-0.820728
H,-4.717235,1.427565,-0.446703
H,-3.033384,1.946112,-0.783676
H,-3.738561,0.556854,-1.673263
H,-3.867459,-0.770447,0.504927
H,0.731696,-0.036079,1.358540
H,1.073758,-0.818714,-1.016459
H,0.463172,2.402679,1.794275
H,2.156294,2.049190,1.312294
H,-3.101448,0.592709,1.384553
H,4.088096,-1.351669,1.255194
H,2.379919,-1.861733,1.040538
H,3.609021,-2.293448,-0.174258
H,5.008999,0.598119,-0.126225
H,4.529681,0.081521,-1.757928
H,3.868115,1.595148,-1.089742
H,1.023981,2.792088,0.133084
H,1.551246,0.828136,-1.488840

Radical species III

S,4.002478,0.076075,0.346488
O,3.349943,-1.146807,-0.595681
O,3.786480,-0.247121,1.770085
O,3.577261,1.371779,-0.217109
O,0.062182,2.354892,0.935669
O,-0.169840,0.567916,2.241109
O,-3.636108,-0.048745,-0.282426
O,-3.245761,-2.232615,0.269927
N,-1.479486,-0.722736,0.096807
N,-1.237433,0.577463,0.190001
C,6.083825,0.041539,-1.535913
C,5.756251,-0.184284,-0.065711
C,2.256928,-1.958096,-0.045763
C,0.945918,-1.189792,-0.334417
C,2.467792,-3.333725,-0.672335
C,-0.408319,-1.846084,0.064160
C,-0.361923,-2.516990,1.452028
C,-0.813827,-2.842163,-1.047849
C,-5.119450,-0.161751,-0.290343
C,-5.618484,-0.542838,1.110546
C,-5.558553,-1.166733,-1.365017
C,-5.558012,1.260515,-0.659636
C,-0.183990,3.202264,-0.250659
C,-1.666123,3.600256,-0.297936
C,0.277072,2.502549,-1.537898
C,0.706456,4.419663,0.038738
C,-2.867321,-1.095886,0.033271
C,-0.402414,1.110430,1.164298
H,7.173871,-0.069528,-1.695022
H,5.562346,-0.690533,-2.179106
H,5.787819,1.058958,-1.851056
H,6.268609,0.538155,0.600581
H,2.408858,-2.023165,1.050662
H,1.072306,-0.238953,0.207423
H,3.499872,-3.673604,-0.465202
H,1.778052,-4.082533,-0.242192
H,5.986915,-1.205356,0.291390
H,0.340091,-3.372811,1.443550
H,-0.046799,-1.789143,2.220031
H,-1.361266,-2.907156,1.712984
H,-1.706464,-3.426467,-0.777379
H,-1.010821,-2.307142,-1.997430

H,0.015546,-3.544561,-1.232394
H,2.327184,-3.308734,-1.769876
H,0.925318,-0.938990,-1.413950
H,-6.661918,1.314967,-0.709244
H,-5.149260,1.554238,-1.644685
H,-5.205123,1.988897,0.093909
H,-5.140256,-0.888321,-2.351458
H,-6.662422,-1.158596,-1.448968
H,-5.233418,-2.191020,-1.114119
H,-6.724599,-0.503019,1.130782
H,-5.236993,0.170851,1.865534
H,-5.300546,-1.562120,1.389708
H,1.317596,2.143659,-1.429246
H,0.242952,3.219891,-2.381267
H,-0.378809,1.649320,-1.790208
H,-1.838217,4.313887,-1.127361
H,-1.963151,4.094506,0.647044
H,-2.307875,2.715010,-0.453449
H,0.613958,5.166954,-0.772435
H,1.764554,4.108283,0.118147
H,0.412650,4.896241,0.992871

Aminoanion species D1

S,4.707392,-0.143122,0.409501
O,3.474122,-0.168463,-0.692922
O,5.908708,0.312230,-0.317207
O,4.737285,-1.414717,1.171527
O,-3.387047,-0.484760,0.250496
O,-2.654974,-2.649545,-0.034696
O,-0.621832,2.616468,0.350861
O,-0.158932,0.789142,1.545184
N,-1.227486,-0.843228,-0.343681
N,-1.172553,0.552423,-0.567554
C,3.060130,0.907791,2.472045
C,4.191642,1.224775,1.503697
C,2.598373,-1.357264,-0.806548
C,1.181017,-0.773860,-0.869619
C,3.100293,-2.164908,-2.000039
C,-0.034064,-1.726250,-0.674714
C,0.227802,-2.670778,0.519357
C,-0.353646,-2.515026,-1.964870
C,-1.201799,3.378279,-0.735791
C,-2.721276,3.144340,-0.827641
C,-0.508707,3.061312,-2.075318
C,-0.912254,4.835035,-0.325525
C,-2.423393,-1.427216,-0.044253
C,-0.658175,1.214276,0.484851
C,-4.717977,-0.899835,0.663181
C,-4.656103,-1.727264,1.961734
C,-5.430257,-1.667762,-0.467518
C,-5.431060,0.437541,0.925722
H,2.981416,1.734814,3.205367
H,2.065837,0.822281,1.986212
H,3.276439,-0.022307,3.030815
H,5.144137,1.465122,2.018333
H,2.735943,-1.945095,0.120859
H,1.138865,-0.054442,-0.035353
H,4.166018,-2.430520,-1.862140
H,2.525843,-3.104182,-2.107465
H,3.960812,2.054398,0.808443
H,1.067713,-3.363581,0.309598
H,0.464645,-2.068312,1.416491
H,-0.672423,-3.275061,0.722559
H,-1.271357,-3.111904,-1.816818
H,-0.512255,-1.816477,-2.809793
H,0.468620,-3.205626,-2.235743
H,2.998665,-1.587124,-2.939375
H,1.062297,-0.195700,-1.805482
H,-6.471342,0.262763,1.265020
H,-5.456649,1.051926,0.005995
H,-4.894654,1.013538,1.703193
H,-5.440282,-1.058324,-1.392511
H,-6.480510,-1.884100,-0.184452
H,-4.904311,-2.615582,-0.674459
H,-5.679670,-1.935522,2.334054
H,-4.109096,-1.164309,2.742833
H,-4.131355,-2.681384,1.782884
H,0.584828,3.217835,-1.986167
H,-0.890447,3.721011,-2.881886
H,-0.695938,2.003508,-2.334144
H,-3.167815,3.769394,-1.628472
H,-3.206328,3.409783,0.132523
H,-2.909450,2.075043,-1.033930
H,-1.311377,5.547549,-1.075264
H,0.179206,4.998084,-0.229251
H,-1.375937,5.056144,0.655700

Aminated product V

S,3.888897,0.303478,0.178146
O,3.633657,-1.255757,-0.367462
O,3.564558,0.370986,1.618116
O,3.276403,1.256558,-0.771355
O,-3.369842,-0.494993,-0.558591
O,-2.963425,-2.479223,0.521825
O,-0.877747,2.317891,-0.558987
O,-1.130164,1.078095,1.375886
N,-1.227978,-1.169481,-0.291607
N,-0.912871,0.102073,-0.709299
C,-2.573742,-1.467214,-0.046053
C,-0.997195,1.182682,0.168357
C,-4.834636,-0.523580,-0.356898
C,-5.158902,-0.490003,1.144137
C,-5.437508,-1.750324,-1.058476
C,-5.290066,0.772298,-1.041890
C,-0.658454,3.627918,0.094219
C,0.602595,3.569509,0.968895
C,-1.907357,4.020919,0.896944
C,-0.453088,4.566744,-1.101762
C,6.145387,0.210851,-1.485763
C,5.694861,0.344482,-0.036760

C,2.543148,-2.029888,0.250300
C,1.221692,-1.542684,-0.374263
C,2.951652,-3.482939,0.034962
C,-0.119585,-2.124228,0.157878
C,-0.124631,-2.205204,1.699242
C,-0.398566,-3.499681,-0.486744
H,7.246140,0.314926,-1.543438
H,5.867002,-0.774474,-1.901698
H,5.687551,0.998275,-2.112271
H,5.965867,1.323850,0.405558
H,2.554116,-1.795549,1.333608
H,1.205813,-0.451437,-0.211655
H,3.984184,-3.637904,0.400346
H,2.289663,-4.165081,0.598622
H,6.080293,-0.452592,0.626042
H,0.618807,-2.941314,2.061781
H,0.097945,-1.217339,2.142539
H,-1.118292,-2.530504,2.053170
H,-1.369170,-3.900656,-0.151489
H,-0.411122,-3.414489,-1.590950
H,0.385835,-4.225845,-0.210807
H,2.913873,-3.761182,-1.035588
H,1.282478,-1.683670,-1.473367
H,-6.390091,0.872395,-0.976380
H,-5.003069,0.772319,-2.110696
H,-4.829007,1.654304,-0.559234
H,-5.154967,-1.763075,-2.129098
H,-6.542438,-1.709753,-0.997897
H,-5.090489,-2.685609,-0.586939
H,-6.249695,-0.364931,1.287378
H,-4.645043,0.361337,1.629608
H,-4.841894,-1.422799,1.641169
H,-2.803172,4.026762,0.246273
H,-1.780377,5.039176,1.313232
H,-2.075439,3.316652,1.730145
H,0.849670,4.585737,1.332559
H,1.462908,3.187159,0.387946
H,0.452611,2.911749,1.842650
H,-0.293353,5.603707,-0.750280
H,-1.338868,4.555881,-1.765264
H,0.429269,4.257681,-1.693293
H,-0.975119,0.306087,-1.709663

TSCN1 for C-N coupling

S,0.904536,-0.384068,-0.369216
O,0.327996,-1.724819,-0.138425
O,2.306906,-0.593650,-1.283014
O,1.230899,0.504200,0.769601
O,-2.040920,2.403124,1.800683
O,-3.394341,-1.193637,-1.624408
O,-3.212330,-1.688140,0.618422
O,-1.052509,3.004036,-0.185803
N,-2.973450,0.451018,-0.051394
N,-1.795130,0.853577,0.092256
C,-0.532097,-0.213466,-2.784336
C,-0.053763,0.482315,-1.562013
C,3.597585,-0.496408,-0.592722
C,4.585992,-0.060856,-1.669622
C,-1.673925,2.188891,0.666632
C,3.879632,-1.843939,0.097784
C,5.078460,-1.950491,1.078123
C,6.457336,-2.073068,0.404254
C,5.070864,-0.850781,2.155379
C,-3.173343,-0.917601,-0.461031
C,-3.571525,-3.140513,0.551669
C,-3.421925,-3.579001,2.011827
C,-0.634397,4.381923,0.229529
C,-1.882125,5.209506,0.562301
C,-2.576030,-3.874215,-0.355092
C,-5.021173,-3.267595,0.068149
C,0.061854,4.911465,-1.028672
C,0.343250,4.277867,1.406539
H,-0.786050,-1.268882,-2.583422
H,-1.428152,0.289344,-3.195915
H,0.255503,-0.192991,-3.572750
H,3.500233,0.301882,0.171385
H,2.962830,-2.093505,0.668638
H,4.221836,0.855131,-2.172093
H,5.574342,0.160970,-1.227569
H,5.326977,0.143228,1.735223
H,4.079008,-0.760880,2.640531
H,5.815736,-1.067556,2.946587
H,7.232580,-2.339173,1.150412
H,6.461854,-2.860410,-0.375488
H,6.783530,-1.128053,-0.072748
H,4.716781,-0.849358,-2.435860
H,3.966856,-2.625485,-0.686384
H,-3.679447,-4.650268,2.113279
H,-4.091828,-2.993244,2.669014
H,-2.381241,-3.433172,2.356488
H,-5.706702,-2.698518,0.724944
H,-5.326861,-4.331359,0.093806
H,-5.130434,-2.900696,-0.967987
H,-2.723261,-4.966559,-0.249933
H,-1.537299,-3.628089,-0.067449
H,-2.721608,-3.601762,-1.414831
H,-0.161772,3.917241,2.319271
H,0.774938,5.275903,1.614381
H,1.168018,3.581832,1.164789
H,0.395864,5.953129,-0.863653
H,-0.626821,4.901030,-1.894583
H,0.950244,4.300901,-1.278015
H,-1.584556,6.254736,0.772991
H,-2.403552,4.809496,1.449165
H,-2.585320,5.219998,-0.292455
H,4.909889,-2.915139,1.605320
H,0.151753,1.560110,-1.552295

TSCN2 for C-N coupling

S,3.768889,0.590546,-0.425412

O,3.466824,-1.048736,-0.661732
O,4.271658,0.781912,0.948662
O,2.623823,1.371328,-0.931799
O,-3.070487,-0.634253,-0.665759
O,-2.903604,-2.335928,0.857723
O,-0.734460,2.864370,1.338707
O,0.300040,1.133248,2.265053
N,-1.669086,-0.425256,1.204453
N,-1.415936,0.722205,0.689417
C,-2.588237,-1.224445,0.443353
C,-0.561149,1.539861,1.503987
C,-4.088282,-1.289873,-1.522104
C,-5.370721,-1.533665,-0.712800
C,-3.516458,-2.587947,-2.111457
C,-4.324368,-0.249265,-2.624071
C,-1.596113,3.560547,0.351596
C,-3.074270,3.240734,0.613019
C,-1.158596,3.198983,-1.074581
C,-1.297385,5.035818,0.657157
C,4.755309,0.487761,-3.049963
C,5.150989,0.733590,-1.599768
C,2.853030,-1.784202,0.435373
C,1.312180,-1.542170,0.405333
C,3.281066,-3.233992,0.244224
C,0.543592,-2.327481,1.422366
C,0.658224,-1.978995,2.866585
C,-0.125117,-3.600793,1.049593
H,5.627751,0.665192,-3.708153
H,4.412219,-0.551978,-3.200223
H,3.943184,1.172794,-3.355428
H,5.504439,1.769094,-1.423335
H,3.265818,-1.387380,1.386222
H,1.160610,-0.460389,0.585900
H,4.384448,-3.310052,0.241475
H,2.893235,-3.864252,1.066669
H,5.921628,0.036579,-1.221184
H,1.610198,-2.390384,3.283132
H,0.671233,-0.882651,3.011562
H,-0.166578,-2.421356,3.456081
H,-1.232696,-3.467648,1.140238
H,0.092309,-3.923890,0.014685
H,0.133591,-4.418796,1.756479
H,2.901619,-3.639148,-0.713846
H,0.963730,-1.776012,-0.620212
H,-5.074156,-0.622876,-3.347112
H,-3.386101,-0.037418,-3.170495
H,-4.695810,0.700078,-2.194879
H,-2.566268,-2.386615,-2.642716
H,-4.232475,-3.011369,-2.842154
H,-3.335159,-3.338354,-1.322875
H,-6.161760,-1.926741,-1.380379
H,-5.736932,-0.587339,-0.270461
H,-5.199953,-2.263472,0.097425
H,-0.071385,3.363057,-1.198300
H,-1.692868,3.841525,-1.801553
H,-1.384892,2.142028,-1.298086
H,-3.714487,3.876381,-0.029625
H,-3.337717,3.446354,1.668526
H,-3.291516,2.181761,0.385344
H,-1.882285,5.693072,-0.014106
H,-0.222228,5.251804,0.512943
H,-1.560326,5.278693,1.704080

8. Single crystal structure

X-ray crystal structure analysis of **2a:**

Diffraction data were collected on a Bruker Kappa APEX II Duo diffractometer. The structure was solved by intrinsic phasing[20] and refined by full-matrix least-squares procedures on F^2 XP (Bruker AXS) was used for graphical representations[21].

CCDC 2497523 contains the supplementary crystallographic data for this paper. These data are provided free of charge by the joint Cambridge Crystallographic Data Centre and Fachinformationszentrum Karlsruhe Access Structures service www.ccdc.cam.ac.uk/structures.

Crystal data of **2a**: $C_{12}H_{22}N_2O_4$, M = 258.31, monoclinic, space group $P2_1/c$, a = 11.9794(12), b = 15.5993(15), c = 15.7039(15) Å, β = 91.7651(17)°, V = 2933.2(5) Å^3, T = 150(2) K, Z = 8, 45727 reflections measured, 7083 independent reflections (R_{int} = 0.0252), final R values (I > 2σ(I)): R_1 = 0.0374, wR_2 = 0.0899, final R values (all data): R_1 = 0.0506, wR_2 = 0.0990, 449 parameters.

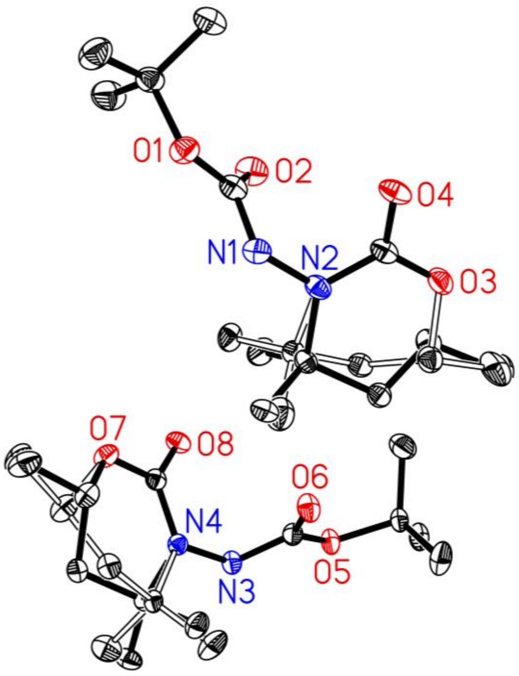

Figure S7. The molecular structure of **2a**. Displacement ellipsoids correspond to 30% probability. Hydrogen atoms are omitted for clarity. Lower occupied parts of disorder are shown with unfilled lines.

9. References

1. Jue, Z.; Huang, Y.; Qian, J.; Hu, P. Visible Light-Induced Unactivated δ-C(sp3)-H Amination of Alcohols Catalyzed by Iron. *ChemSusChem* **2022**, *15* (20), e202201241.
2. Yang, X.; Toste, F. D. Direct Asymmetric Amination of α-Branched Cyclic Ketones Catalyzed by a Chiral Phosphoric Acid. *J. Am. Chem. Soc.* **2015**, *137* (9), 3205-3208.
3. Stoll, S.; Schweiger, A. EasySpin, a comprehensive software package for spectral simulation and analysis in EPR. *Magn. Reson* **2006**, *178* (1), 42-55.
4. Becke, A. D. Density-functional exchange-energy approximation with correct asymptotic behavior. *PHYS REV A* **1988**, *38* (6), 3098-3100.
5. Perdew, J. P.; Wang, Y. Accurate and simple analytic representation of the electron-gas correlation energy. *PHYS REV B* **1992**, *45* (23), 13244-13249.
6. Ahlrichs, R.; Bär, M.; Häser, M.; Horn, H.; Kölmel, C. Electronic structure calculations on workstation computers: The program system turbomole. *Chem. Phys. Lett* **1989**, *162* (3), 165-169.
7. Pople, J. A. Nobel Lecture: Quantum chemical models. *Rev Mod Phys* **1999**, *71* (5), 1267-1274.
8. Perdew, J. P.; Burke, K.; Ernzerhof, M. Generalized Gradient Approximation Made Simple. *Phys. Rev. Lett* **1996**, *77* (18), 3865-3868.
9. Tomasi, J.; Mennucci, B.; Cammi, R. Quantum Mechanical Continuum Solvation Models. *Chem. Rev.* **2005**, *105* (8), 2999-3094.
10. Tomasi, J.; Persico, M. Molecular Interactions in Solution: An Overview of Methods Based on Continuous Distributions of the Solvent. *Chem. Rev.* **1994**, *94* (7), 2027-2094.
11. Frisch et al., Gaussian, Inc., Wallingford CT, 2016
12. Dam, P.; Amarasinghe, K. N.; Wang, C.; Bokareva, O. S.; Azofra, L. M.; El-Sepelgy, O. Cobalt-Catalyzed γ-C–H Functionalization of Alcohols via Olefin-Tethered Radical Relay. *JACS Au*. **2025,** DOI**:**10.1021/jacsau.5c00909.
13. Stateman, L. M.; Nakafuku, K. M.; Nagib, D. A. Remote C–H Functionalization via Selective Hydrogen Atom Transfer. *Synthesis* **2018**, *50* (08), 1569-1586.
14. Perrin, D. D.; Dempsey, B.; Serjeant, E. P. pKa Prediction for Organic Acids and Bases; Chapman & Hall: London, 1981.
15. Ho, J. Predicting pKa in Implicit Solvents: Current Status and Future Directions. *Aust. J. Chem.* **2014**, *67* (10).
16. Jensen, F. Introduction to Computational Chemistry, 2nd ed.; Wiley: Chichester, 2007.
17. Entgelmeier, L. M.; Mori, S.; Sendo, S.; Yamaguchi, R.; Suzuki, R.; Yanai, T.; Garcia Mancheno, O.; Ohmatsu, K.; Ooi, T. Zwitterionic Acridinium Amidate: A Nitrogen-Centered Radical Catalyst for Photoinduced Direct Hydrogen Atom Transfer. *Angew Chem Int Ed* **2024**, *63* (43), e202404890.
18. Flores-Holguín, N.; Frau, J.; Glossman-Mitnik, D. Computational Study of the Chemical Reactivity and Bioactivity Rates of Marine Peptides Hemiasterlin and Its A and B Derivatives Used in the Cancer Treatment through Conceptual Density Functional Theory. *Comput. Mol. Biosci* **2019**, *09* (04), 95-107.
19. Casasnovas, R.; Ortega-Castro, J.; Frau, J.; Donoso, J.; Muñoz, F. Theoretical pKa Calculations With Continuum Model Solvents, Alternative Protocols to Thermodynamic Cycles. *Int. J. Quantum Chem* **2014**, *114* (20), 1350-1363.
20. Sheldrick, G. M. SHELXT – Integrated space-group and crystal-structure determination. *Acta Cryst.* **2015**. *A71*, 3-8.
21. Sheldrick, G.M. Crystal Structure Refinement with SHELXL, *Acta Cryst.* **2015**. *C71*, 3-8.

10. NMR spectra

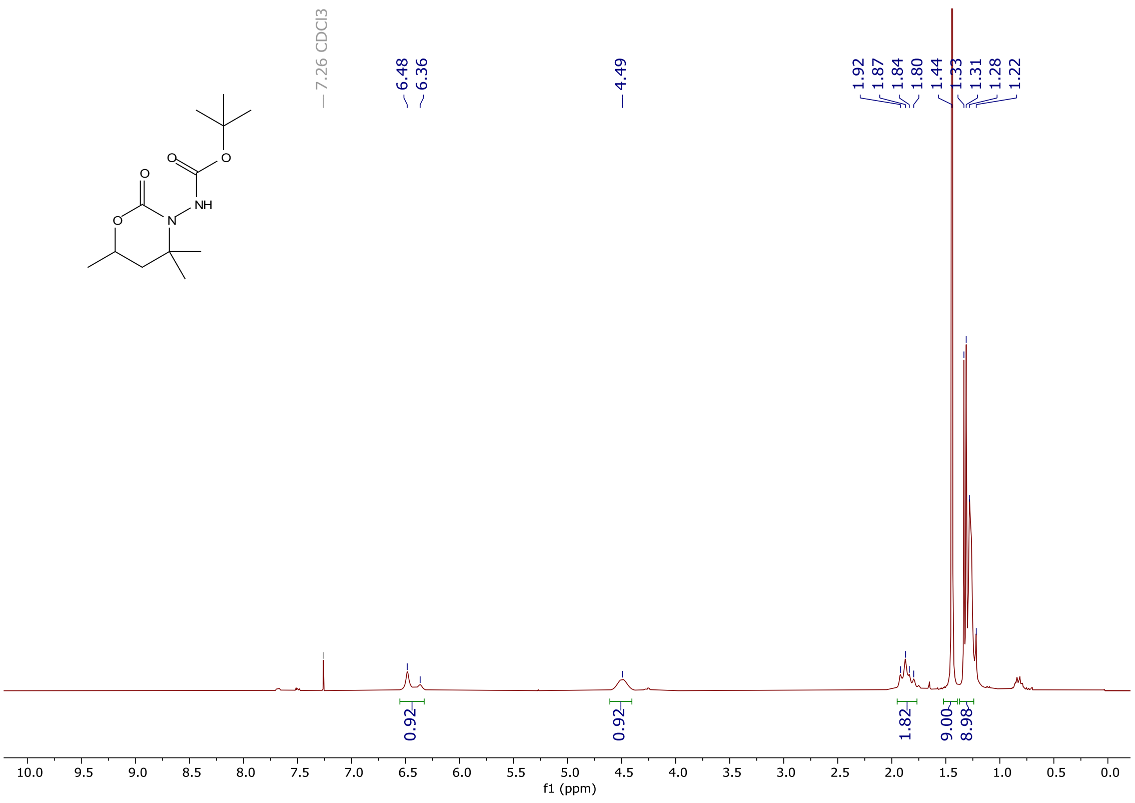

^{1}H NMR Spectrum of **2a** (300MHz, $CDCl_3$)

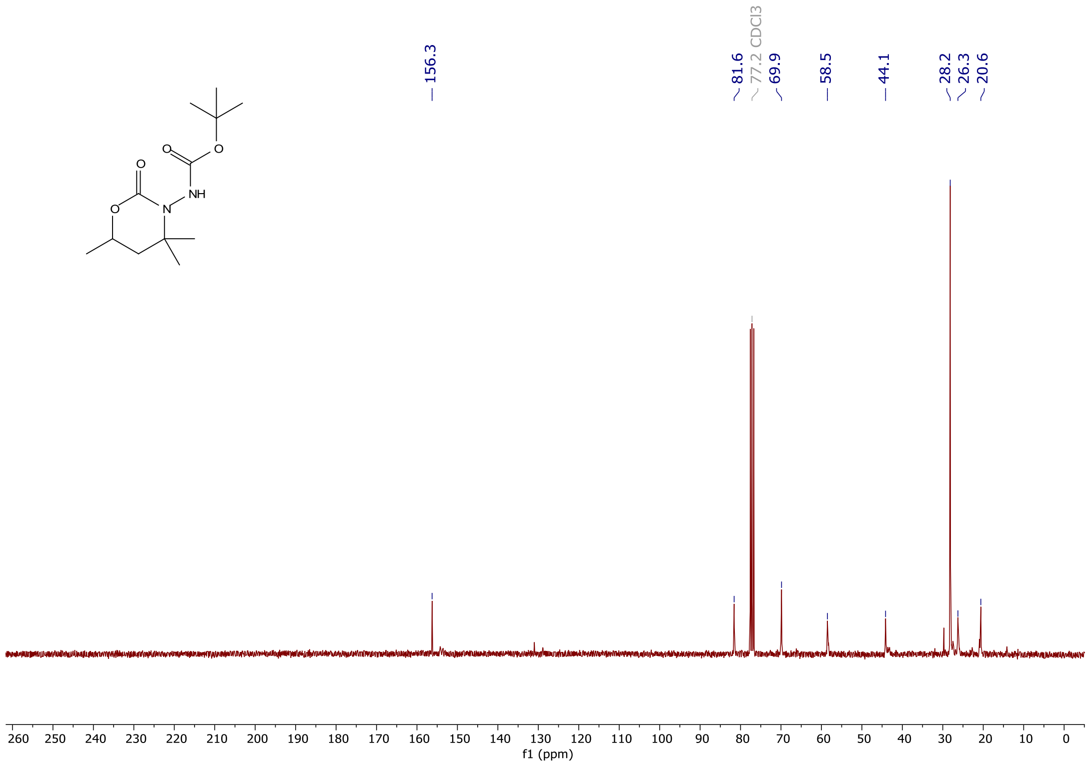

^{13}C NMR Spectrum of **2a (**101MHz, $CDCl_3$)

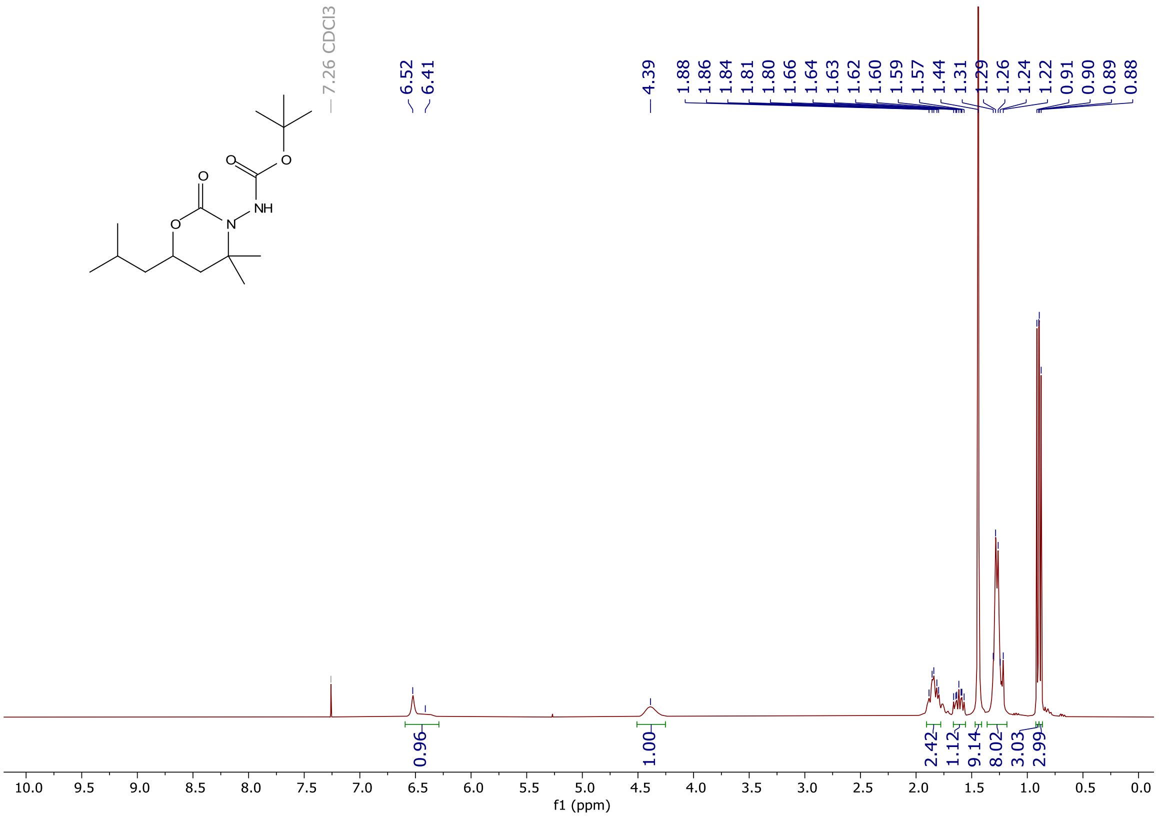

^{1}H NMR Spectrum of **2b** (300MHz, $CDCl_3$)

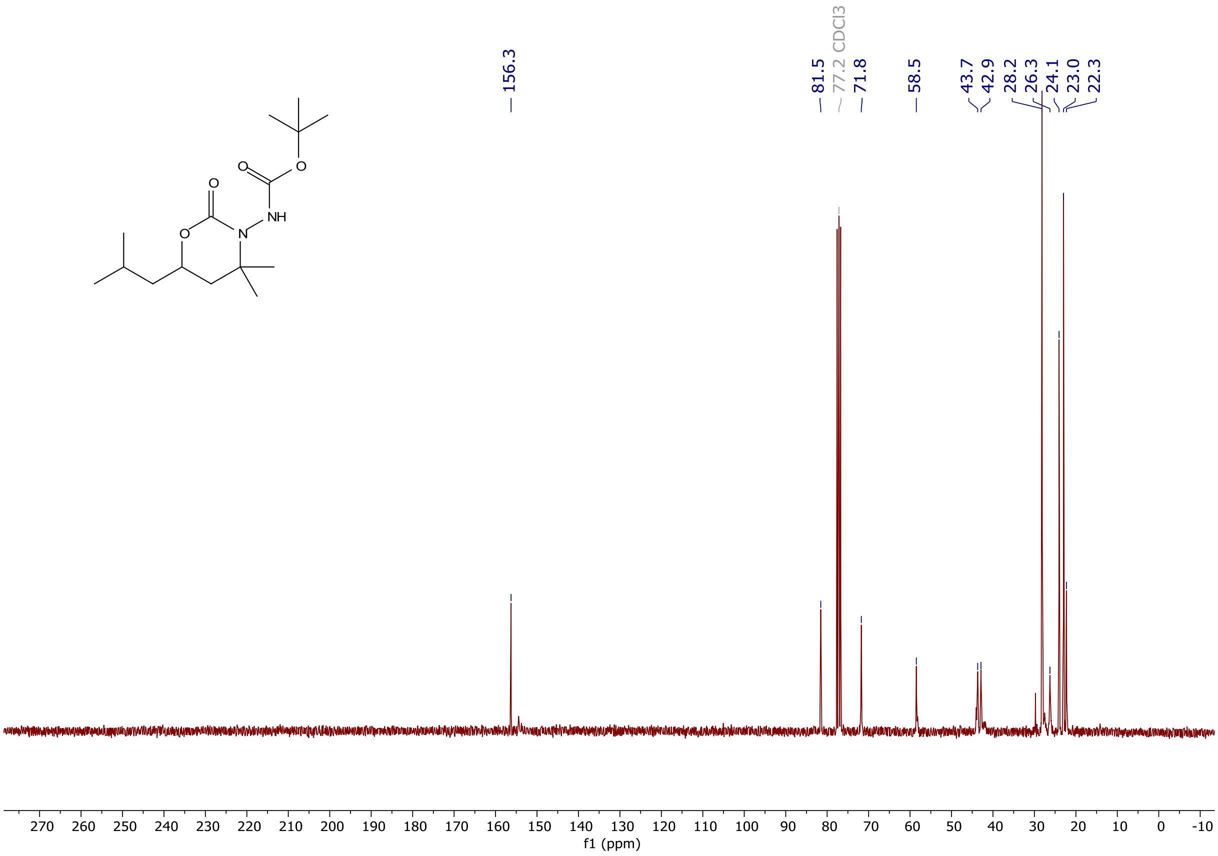

^{13}C NMR Spectrum of **2b (**101MHz, $CDCl_3$)

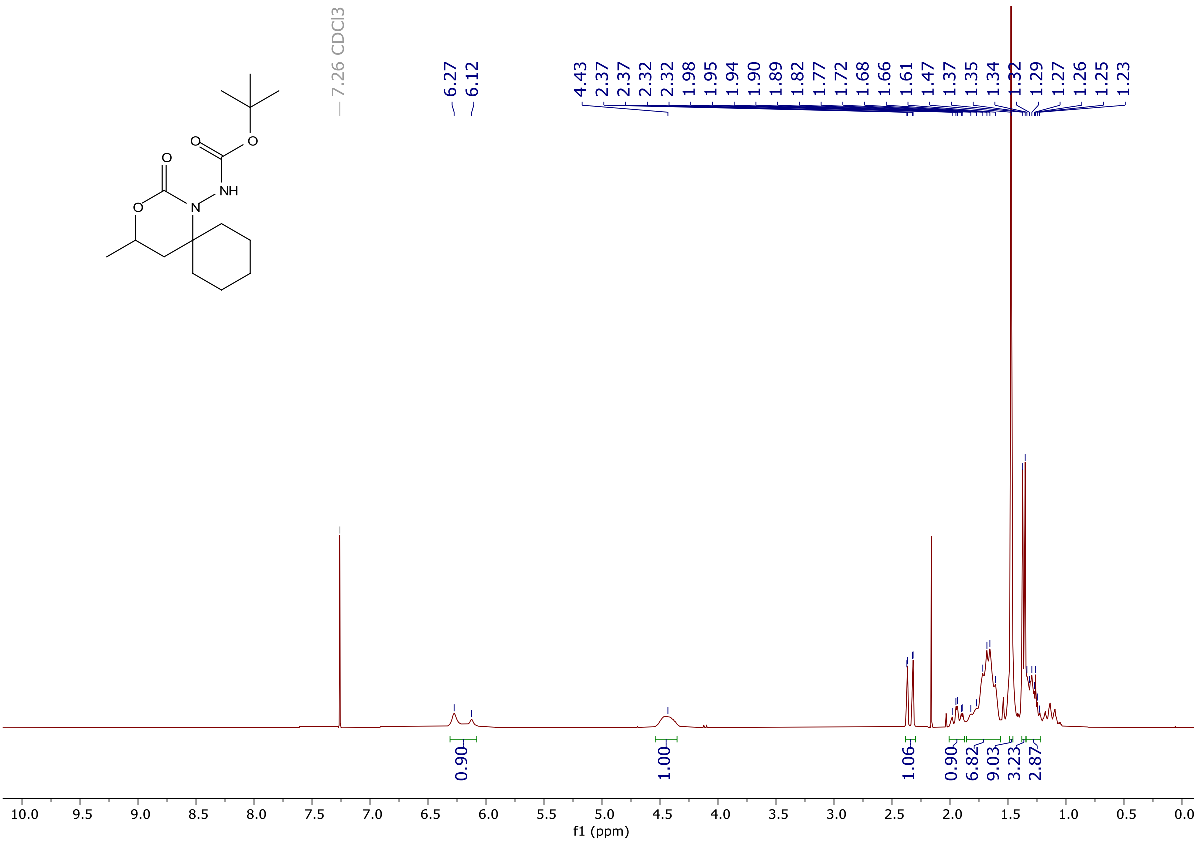

^{1}H NMR Spectrum of **2c** (300MHz, $CDCl_3$)

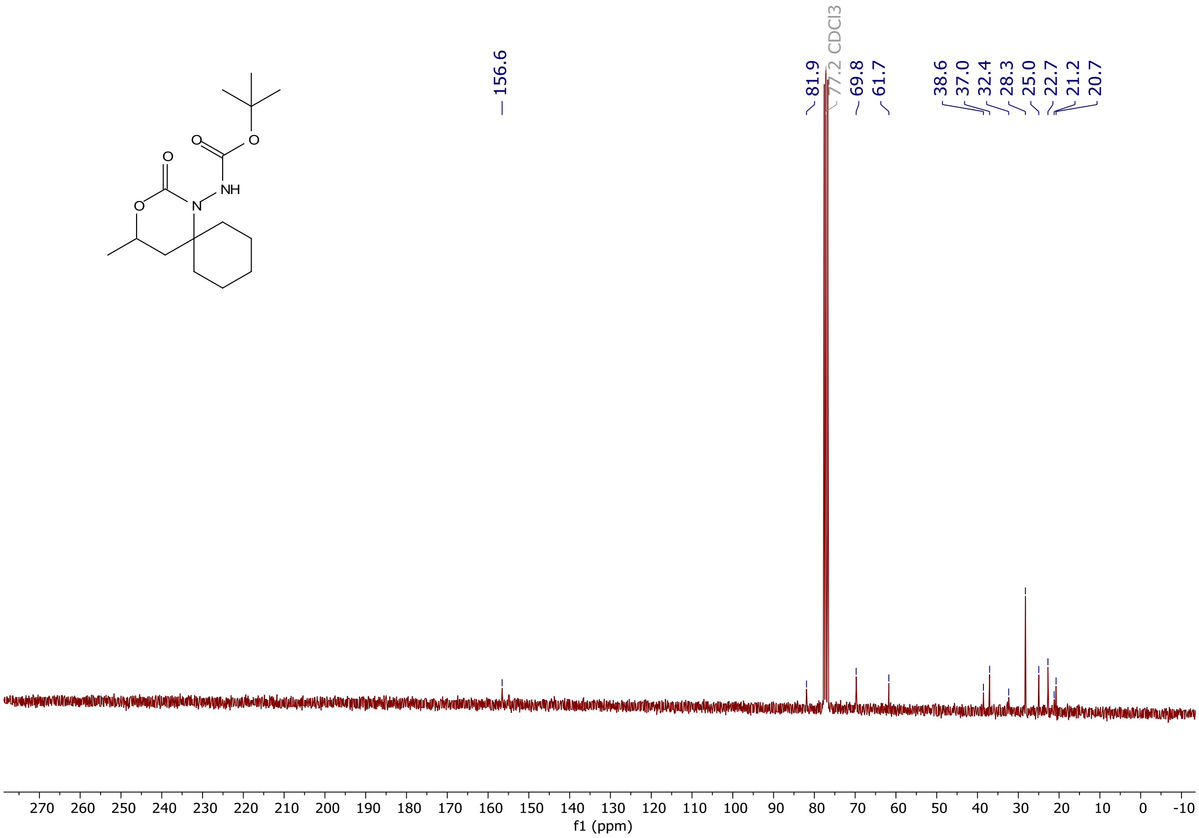

^{13}C NMR Spectrum of **2c (**101MHz, $CDCl_3$)

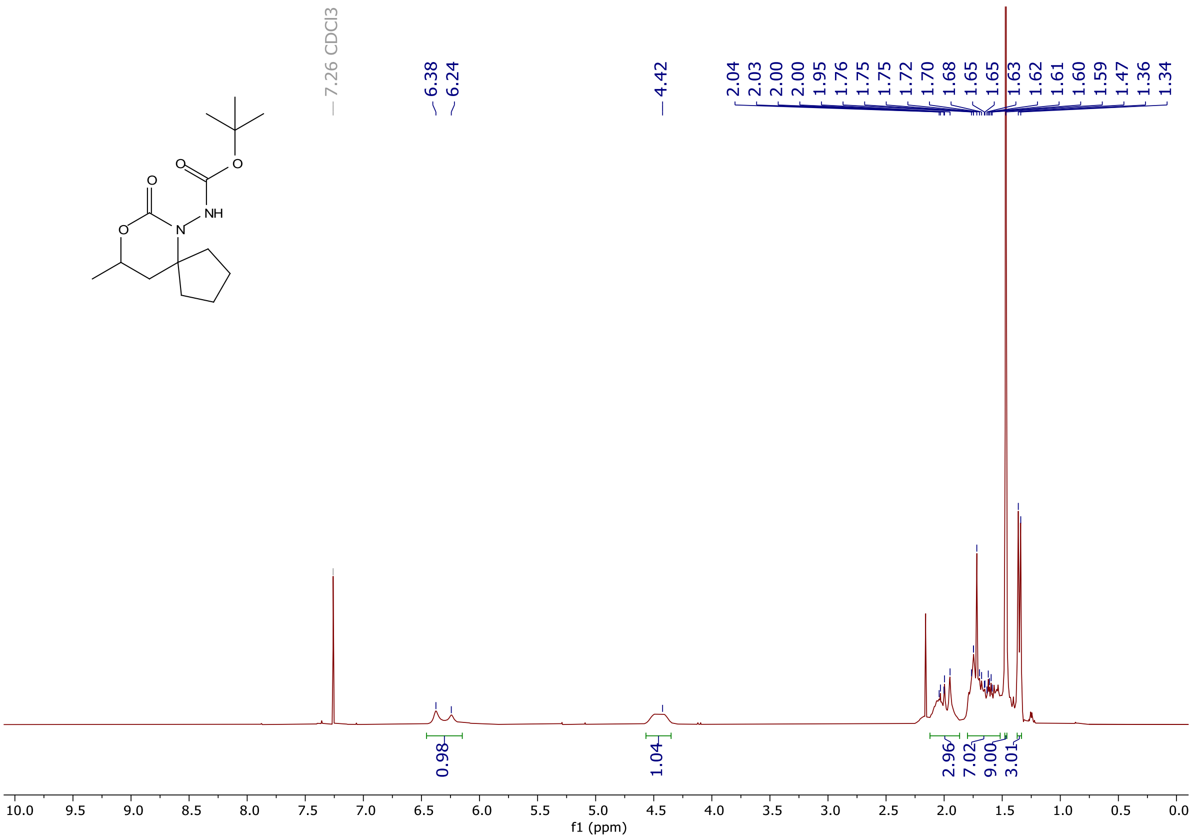

^{1}H NMR Spectrum of **2d** (300MHz, $CDCl_3$)

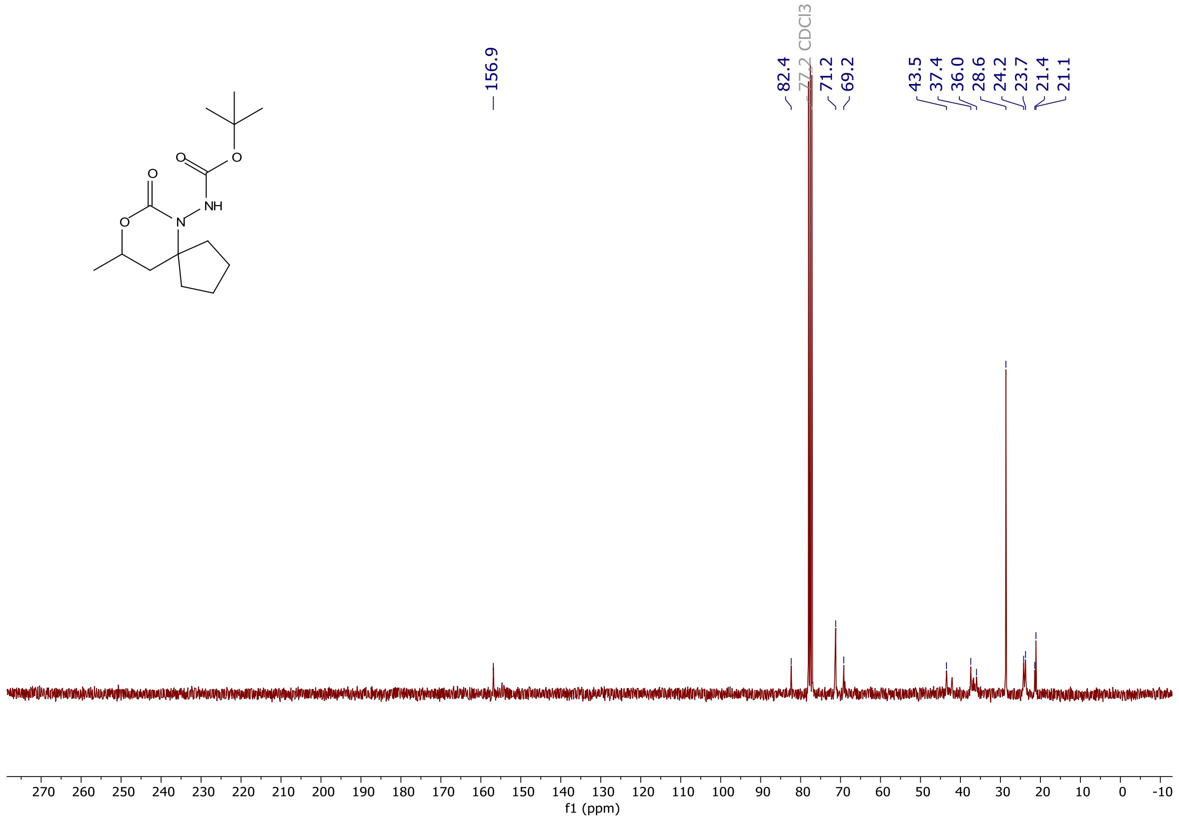

^{13}C NMR spectrum of **2d** (101MHz, $CDCl_3$)

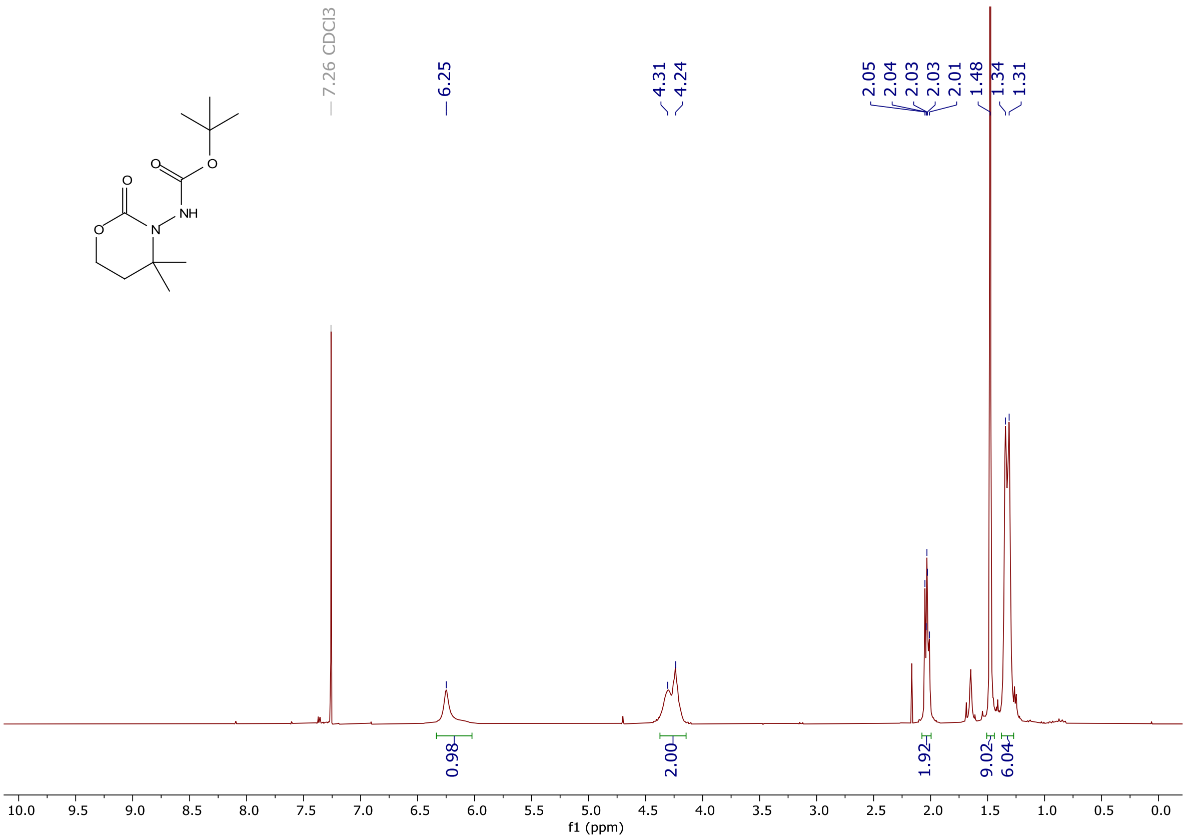

^{1}H NMR Spectrum of **2e** (300MHz, $CDCl_3$)

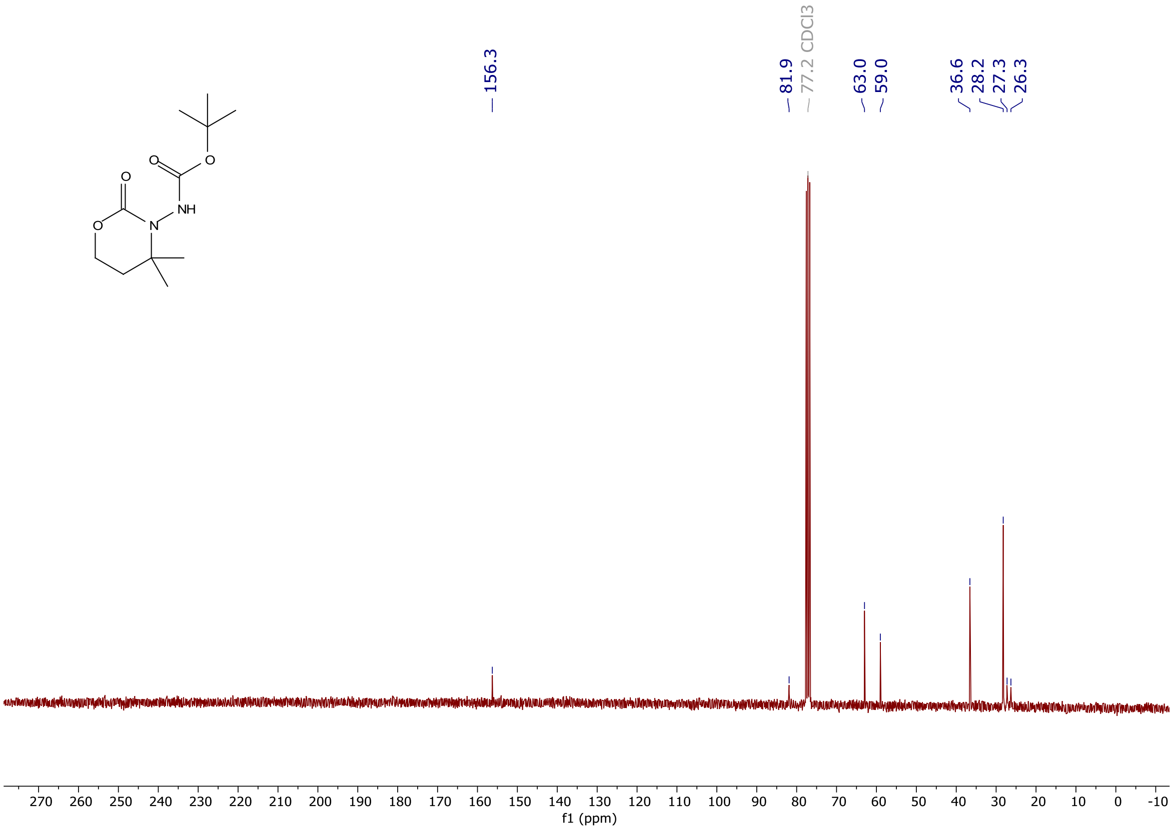

^{13}C NMR Spectrum of **2e** (101MHz, $CDCl_3$)

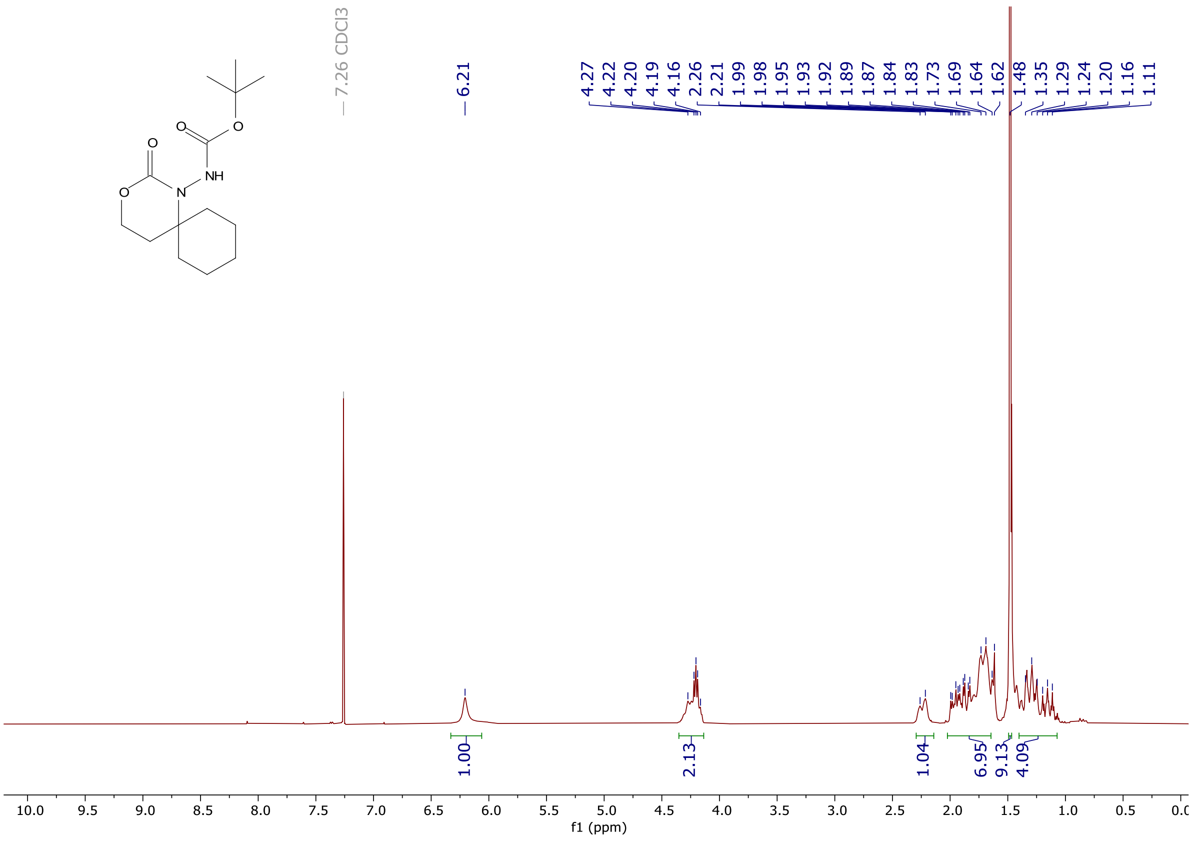

^{1}H NMR Spectrum of **2f** (300MHz, $CDCl_3$)

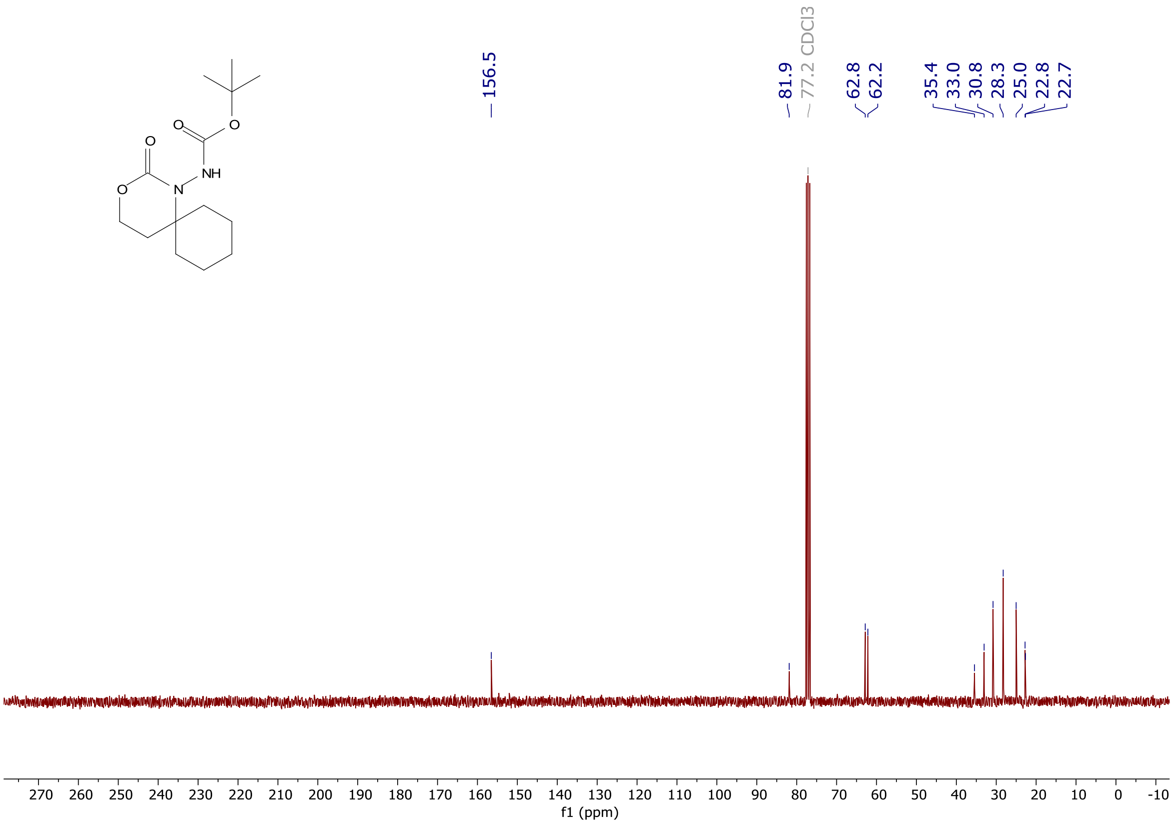

^{13}C NMR Spectrum of **2f (**101MHz, $CDCl_3$)

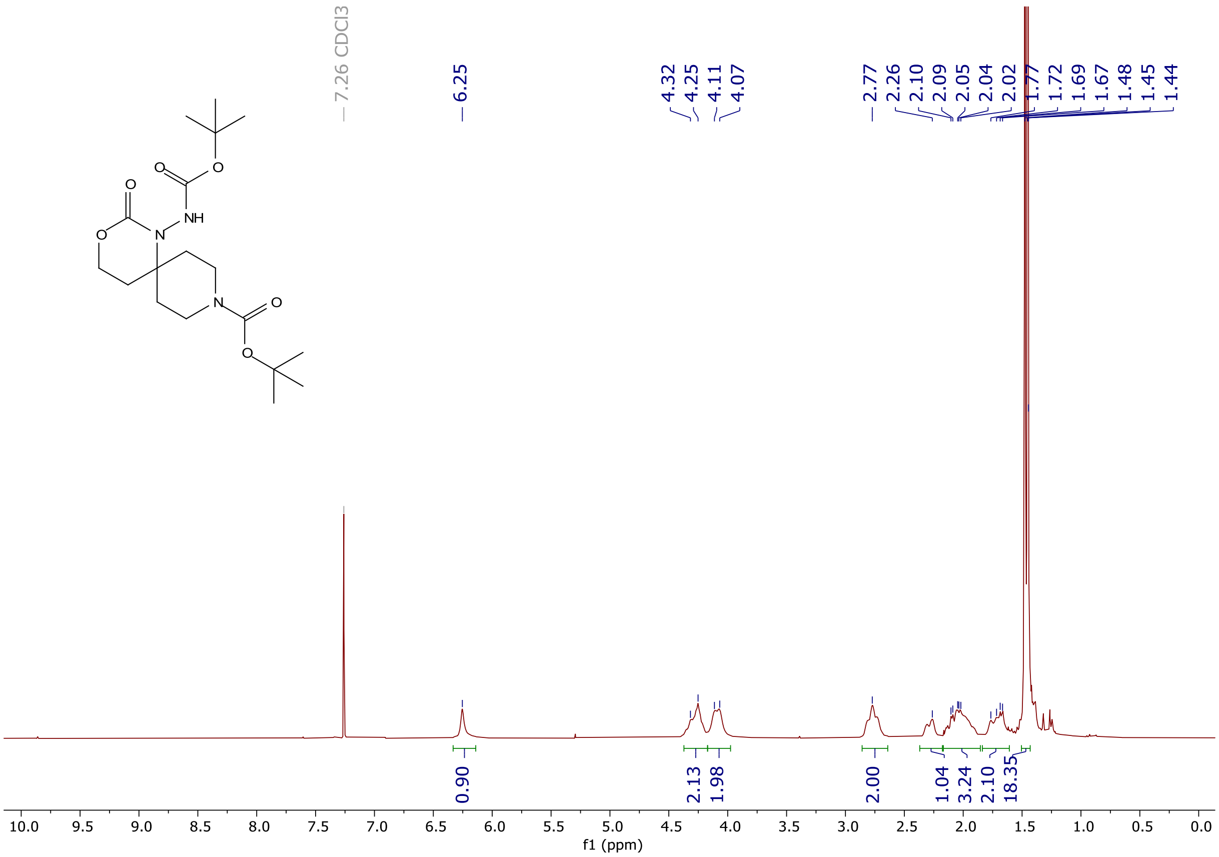

^{1}H NMR Spectrum of **2g** (300MHz, $CDCl_3$)

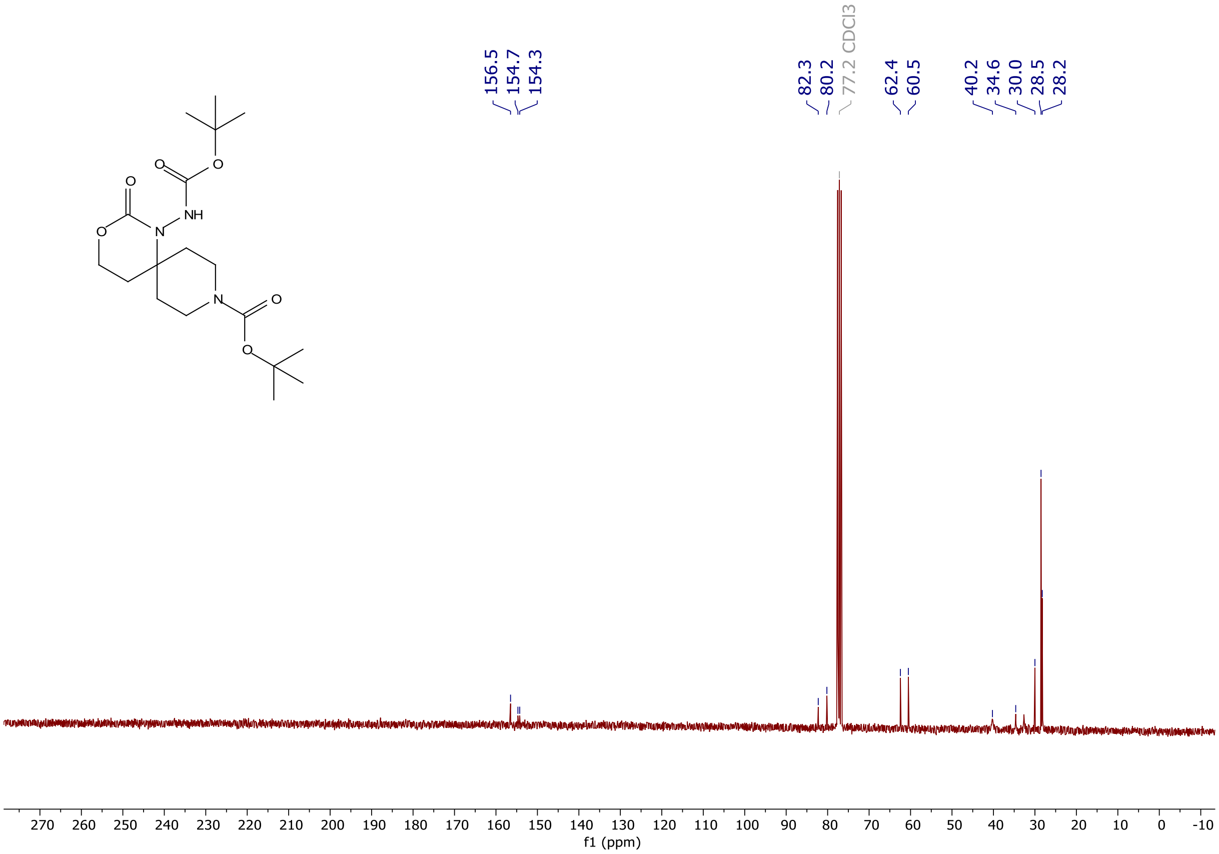

^{13}C NMR Spectrum of **2g** (101MHz, $CDCl_3$)

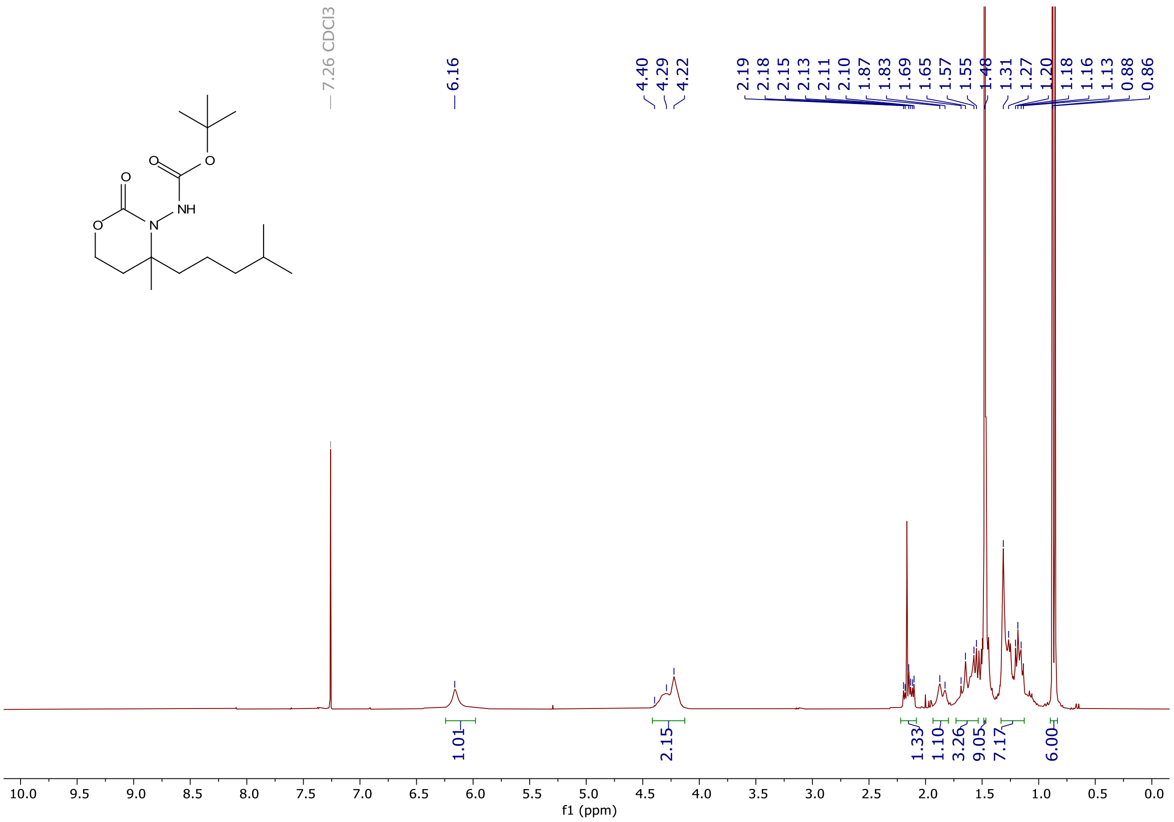

^{1}H NMR Spectrum of **2h** (300MHz, $CDCl_3$)

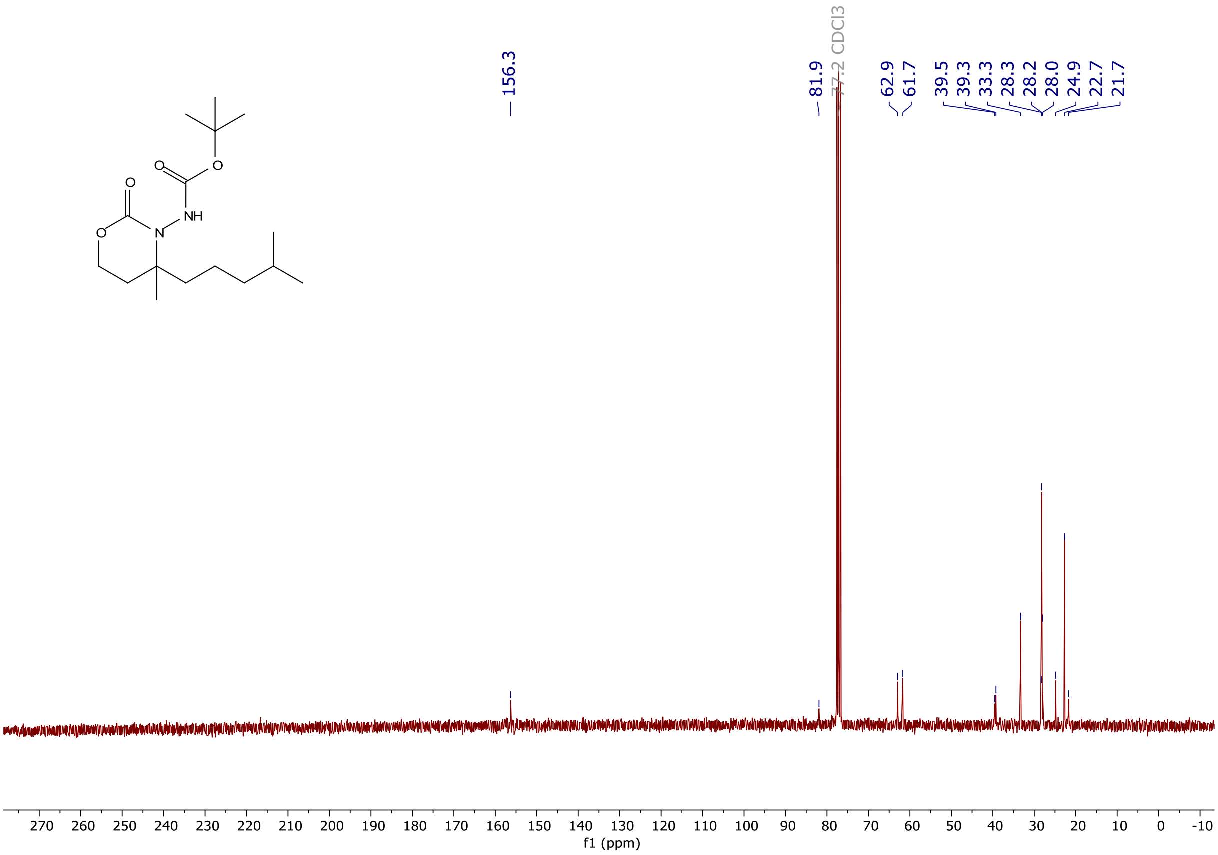

^{13}C NMR Spectrum of **2h (**101MHz, $CDCl_3$)

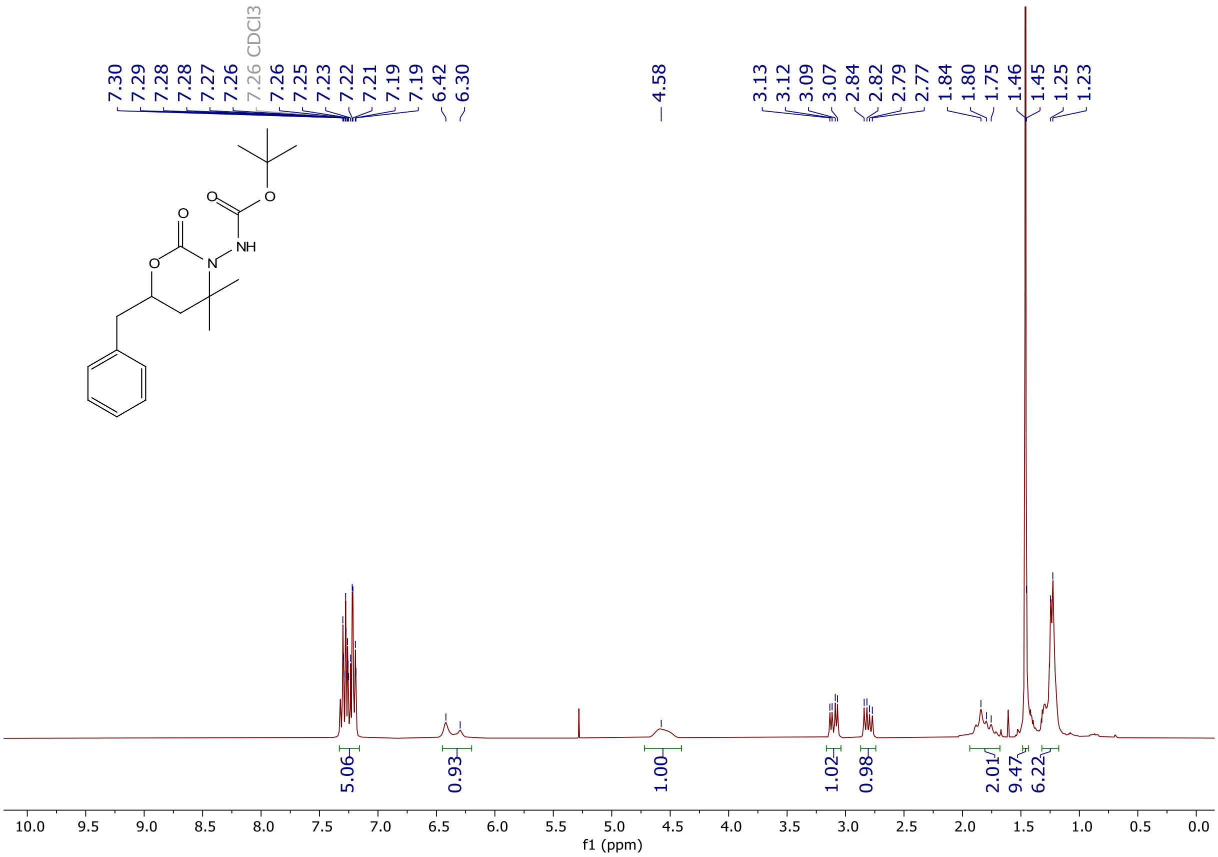

^{1}H NMR Spectrum of **2i** (300MHz, $CDCl_3$)

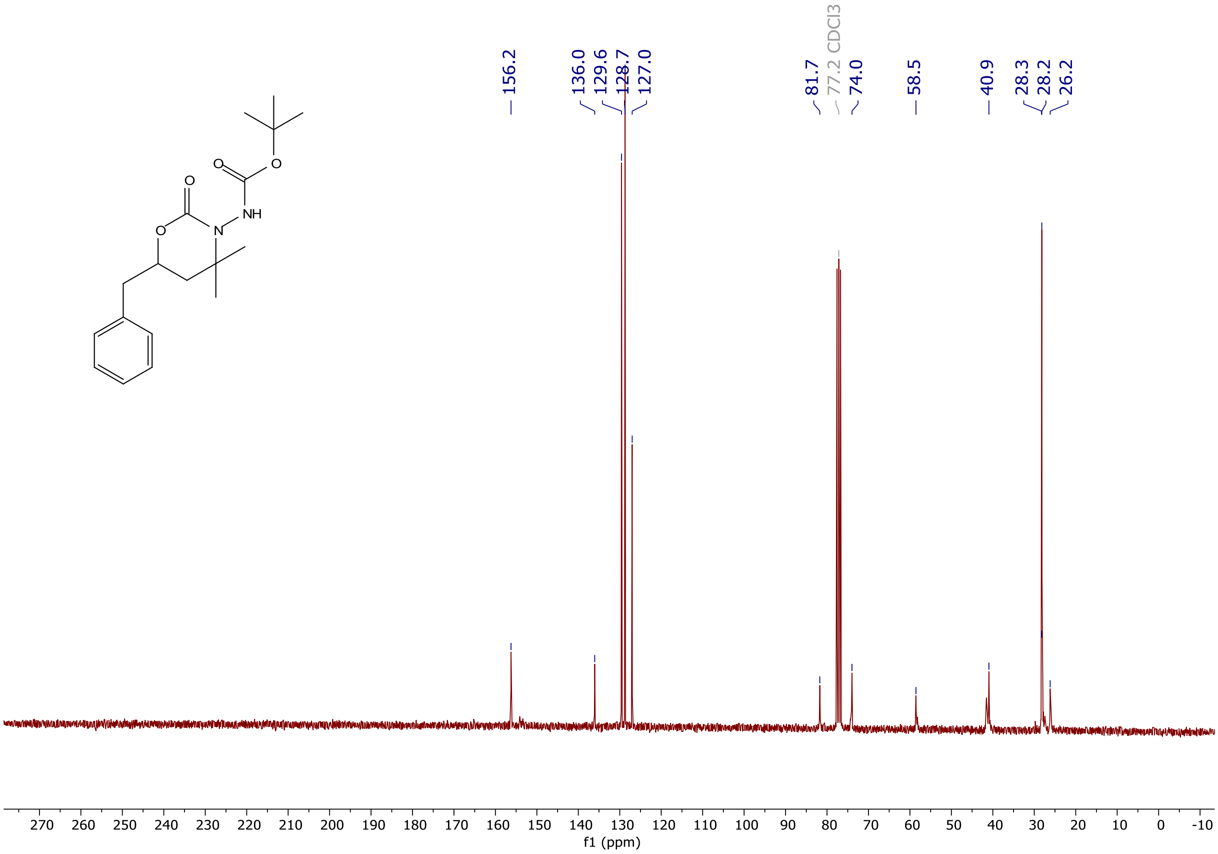

^{13}C NMR Spectrum of **2i** (101MHz, $CDCl_3$)

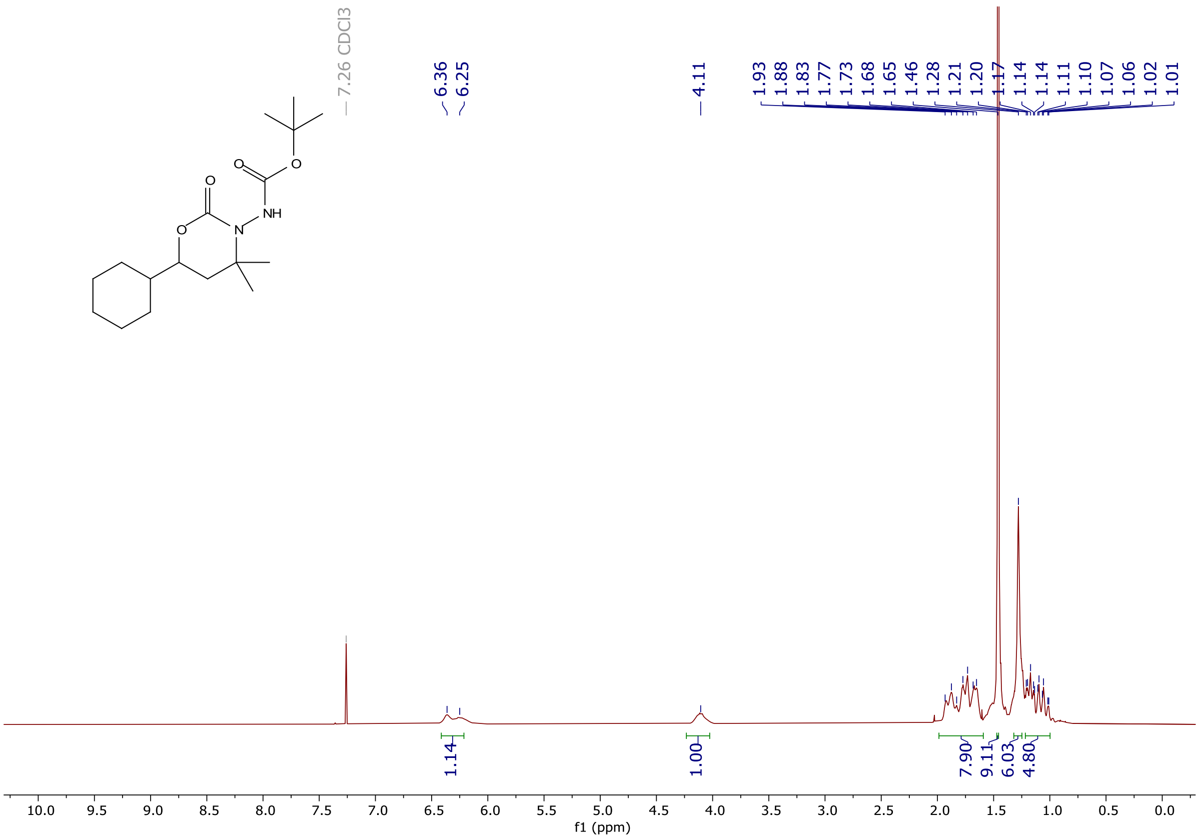

^{1}H NMR Spectrum of **2j** (300MHz, CDCl$_3$)

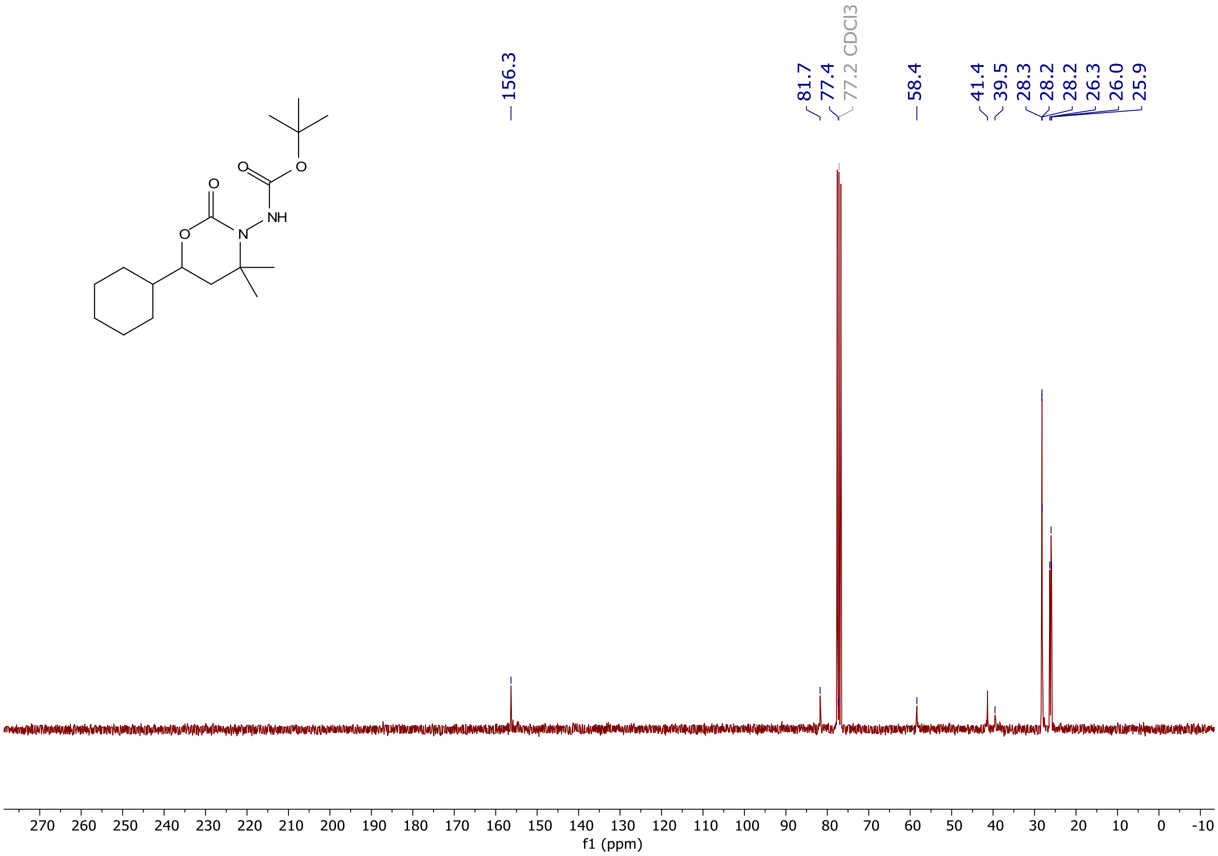

^{13}C NMR Spectrum of **2j** (101MHz, CDCl$_3$)

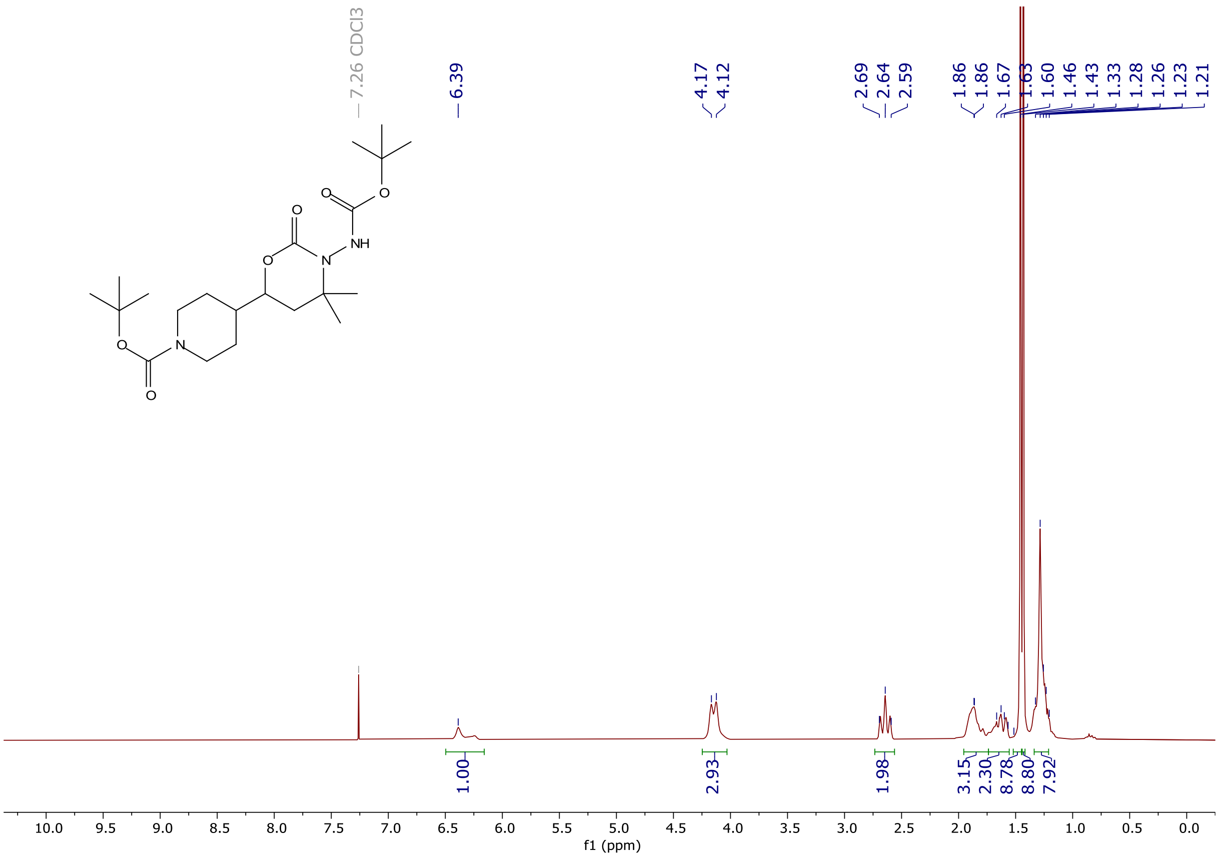

^{1}H NMR Spectrum of **2k** (300MHz, $CDCl_3$)

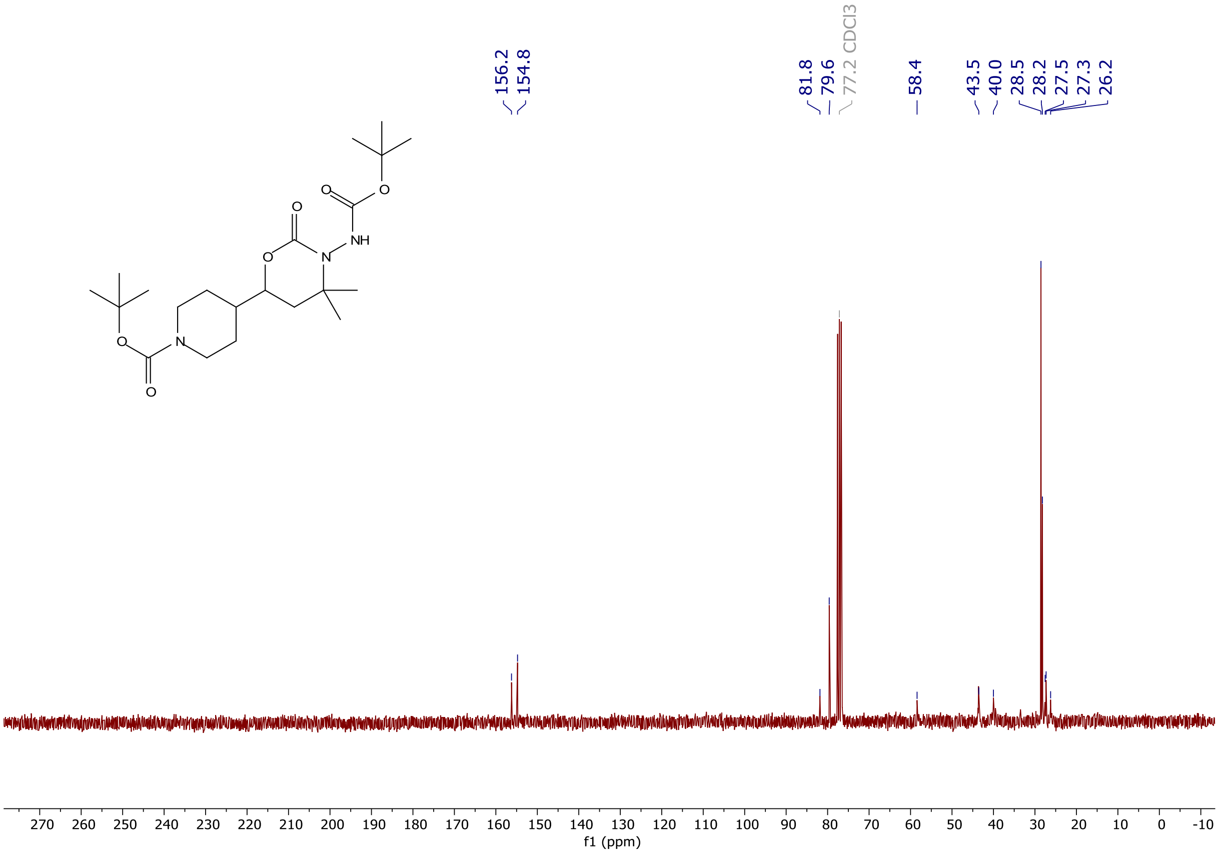

^{13}C NMR Spectrum of **2k (**101MHz, $CDCl_3$)

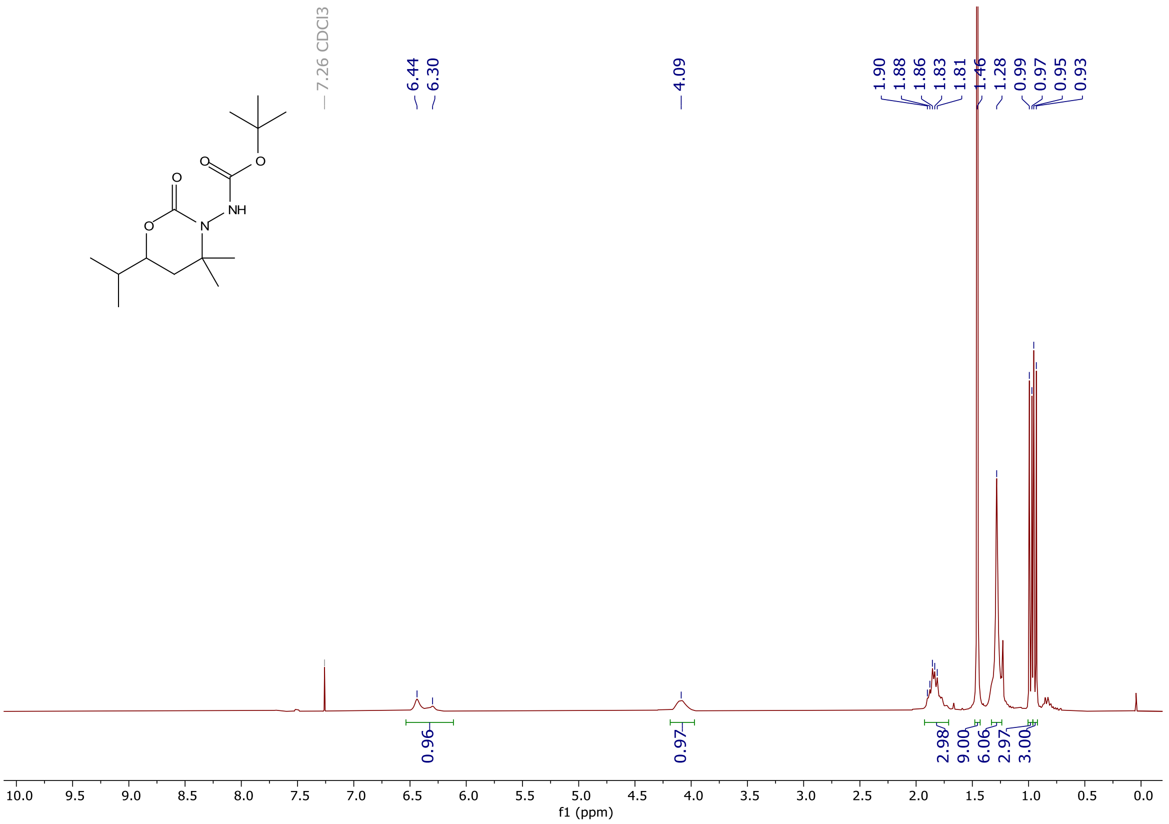

^{1}H NMR Spectrum of **2l** (300MHz, $CDCl_3$)

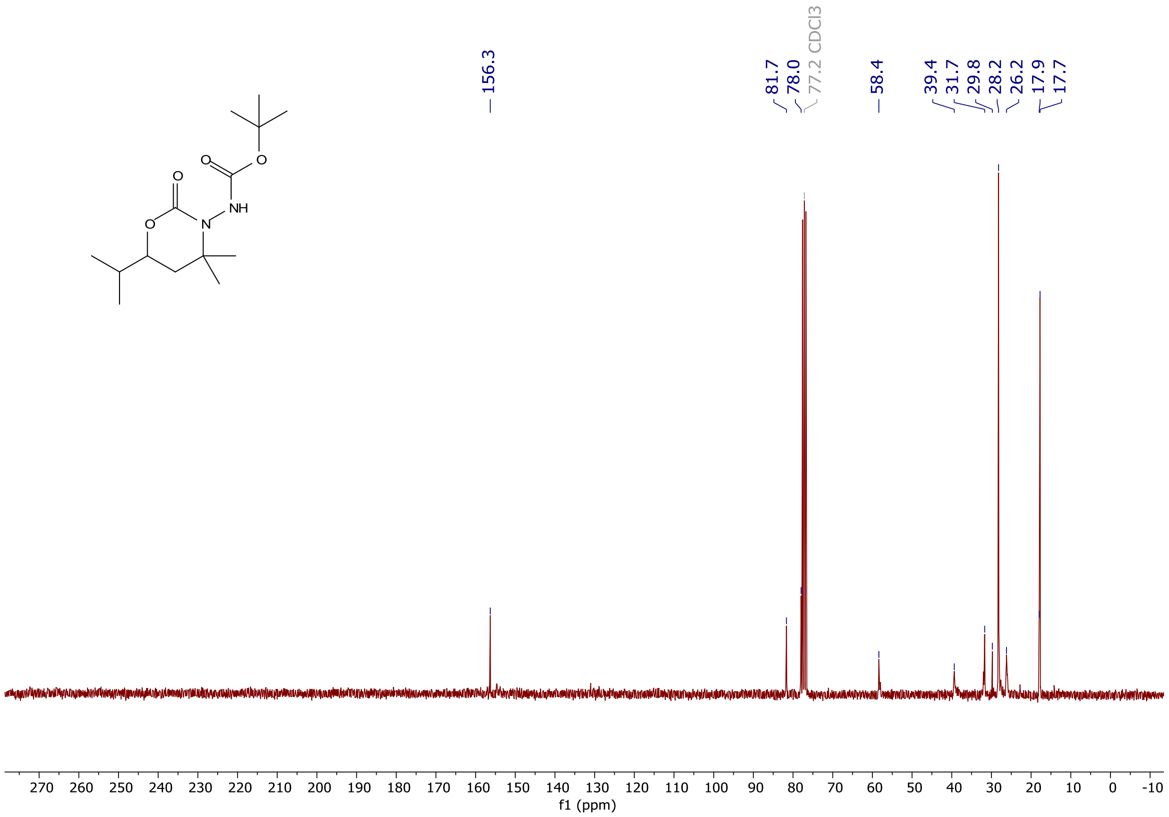

^{13}C NMR Spectrum of **2l (**101MHz, $CDCl_3$)

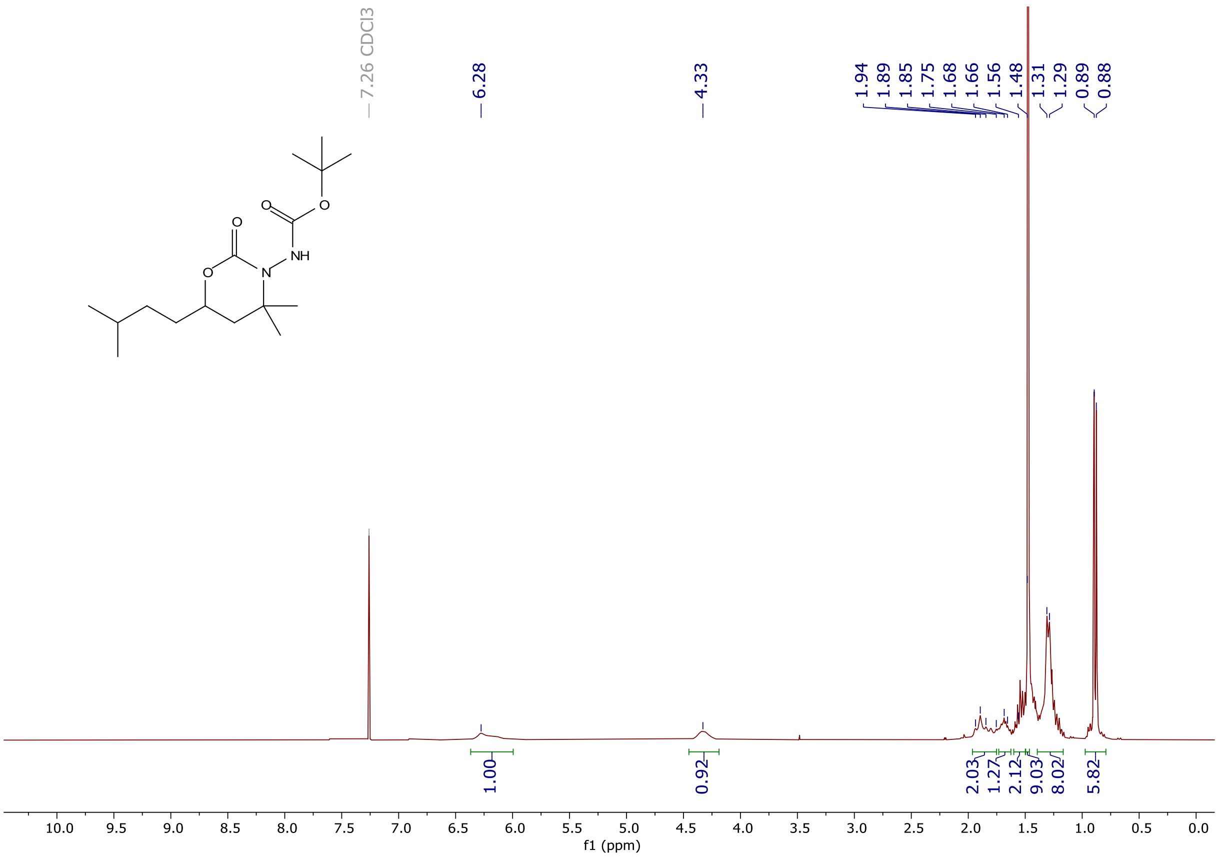

^{1}H NMR Spectrum of **2m** (300MHz, $CDCl_3$)

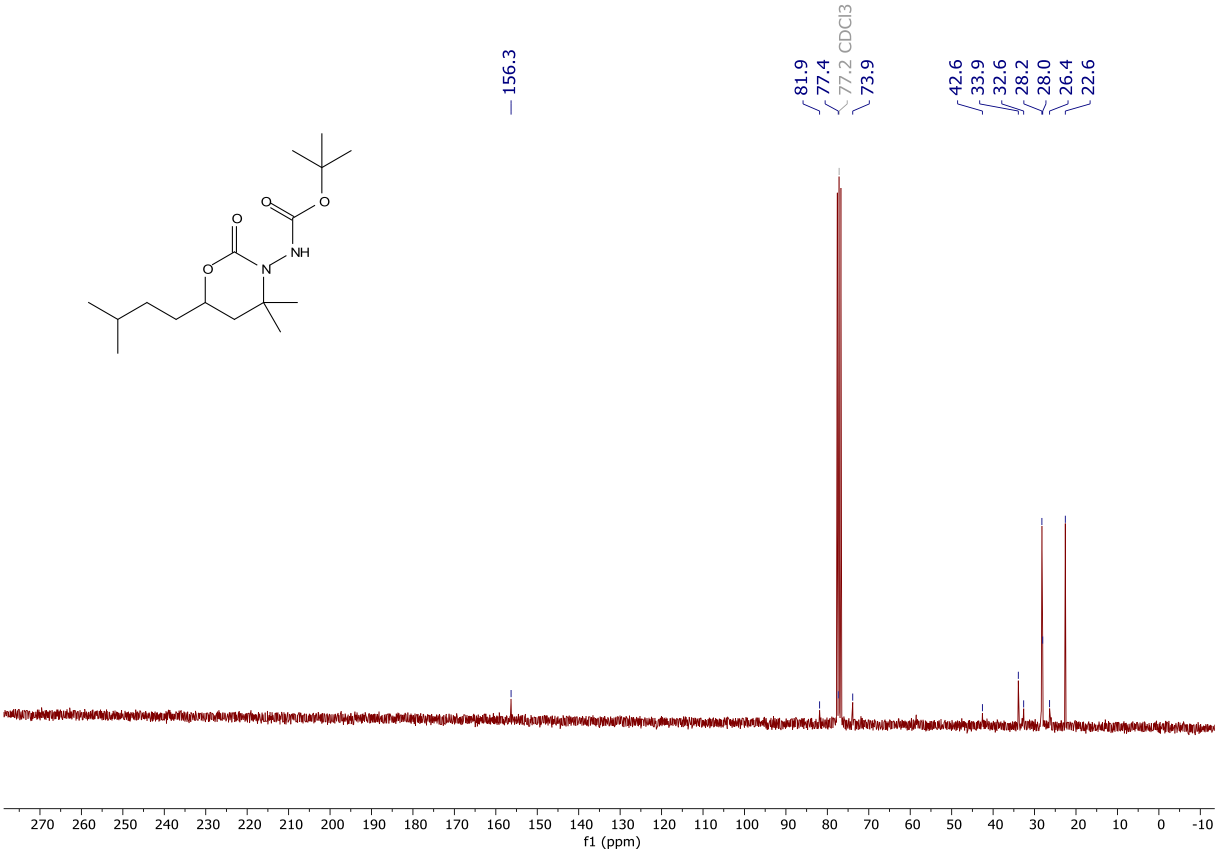

^{13}C NMR Spectrum of **2m (**101MHz, $CDCl_3$)

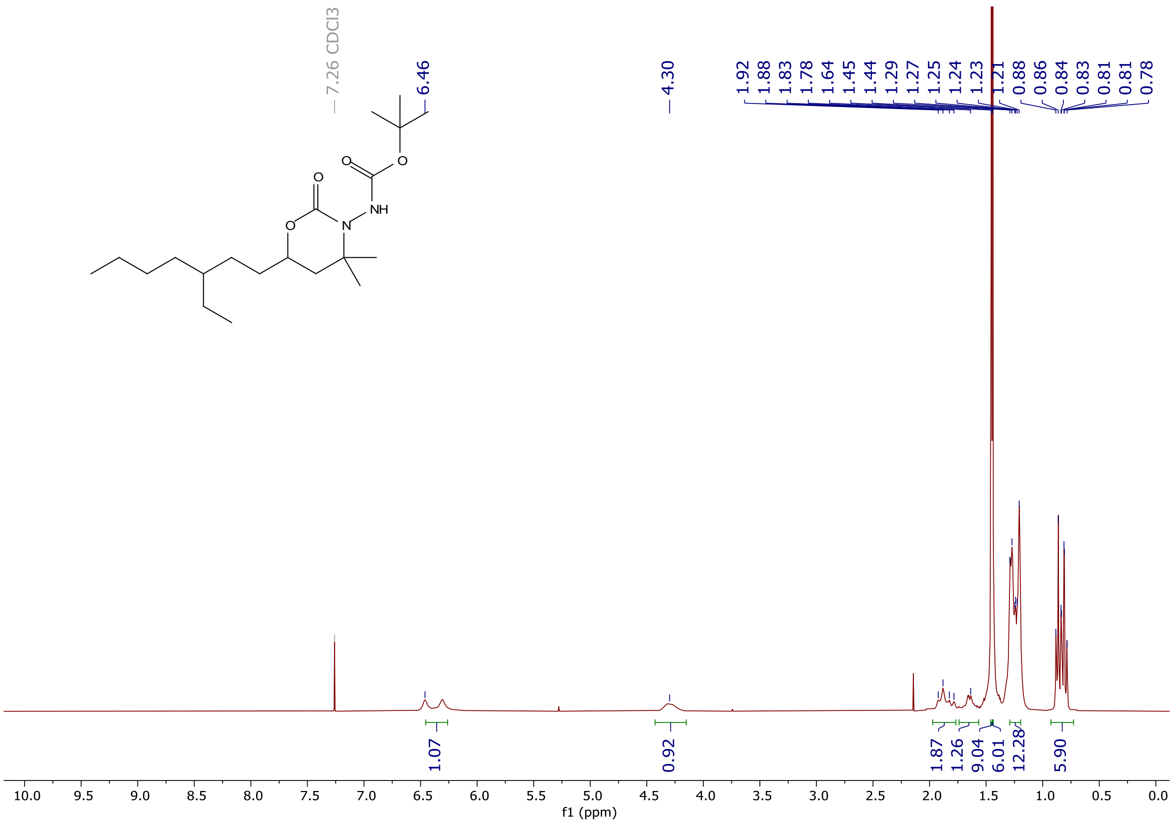

^{1}H NMR Spectrum of **2n** (300MHz, $CDCl_3$)

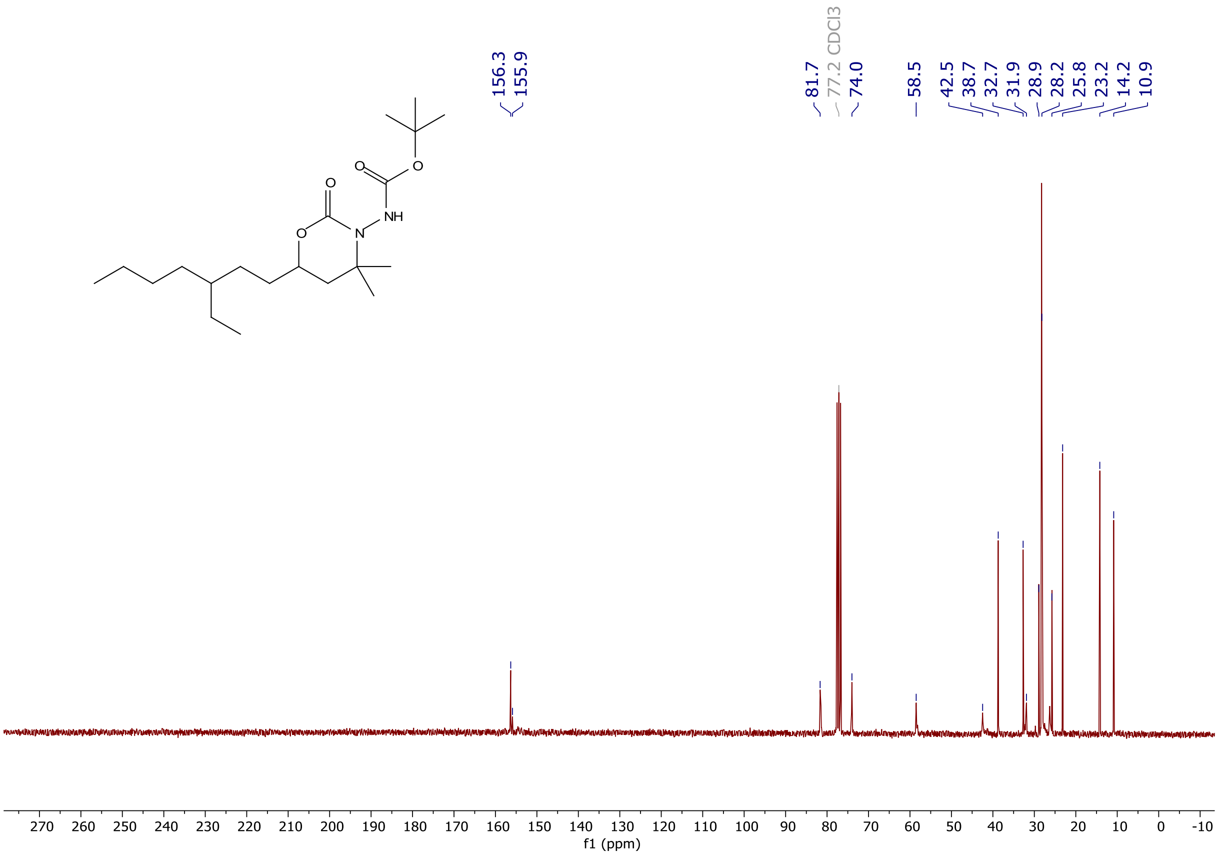

^{13}C NMR Spectrum of **2n (**101MHz, $CDCl_3$)

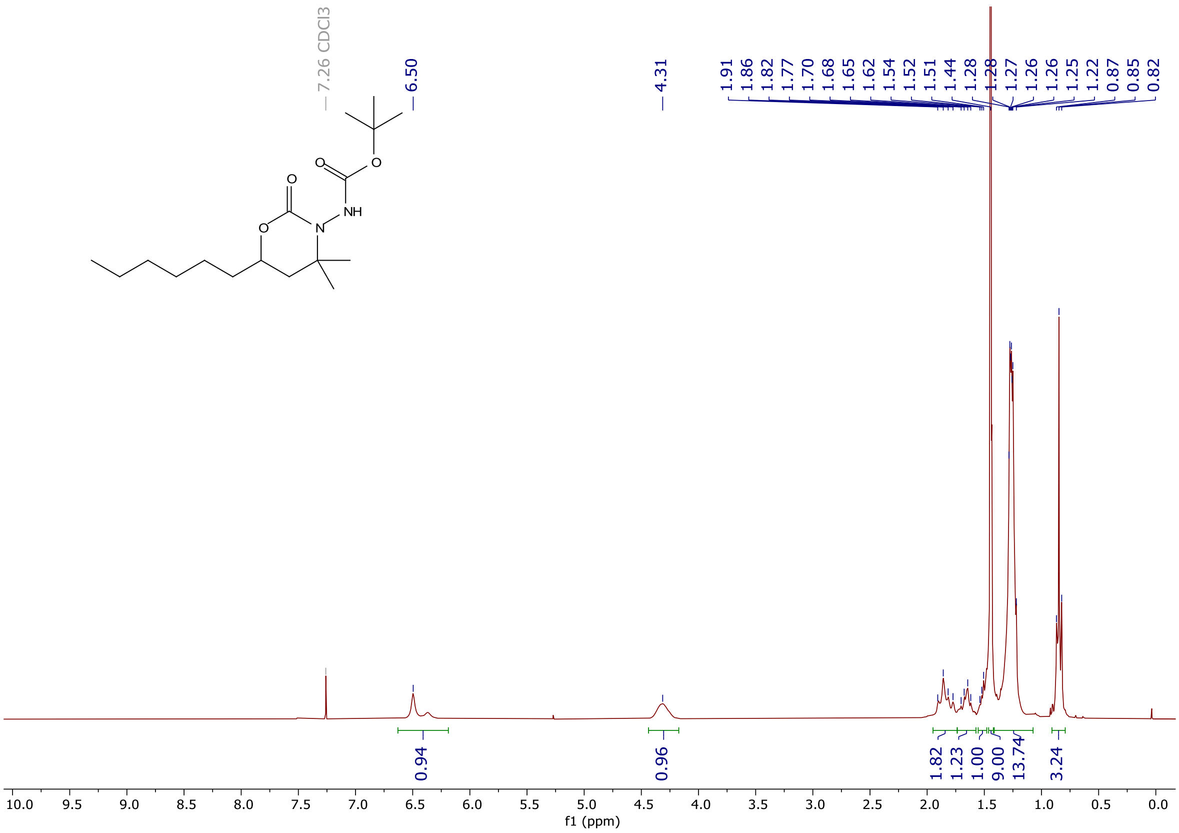

^{1}H NMR Spectrum of **2o** (300MHz, $CDCl_3$)

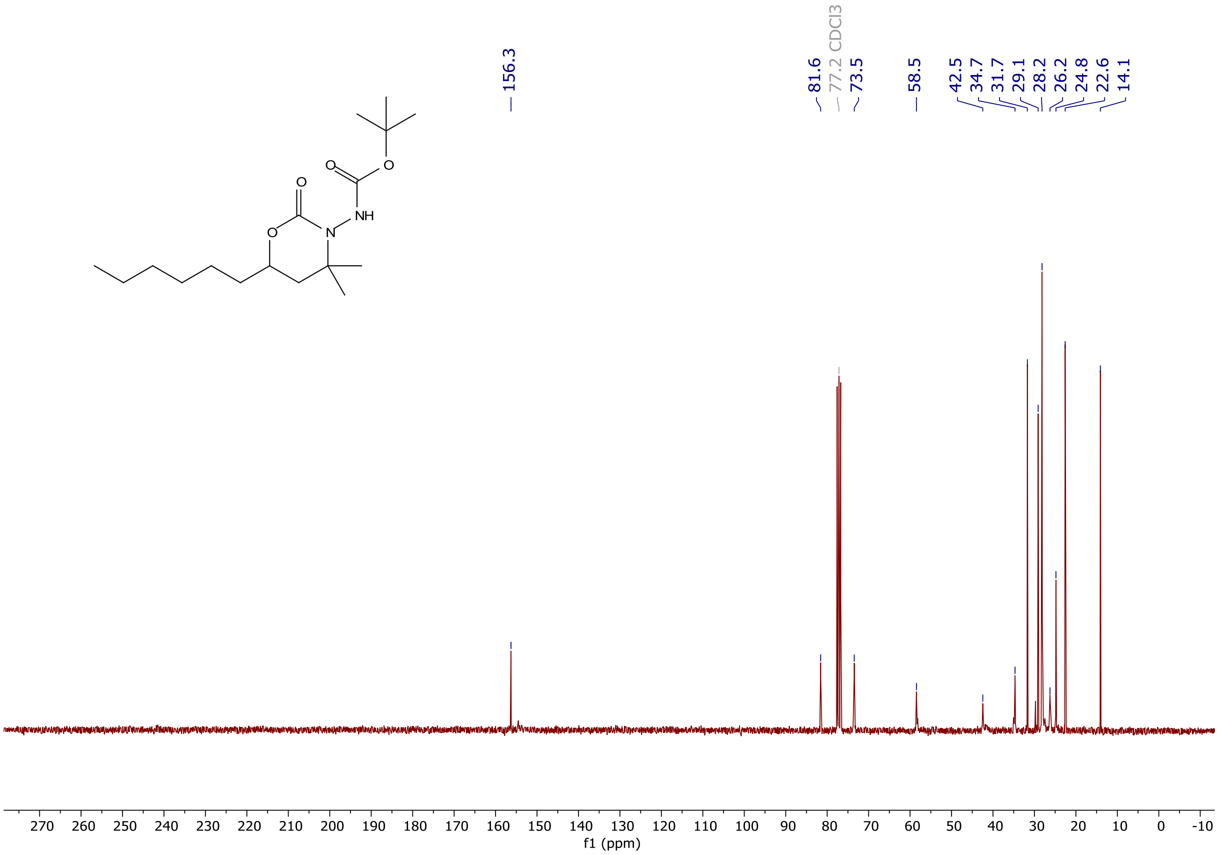

^{13}C NMR Spectrum of **2o** (101MHz, $CDCl_3$)

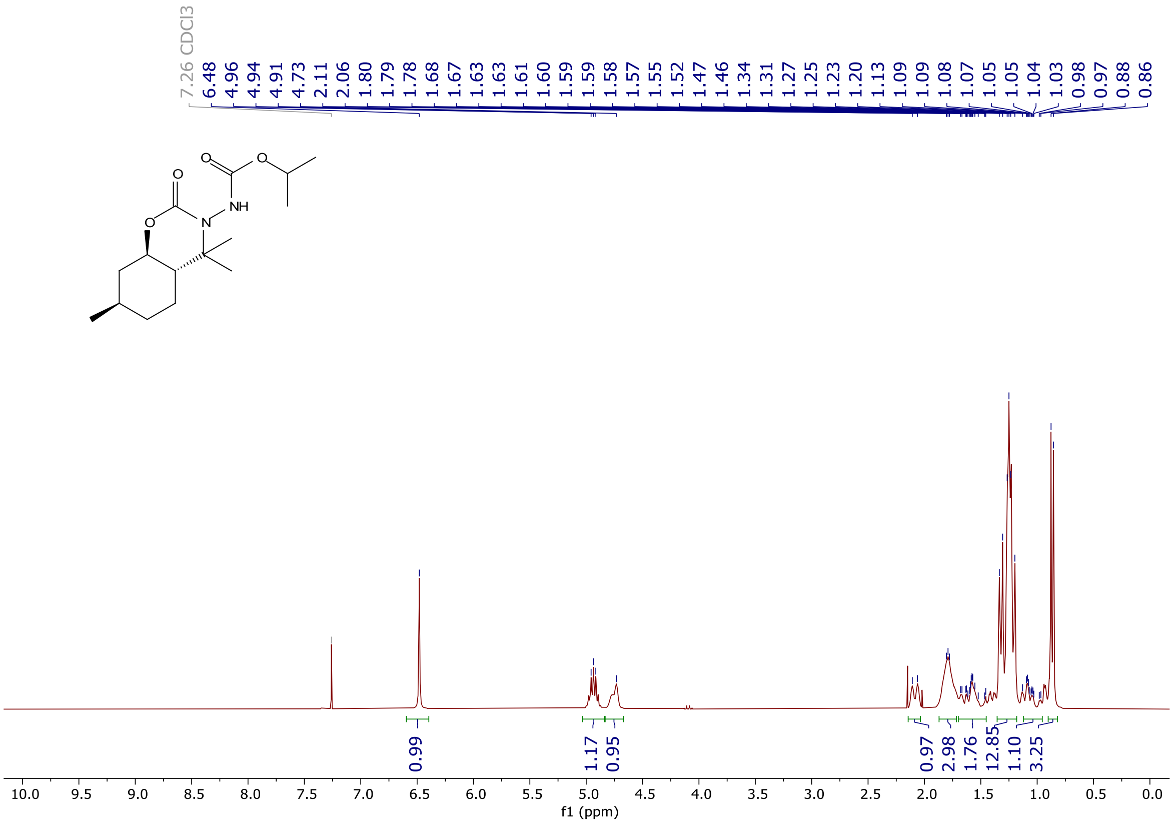

^{1}H NMR Spectrum of **2p** (300MHz, $CDCl_3$)

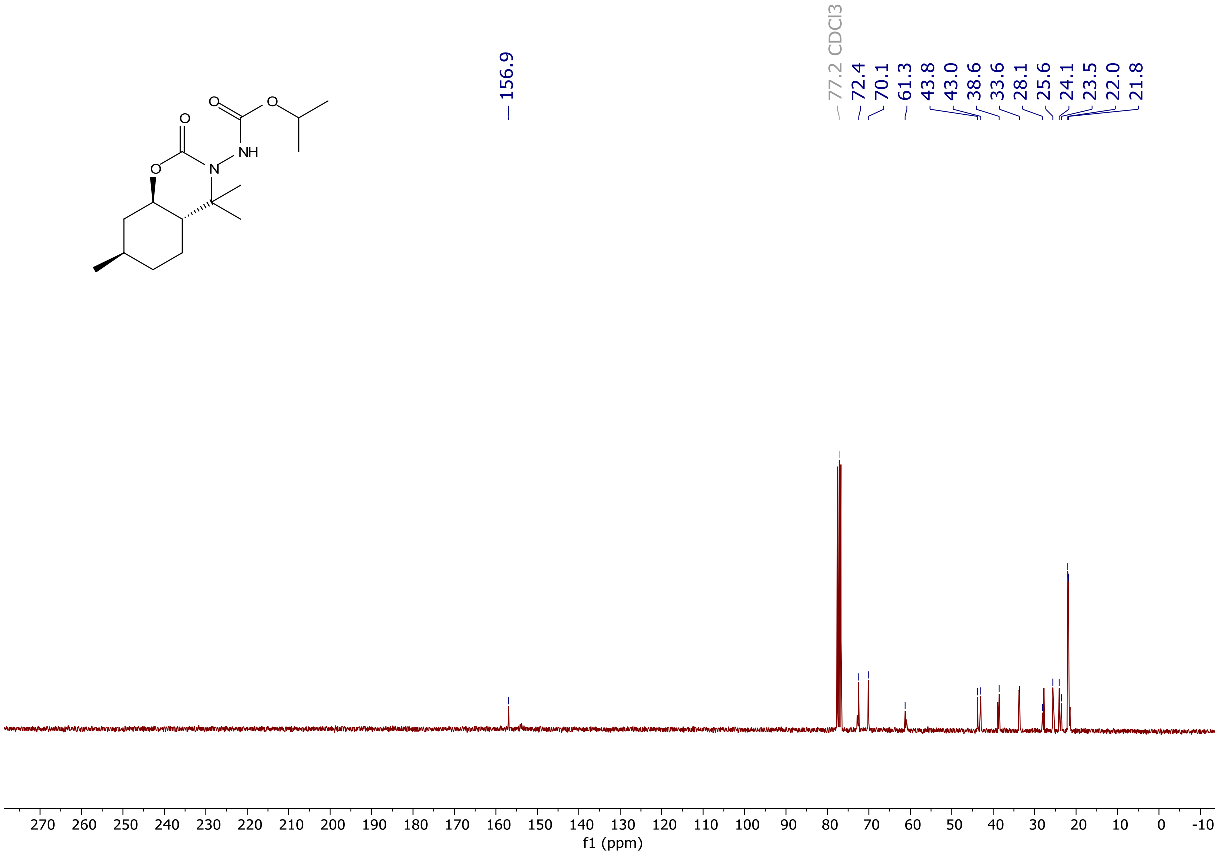

^{13}C NMR Spectrum of **2p (**101MHz, $CDCl_3$)

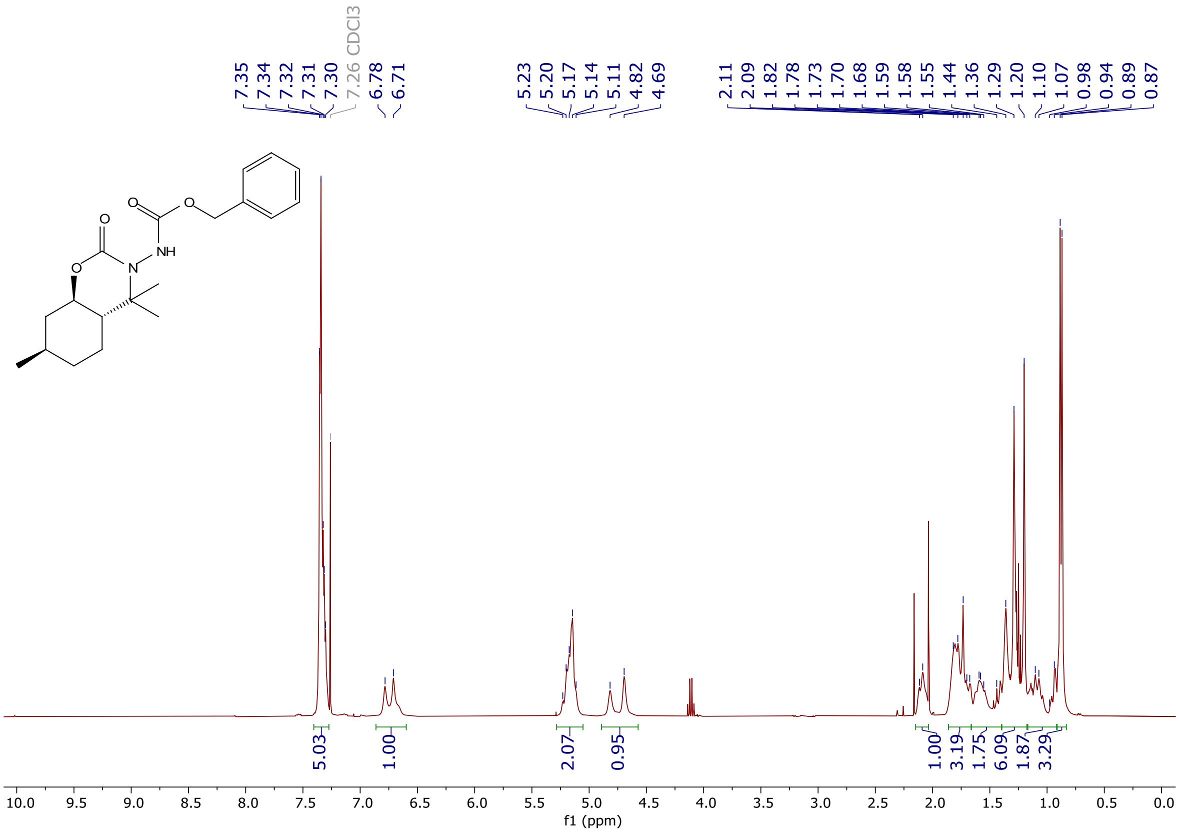

^{1}H NMR Spectrum of **2q** (300MHz, $CDCl_3$)

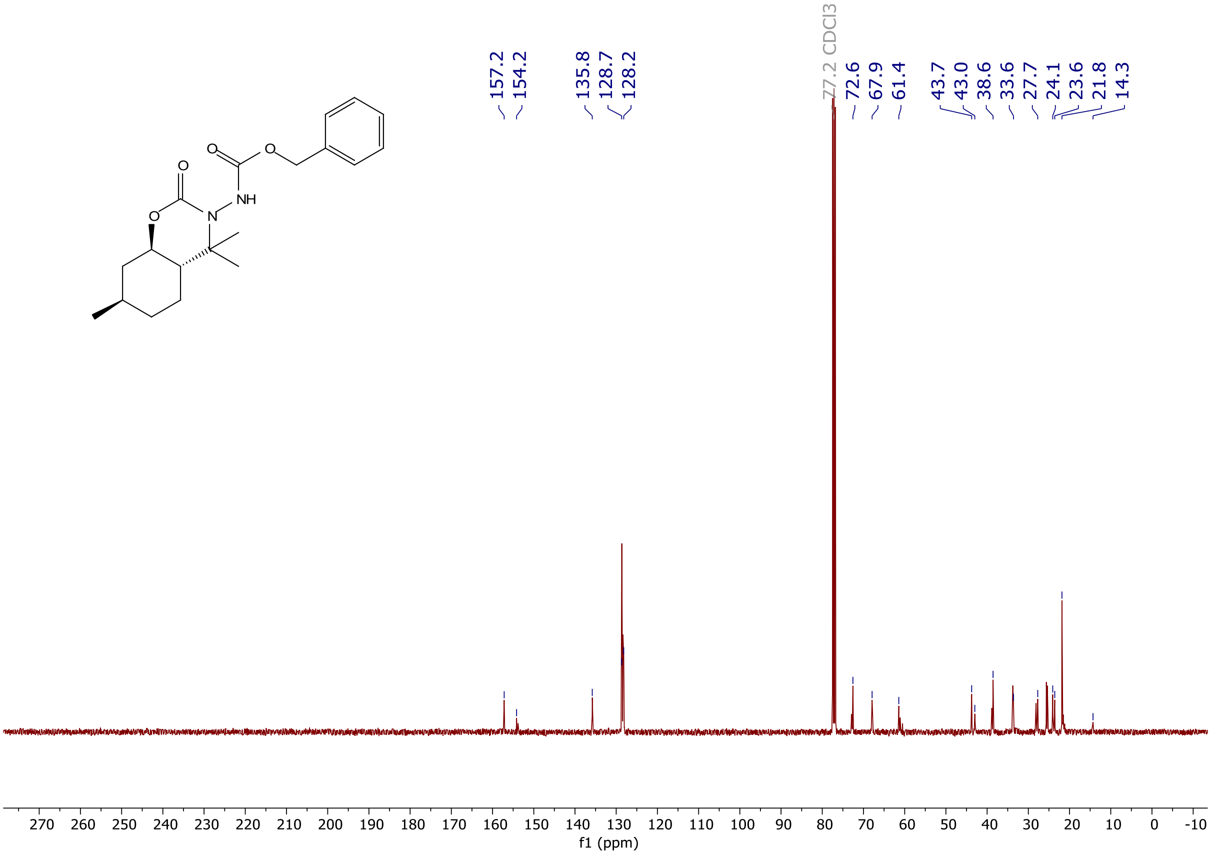

^{13}C NMR Spectrum of **2q** (101MHz, $CDCl_3$)

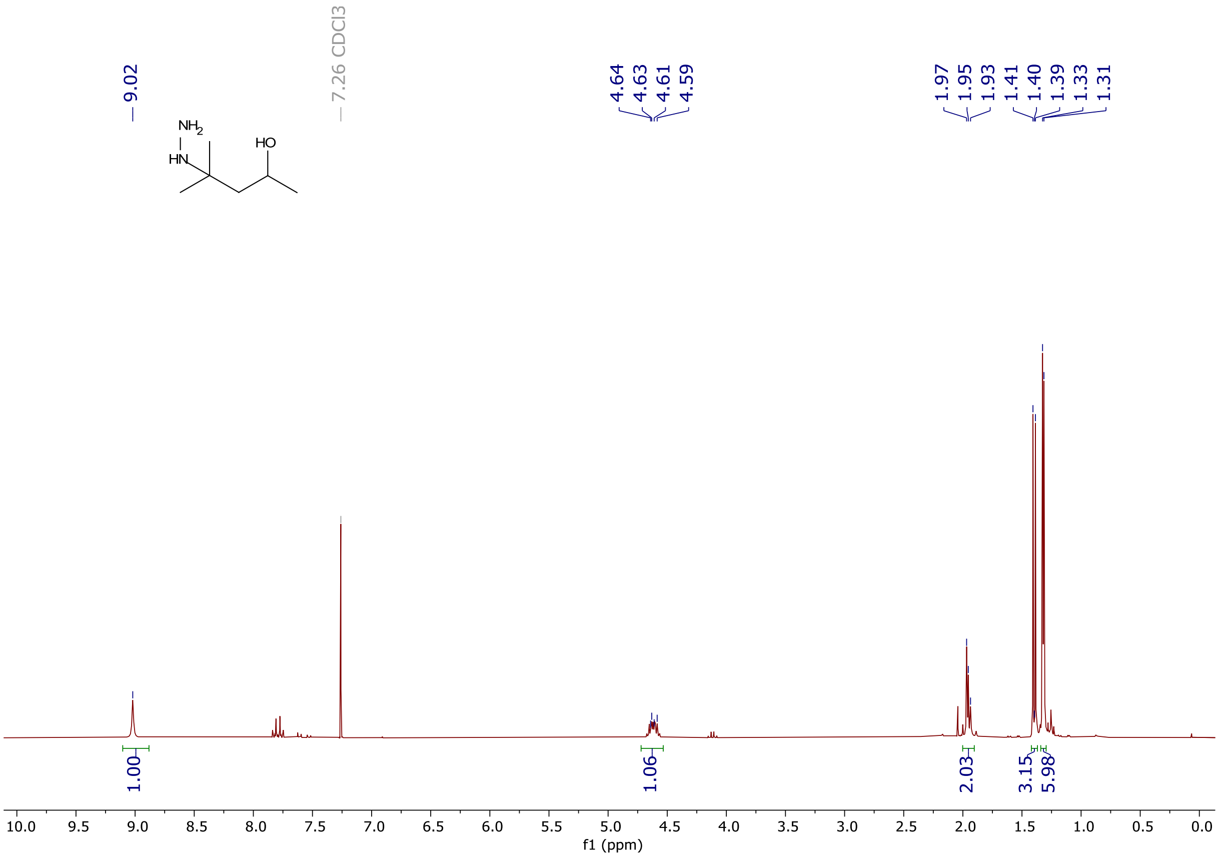

^{1}H NMR Spectrum of **3a** (300MHz, $CDCl_3$)

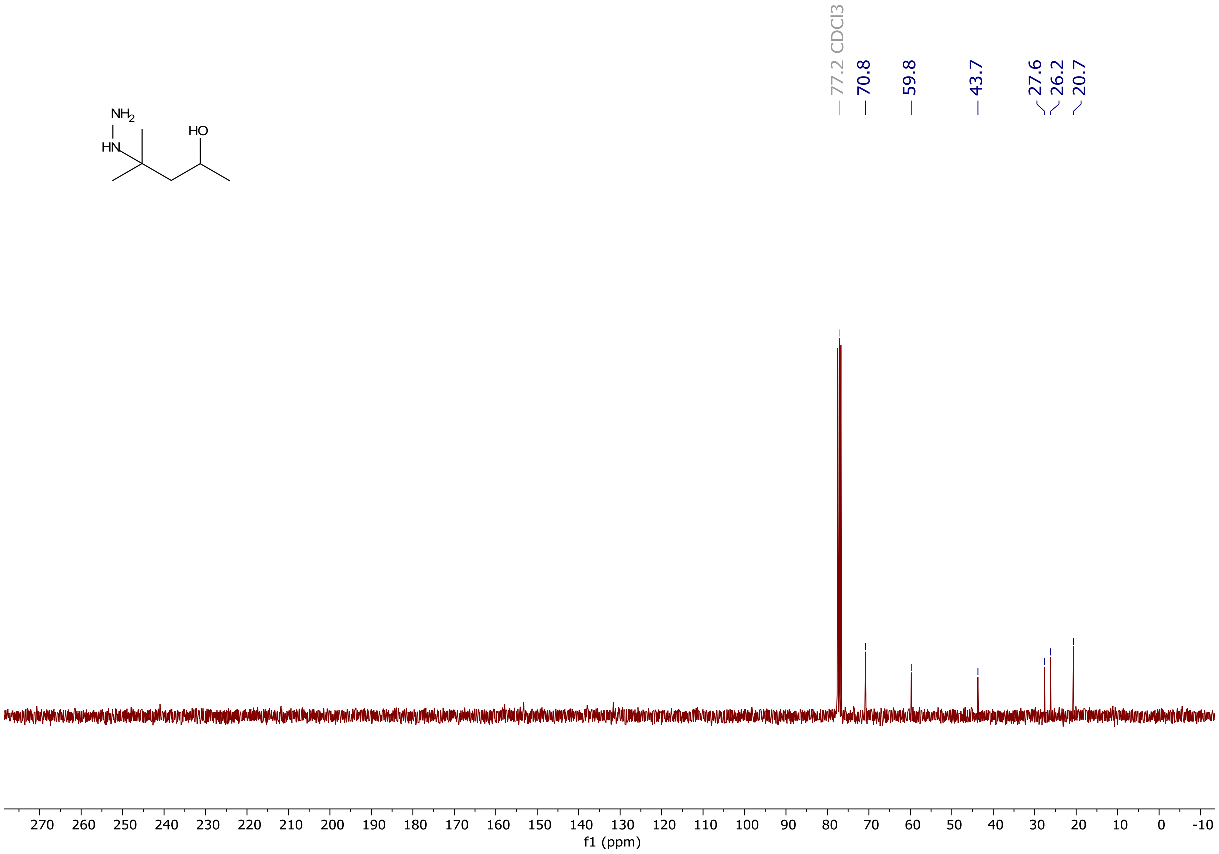

^{13}C NMR Spectrum of **3a** (101MHz, $CDCl_3$)

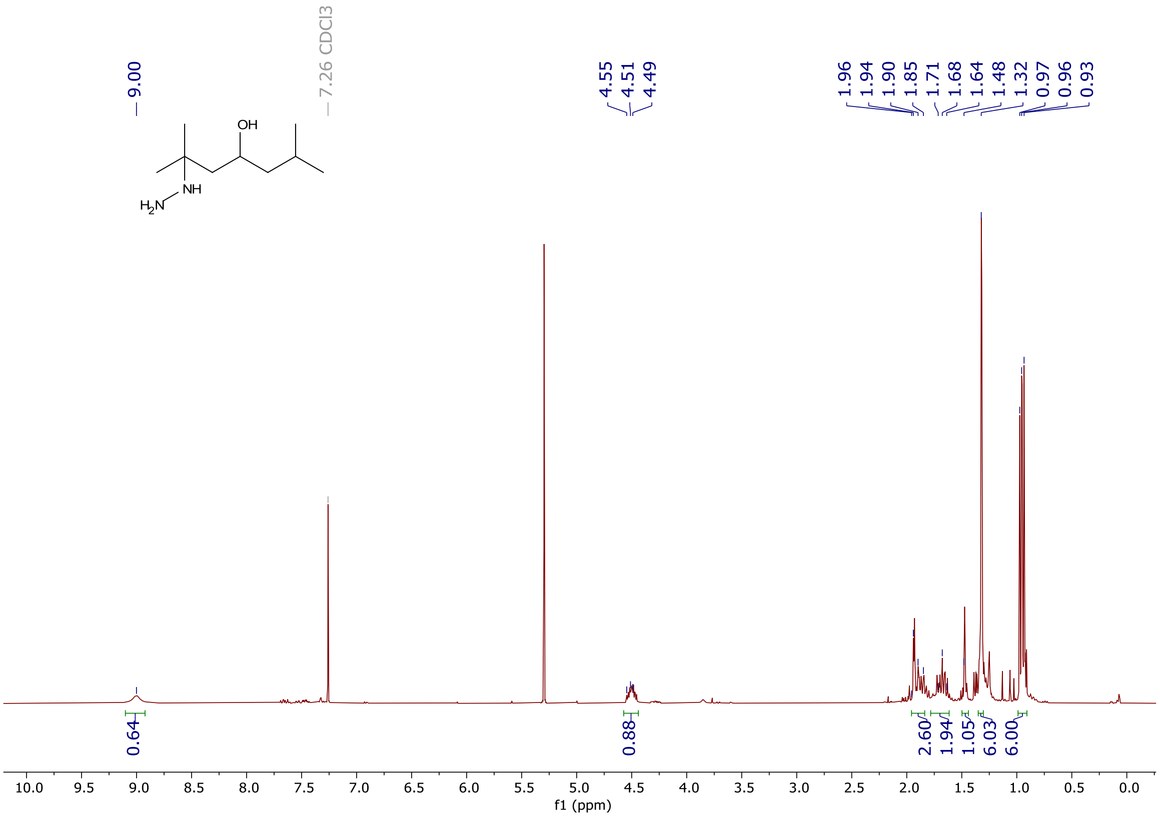

^{1}H NMR Spectrum of **3b** (300MHz, $CDCl_3$)

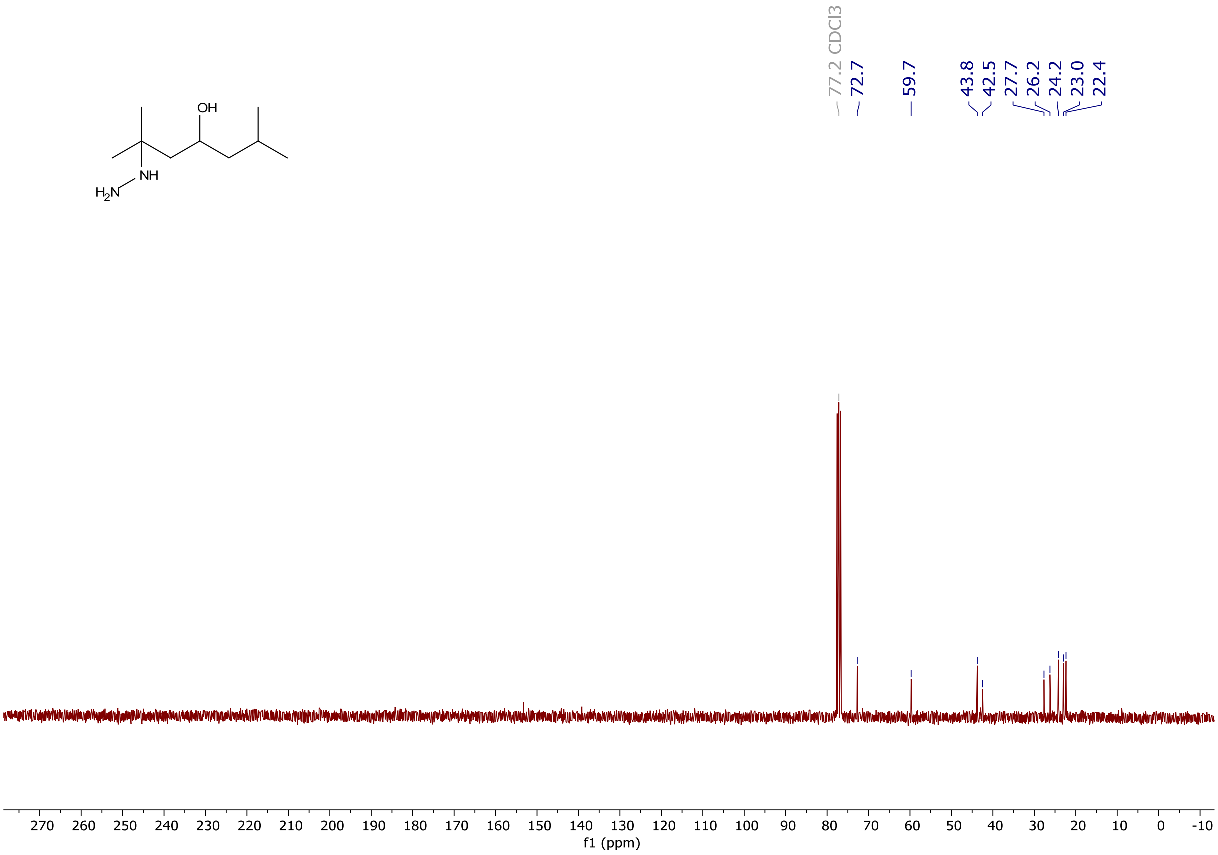

^{13}C NMR Spectrum of **3b** (101MHz, $CDCl_3$)

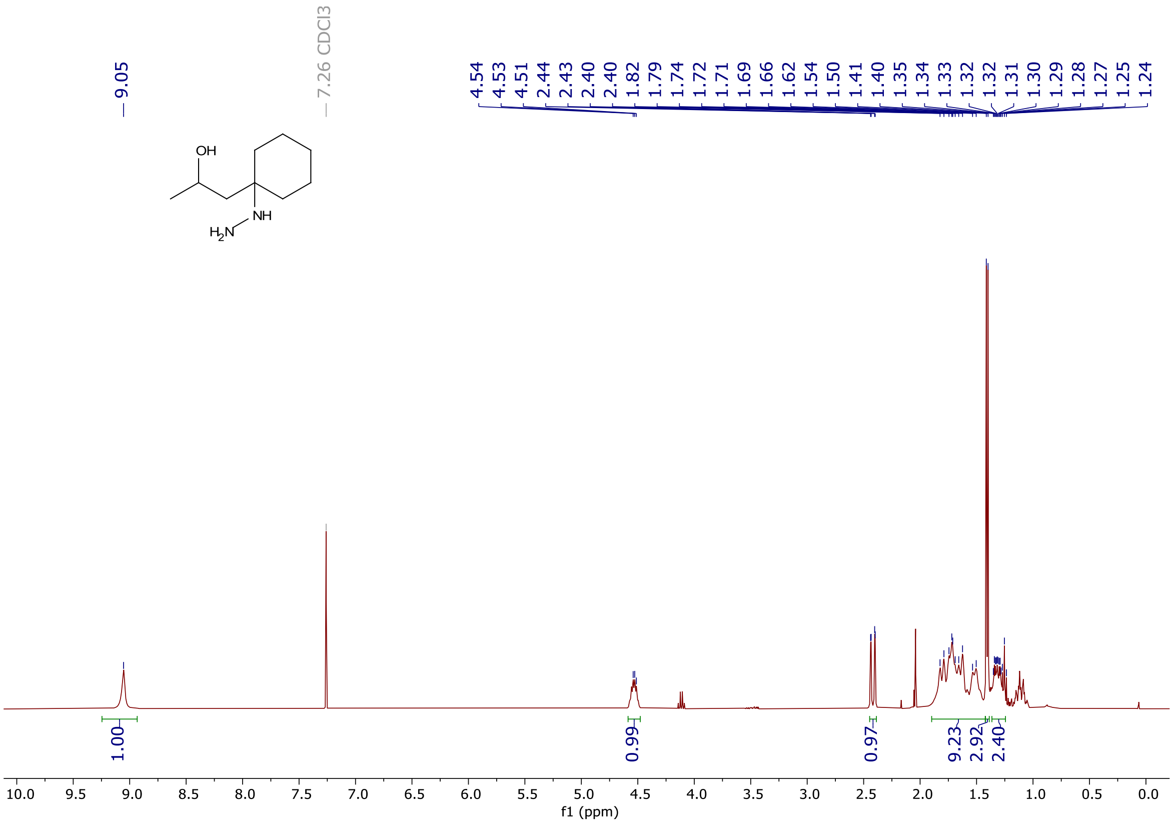

^{1}H NMR Spectrum of **3c** (300MHz, $CDCl_3$)

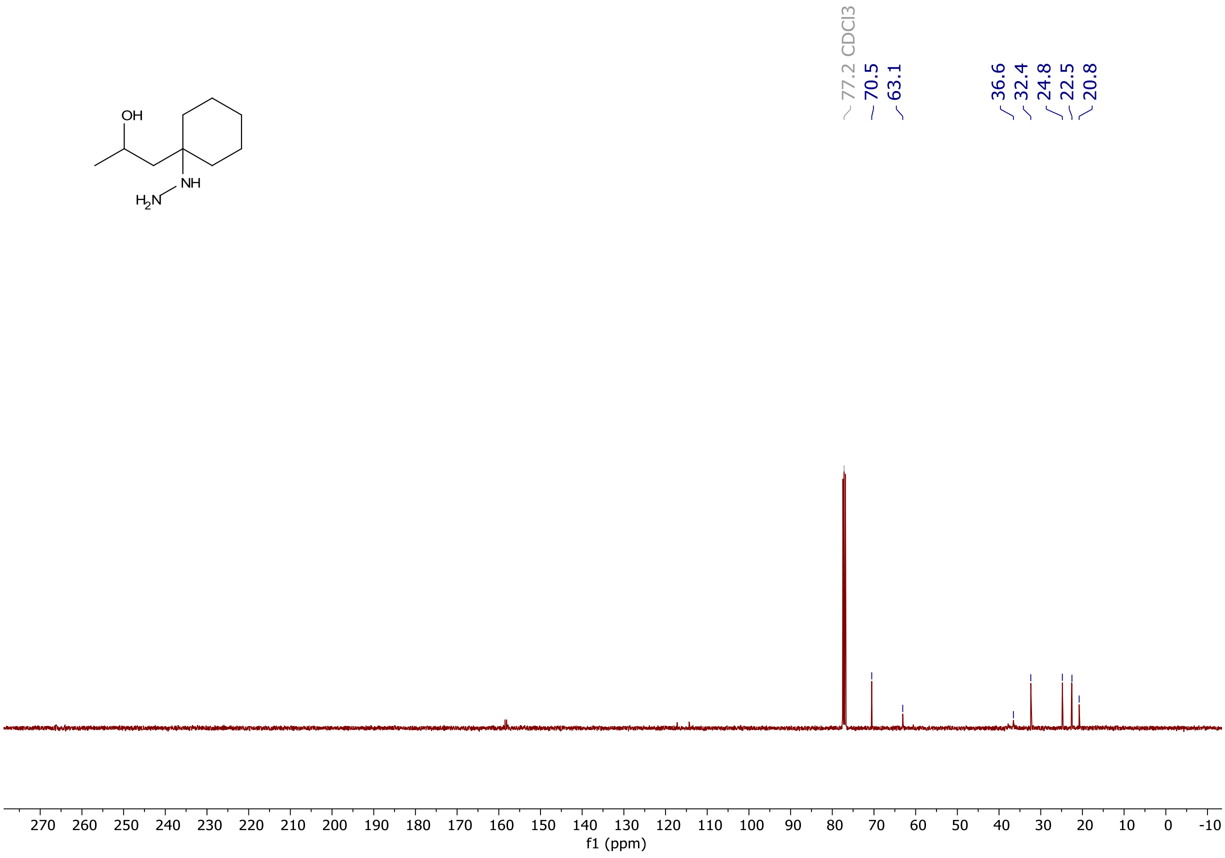

^{13}C NMR Spectrum of **3c** (101MHz, $CDCl_3$)

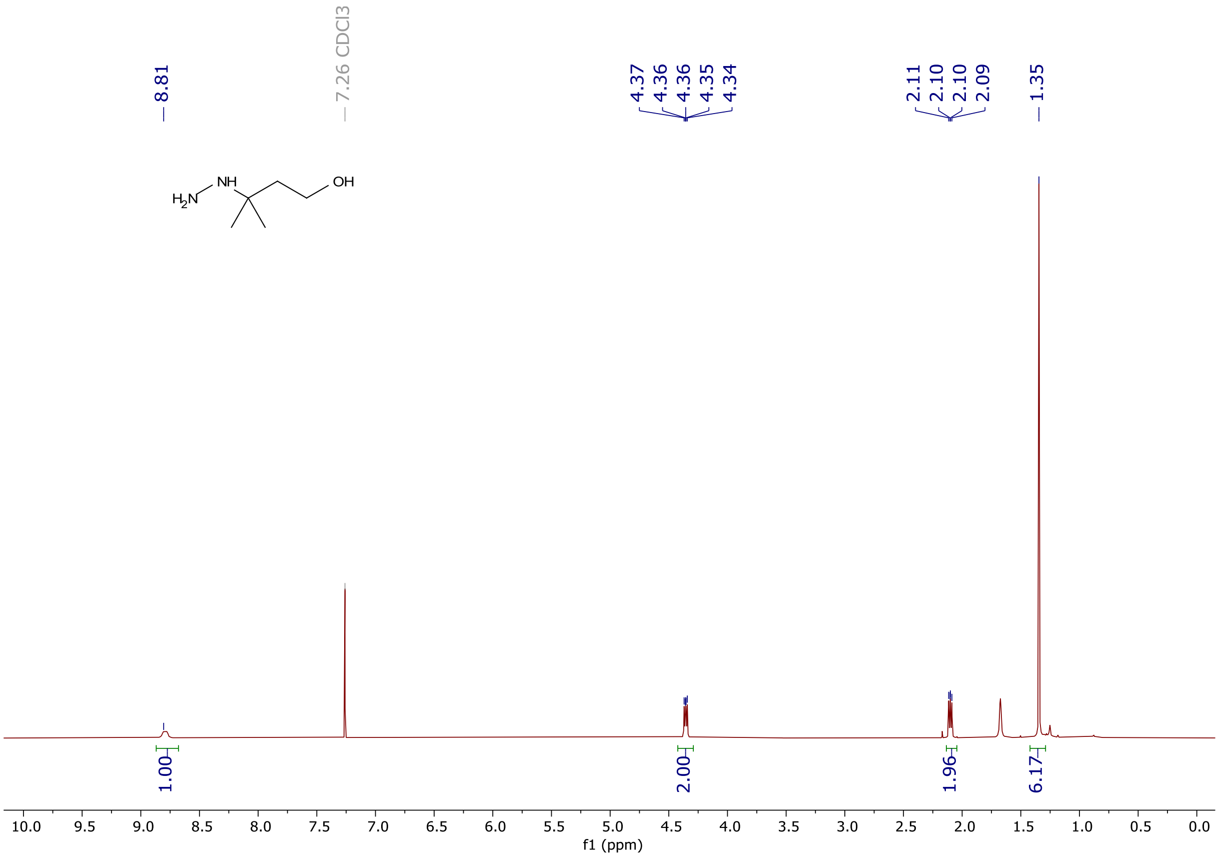

^{1}H NMR Spectrum of **3e** (300MHz, $CDCl_3$)

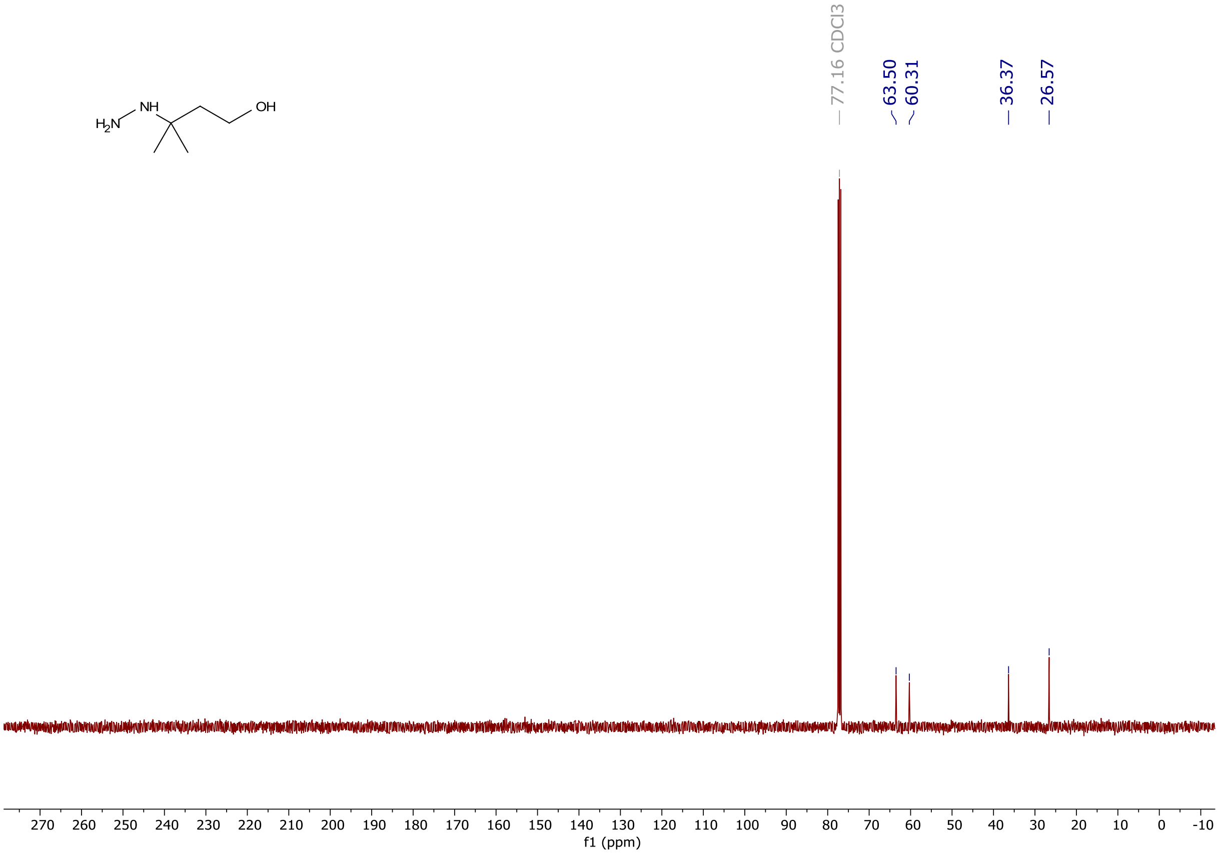

^{13}C NMR Spectrum of **3e** (101MHz, $CDCl_3$)

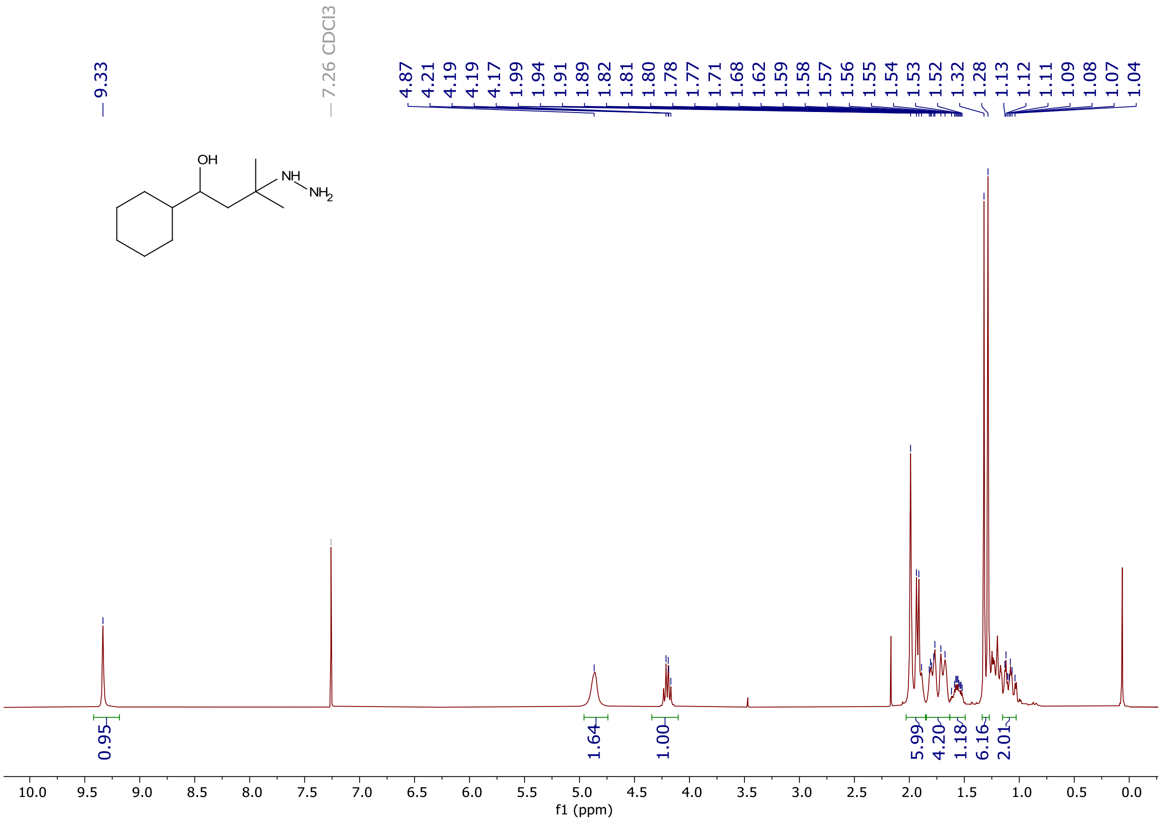

^{1}H NMR Spectrum of **3j** (300MHz, $CDCl_3$)

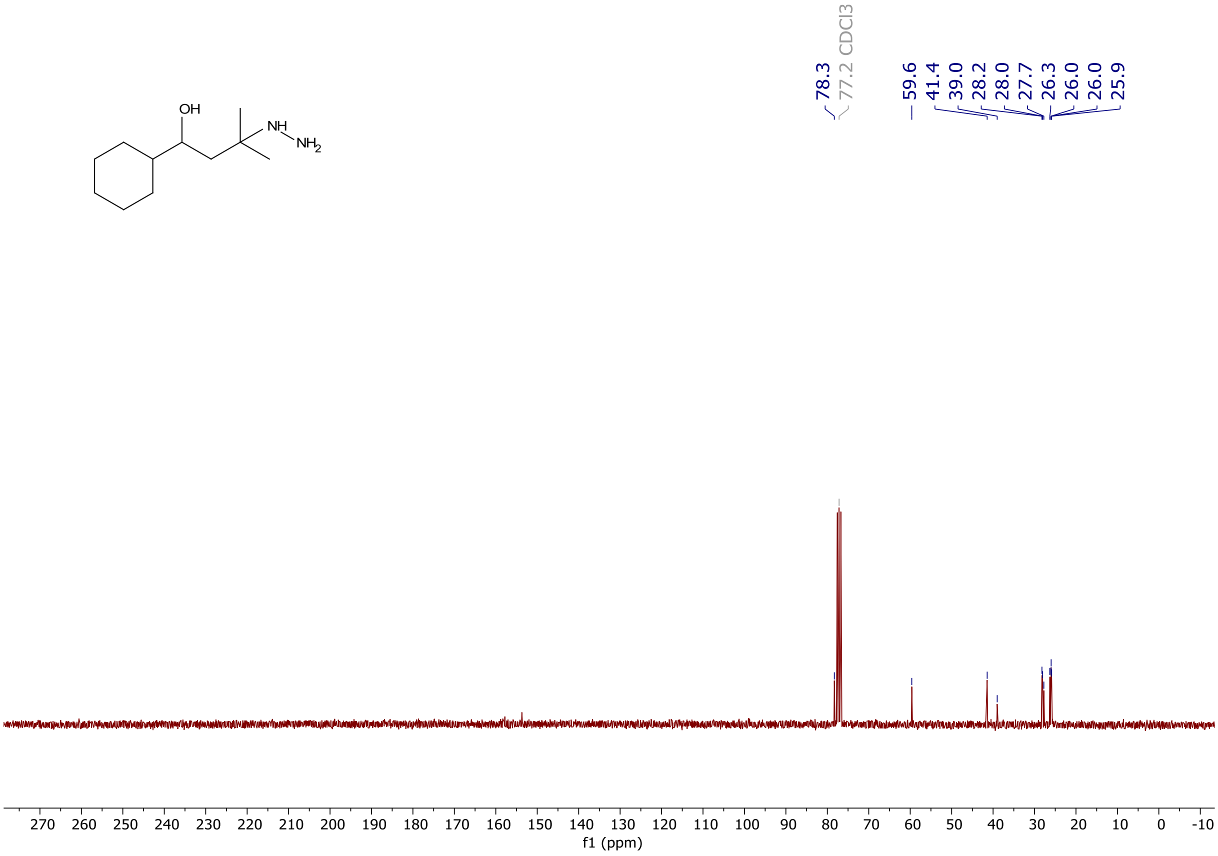

^{13}C NMR Spectrum of **3j** (101MHz, $CDCl_3$)

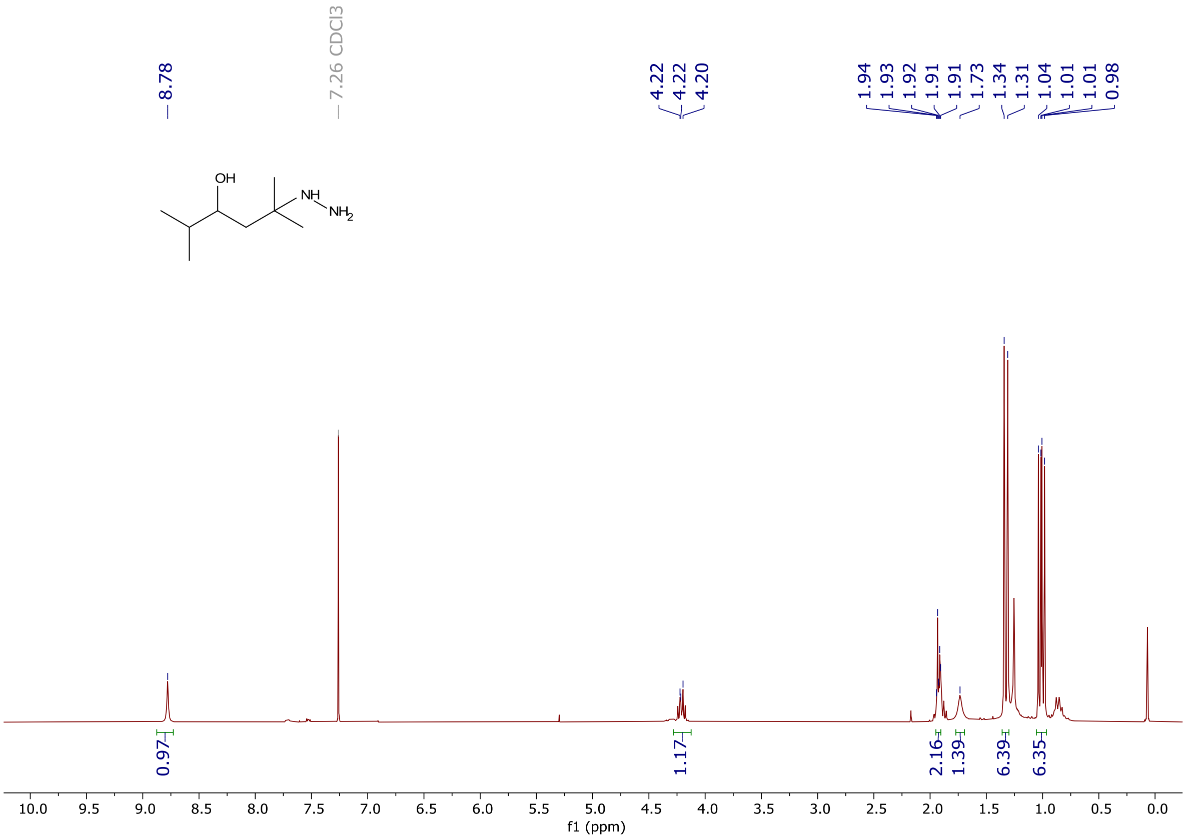

^{1}H NMR Spectrum of **3l** (300MHz, CDCl$_3$)

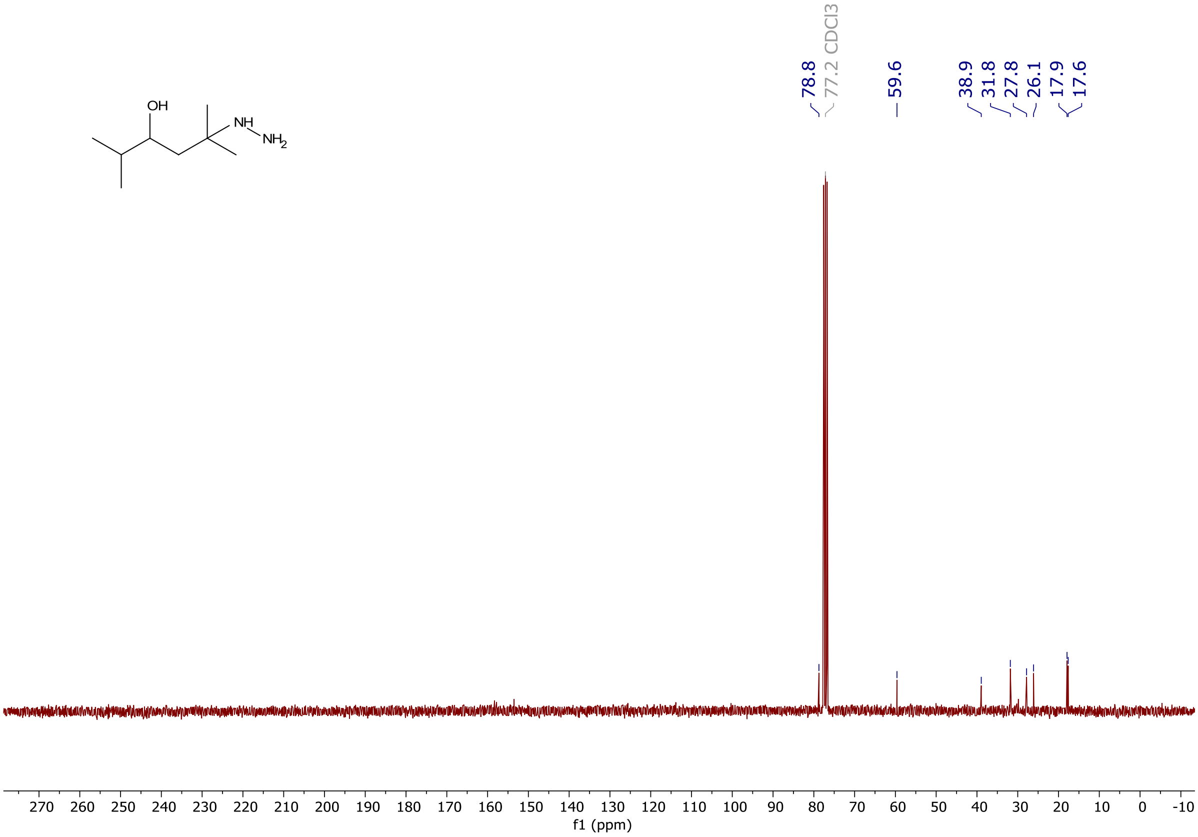

^{13}C NMR Spectrum of **3l (**101MHz, CDCl$_3$)

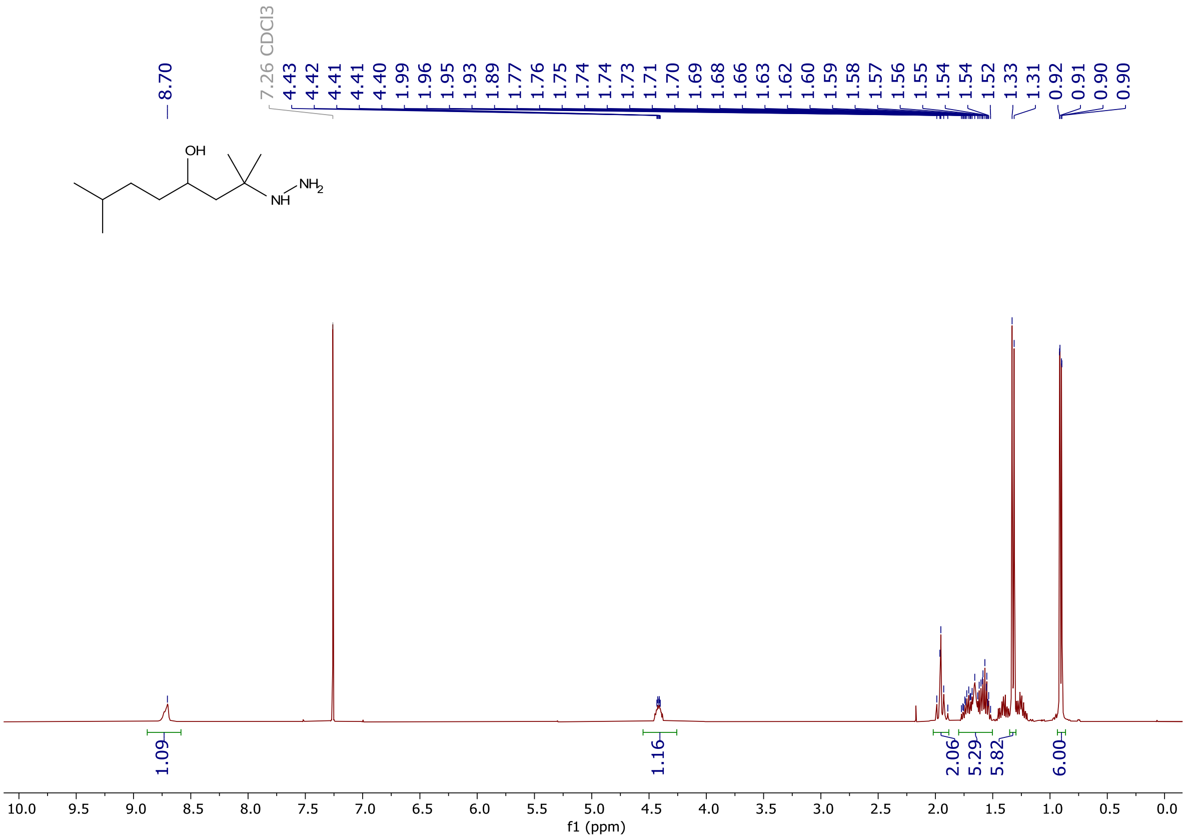

^{1}H NMR Spectrum of **3m** (300MHz, $CDCl_3$)

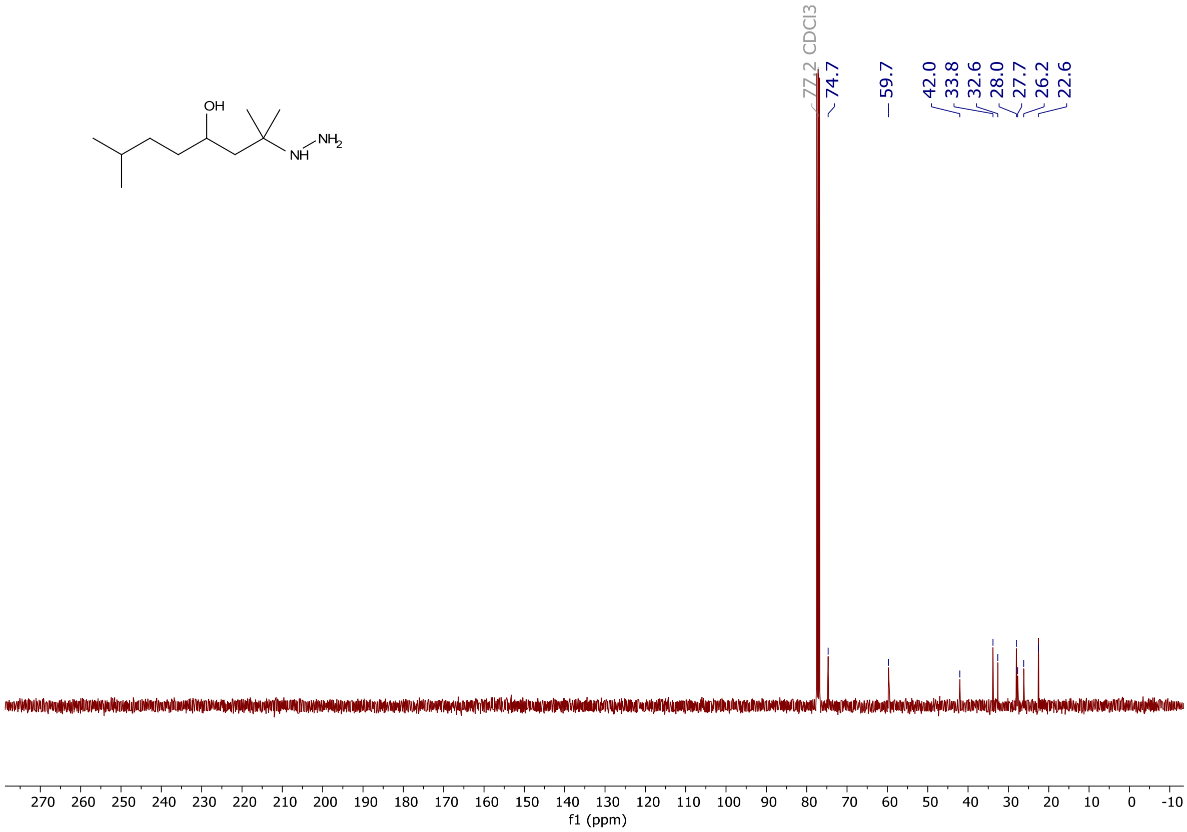

^{13}C NMR Spectrum of **3m** (101MHz, $CDCl_3$)

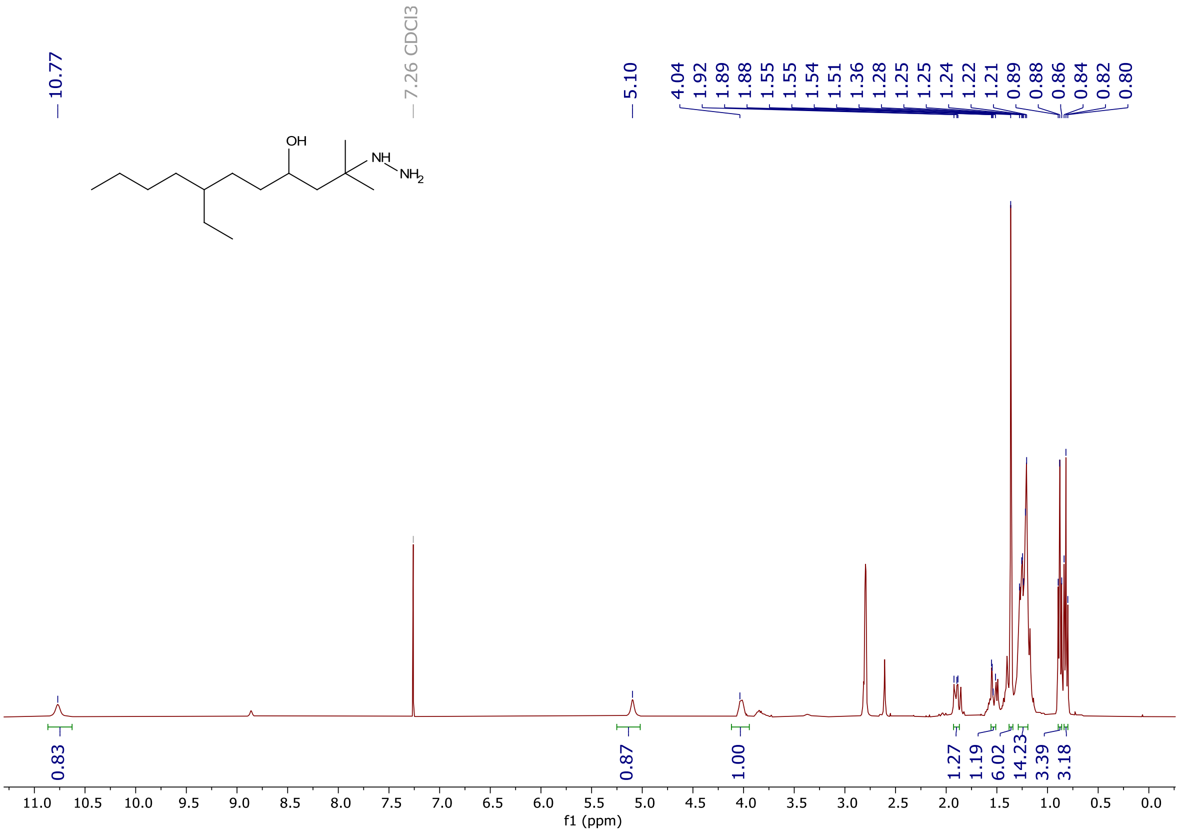

^{1}H NMR Spectrum of **3n** (300MHz, $CDCl_3$)

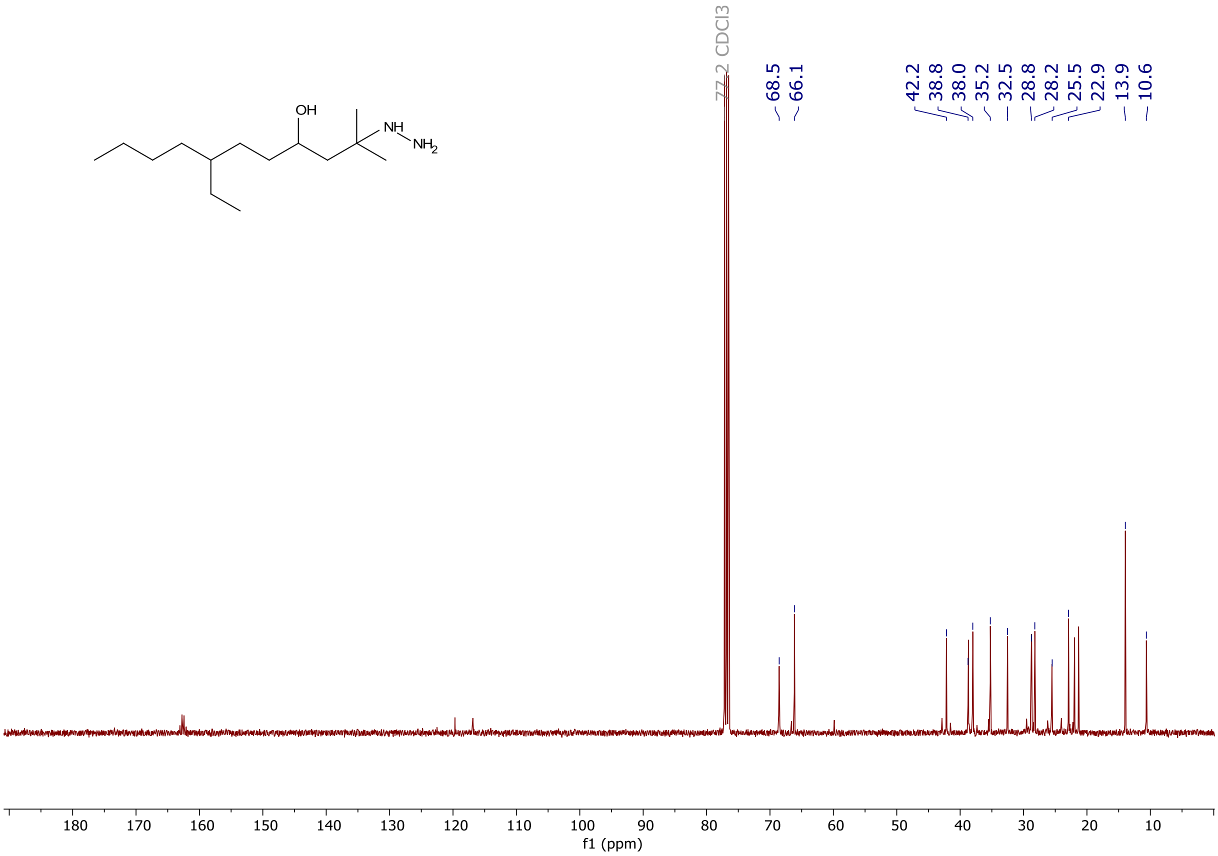

^{13}C NMR Spectrum of **3n (**101MHz, $CDCl_3$)

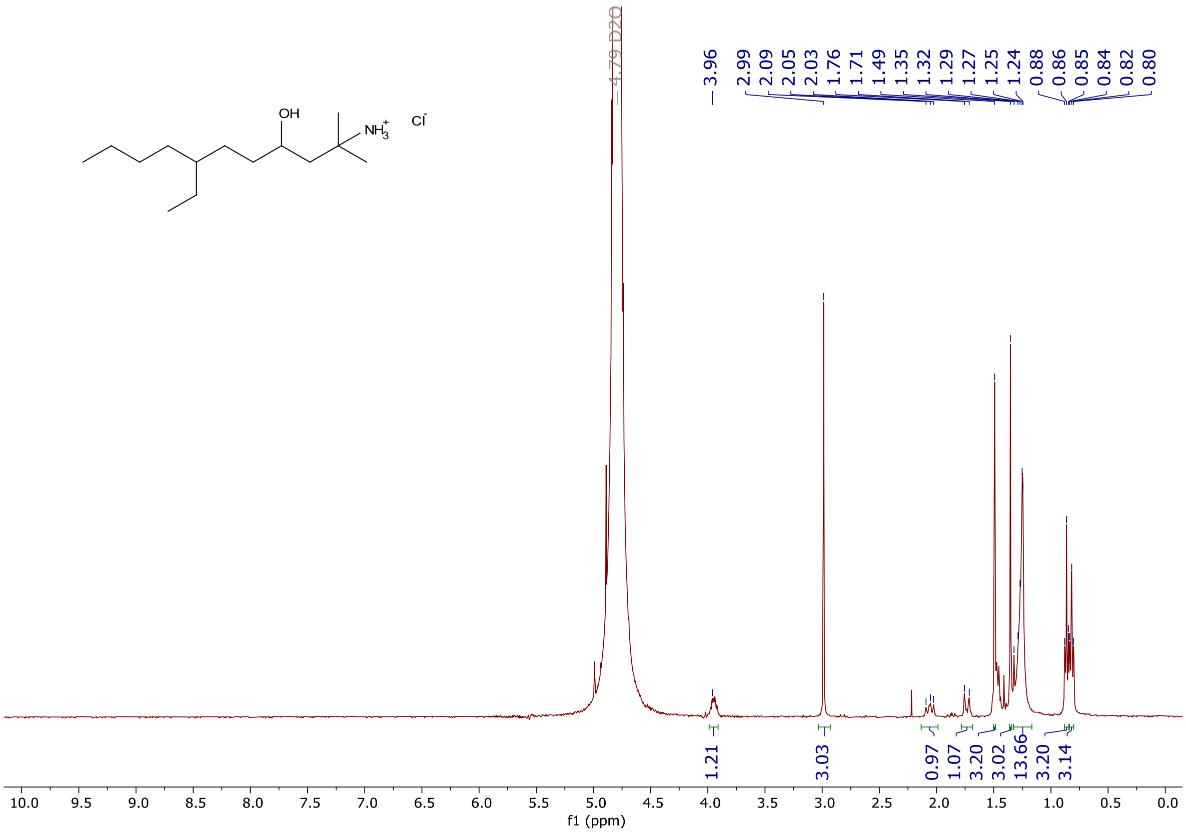

^{1}H NMR Spectrum of **4n** (300MHz, D_2O)

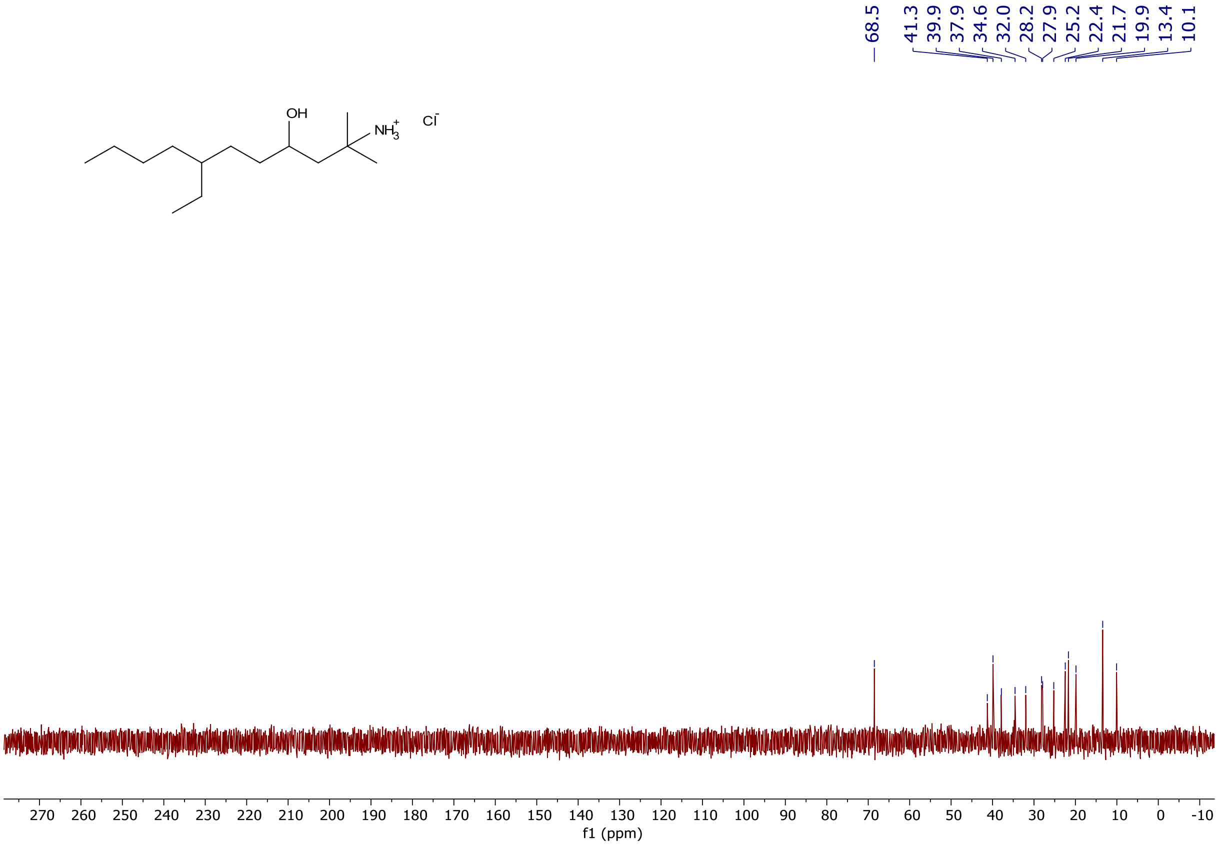

^{13}C NMR Spectrum of **4n** (101MHz, D_2O)
